# Towards Anticancer and Antibacterial Agents: Design and Synthesis of 1,2,3-Triazol-quinobenzothiazine Derivatives

**DOI:** 10.3390/ijms241713250

**Published:** 2023-08-26

**Authors:** Ewa Kisiel-Nawrot, Dominika Pindjakova, Malgorzata Latocha, Andrzej Bak, Violetta Kozik, Kinga Suwinska, Alois Cizek, Josef Jampilek, Andrzej Zięba

**Affiliations:** 1 Department of Organic Chemistry, Faculty of Pharmaceutical Sciences in Sosnowiec, Medical University of Silesia, Jagiellońska 4, 41-200 Sosnowiec, Poland; e_kisiel@o2.pl; 2 Department of Analytical Chemistry, Faculty of Natural Sciences, Comenius University, Ilkovicova 6, 842 15 Bratislava, Slovakia; pindjakova.dominika@gmail.com; 3 Department of Cell Biology, Faculty of Pharmaceutical Sciences in Sosnowiec, Medical University of Silesia, Jedności 9, 41-200 Sosnowiec, Poland; mlatocha@sum.edu.pl; 4 Institute of Chemistry, University of Silesia, Szkolna 9, 40-006 Katowice, Poland; andrzej.bak@us.edu.pl (A.B.); violetta.kozik@us.edu.pl (V.K.); 5 Faculty of Mathematics and Natural Sciences, Cardinal Stefan Wyszyński University, K. Woycickiego 1/3, 01-938 Warszawa, Poland; k.suwinska@uksw.edu.pl; 6 Department of Infectious Diseases and Microbiology, Faculty of Veterinary Medicine, University of Veterinary Sciences Brno, Palackeho tr. 1946/1, 612 42 Brno, Czech Republic; cizeka@vfu.cz; 7 Institute of Neuroimmunology, Slovak Academy of Sciences, Dubravska Cesta 9, 845 10 Bratislava, Slovakia

**Keywords:** phenothiazine, azaphenothiazines, antibacterial activity, anticancer activity

## Abstract

In this paper, we describe a new method for synthesizing hybrid combinations of 1,2,3-triazoles with a tetracyclic quinobenzothiazinium system. The developed approach allowed for the production of a series of new azaphenothiazine derivatives with the 1,2,3-triazole system in different positions of the benzene ring. In practice, the methodology consists of the reaction of triazole aniline derivatives with thioquinanthrenediinium *bis*-chloride. The structure of the products was determined by ^1^H-NMR, ^13^C-NMR spectroscopy, and HR-MS spectrometry, respectively. Moreover, the spatial structure of the molecule and the arrangement of molecules in the crystal (unit cell) were determined by X-ray crystallography. The anticancer activity profiles of the synthesized compounds were tested in vitro against human cancer cells of the A549, SNB-19, and T47D lines and the normal NHDF cell line. Additional tests of antibacterial activity against methicillin-sensitive and methicillin-resistant *staphylococci*, vancomycin-sensitive and vancomycin-resistant *enterococci*, and two mycobacterial strains were also performed. In fact, the dependence of anticancer and antibacterial activity on the substituent type and its position in the quinobenzothiazinium system was observed. Furthermore, the distance-guided property evaluation was performed using principal component analysis (PCA) and hierarchical clustering analysis (HCA) on the pool of the calculated descriptors. Finally, the theoretically approximated partition coefficients (clogP) were (inter-)correlated with each other and cross-compared with the empirically specified logP_TLC_ parameters.

## 1. Introduction

Phenothiazine is an important structural motif in medicinal chemistry, showing various biological properties; therefore, phenothiazine derivatives are an excellent example of how small structural changes in the molecule can affect host-guest interactions [1,2]. In other words, a ‘fragile event’ might occur when even a tiny structural modification can boost or completely demolish the biological activity in QSAR/QSPR studies (termed ‘magic methyl’) [3]. In practice, phenothiazine derivatives were the first neuroleptic drugs effectively applied due to the presence of an aminoalkyl substituent on the thiazine nitrogen atom, where the distance between the thiazine nitrogen atom and the nitrogen atom of the substituent was three carbon atoms [4]. It was revealed that reducing the above length to two carbon atoms changes the activity from neuroleptic to antihistaminic. As a matter of fact, many different phenothiazine derivatives with interesting biological properties, such as anticancer, antibacterial, anti-inflammatory, and antiviral, have been described so far [5,6,7]. The structures of the synthesized derivatives were modified by introducing a variety of substituents to the thiazine nitrogen atom or swapping one or two benzene rings into nitrogen heterocyclic systems, which led to the corresponding azaphenothiazines [8,9]. On the other hand, there are only a few reports on the introduction of substituents on benzene rings or heterocyclic rings.

**Figure 1:**
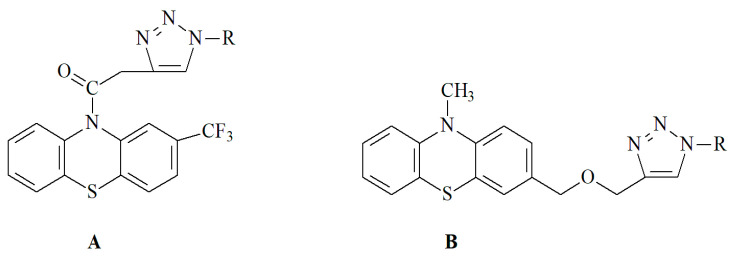
1,2,3-triazol-phenothiazine derivatives. (**A**) Phenothiazine derivatives with a 1,2,3-thiazole substituent on the thiazine nitrogen atom (**B**) Phenothiazine derivatives with a 1,2,3-thiazole substituent on the benzene ring.

1,2,3-triazoles play a crucial role not only in organic chemistry, but also in medicinal chemistry due to their relevant chemical and biological properties and the ease with which they can be synthesized by click chemistry [10]. 1,2,3-triazoles indicate high aromatic stabilization since they are highly stable under alkaline and acidic hydrolysis as well as under reducing and oxidizing conditions [11]. In fact, such heterocyclic compounds have a high dipole moment and are capable of forming hydrogen bonds (HBs), which are valid in modulating the availability and solubility of biologically active compounds—which is beneficial in binding to biomolecular targets. It should also be emphasized that the triazole ring may act as a biological linker because it exhibits bioisosteric properties with various heteroaromatic and aromatic rings [12,13,14,15]. In consequence, the 1,2,3-triazole scaffold is one of the key structural units found in molecules with diverse biological activity, such as antifungal agents [16], bacterial agents [17,18], antiallergic agents [19], antiviral agents [20,21], antituberculous agents [22,23] and anti-inflammatory agents [24]. Obviously, there are marketed drugs containing a 1,2,3-triazole structural unit, e.g., tazobactam and cefatrizine [25]. Moreover, phenothiazine derivatives with 1,2,3-triazole substituents at the thiazine nitrogen atom (see Figure 1A) were also described as showing cytotoxic properties [26]. In practice, reaction products with a 1,2,3-triazole group in position 4 of the phenothiazine system (see Figure 1B) were synthesized and characterized [27].

The details of the preparation of quinobenzothiazinium derivatives that revealed their noteworthy biological properties were presented in our previous reports [28,29,30]. The direction and strength of the antibacterial and anticancer activity depended on the presence and nature of the substituents in the tetracyclic quinobenzothiazinium system. The obtained results indicated that the anticancer effect of quinobenzothiazines may be due to DNA intercalation [31,32]. Furthermore, the comprehensive procedure of implementing different types of substituents into the 9-, 10-, and 11-positions of the quinobenzothiazinium system was reported [33], revealing additionally a dependence of antibacterial and anticancer activity both on the type of substituent and its position in the quinobenzothiazinium system.

In the current work, a new methodology for the synthesis of hybrid combinations of 1,2,3-triazoles with a tetracyclic quinobenzothiazinium system was proposed in order to obtain a series of new azaphenothiazine derivatives, where the 1,2,3-triazole motif is attached to different positions of the benzene ring using the methoxylene linker. In practice, the laboratory procedure relies on the reaction of triazole aniline derivatives with thioquinanthrenediinium *bis*-chloride. It should be emphasized that triazole quinobenzothiazinium derivatives cannot be synthesized directly using propargyl quinobenzothiazinium derivatives with organic azides. Moreover, the spatial geometry of the new products and the composition of atoms in the crystal (unit cell) were determined using X-ray diffraction. The biological potency (anticancer and antibacterial activities) of the obtained triazole quinobenzothiazinium derivatives was examined as a function of 1,2,3-triazole chemical character and the three-dimensional arrangement of substituents (their isomeric forms) in a tetracyclic quinobenzothiazinium system. 

The distance-related similarity analysis of structurally similar compounds is a common practice that contributes noticeably to the quantitative and/or qualitative structure-activity (SAR) mapping. The chemical composition (topology and/or topography) can be structurally coded by the calculated multidimensional (mD) descriptors and/or represented by the experimental property data. Accordingly, SAR-related exploration of the descriptor-based feature/structural chemical space (CS) seems valid to the quantitative potency modeling and ADMET-tailored property prediction. It is expected that in silico mapping of molecular descriptors that is combined with the experimental findings can support the synthetic efforts at the decision-making phases of hit → lead → drug design. Hence, the distance-guided property evaluation was performed using principal component analysis (PCA) and hierarchical clustering analysis (HCA) on the pool of the calculated descriptors. Moreover, the theoretically approximated partition coefficients (clogP) were (inter-)correlated with each other and cross-compared with the empirically specified logP_TLC_ parameters.

## 2. Results and Discussion

### 2.1. Chemistry—Design and Synthesis

**Figure 2:**
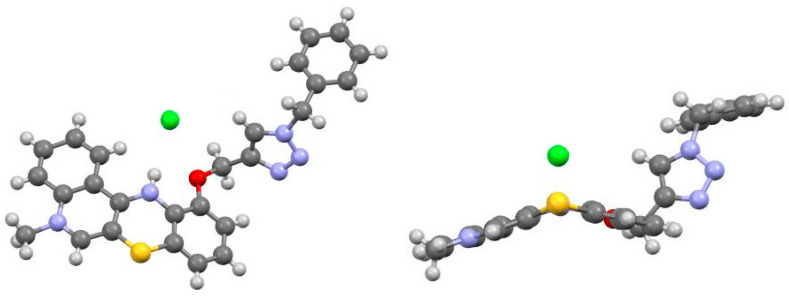
X-ray structure of 5-methyl-11-(1-benzyl-1*H*-1,2,3-triazol-4-yl) methoxy-12*H*-quino [3,4-*b*][1,4] benzothiazinium chloride **2f**.

The detailed synthesis of phenothiazine and azaphenothiazine derivatives containing 1,2,3-triazole substituents at the thiazine nitrogen atom is known; however, there are only a few reports on analogs that have attached different groups to other positions of the phenothiazine or azaphenothiazine system. In our previous paper, we described the procedure for synthesizing quinobenzothiazinium derivatives substituted in the benzene ring [33]. In fact, the synthesized compounds showed a range of interesting antibacterial properties with very low toxicity to normal human cells under in vitro culture conditions. A compelling (repeatable) dependence of the antibacterial properties on the structure of the tested compounds was revealed as well. Such findings encouraged us to continue efforts in the search for biologically active factors that determine the activity profile of these compounds. Among the generated molecules were derivatives with a propargyl group attached at different positions (9, 10, and 11) of the tetracyclic quinobenzothiazinium system. It seemed that these compounds might be a suitable substrate (starting point) for obtaining hybrid compounds with a 1,2,3-triazole system and a tetracyclic azaphenothiazinium system—a unique structure where the triazole system is attached not to the thiazine nitrogen atom, but to the carbon atoms of the benzene ring. The text continues here (Figure 2).

**Scheme 1:**
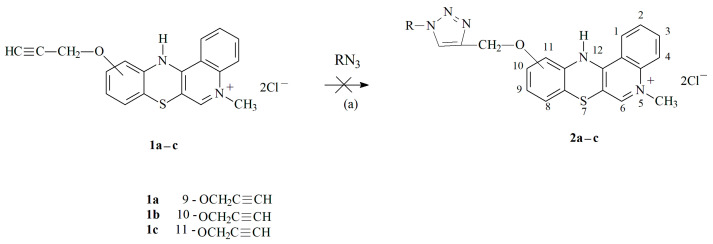
Reaction of propargyl quinobenzothiazinium derivative **1** with organic azides. Reagents and conditions: (a) DMF/H_2_O, CuSO_4_ × 5H_2_O, sodium ascorbate, room temp.

**Scheme 2:**
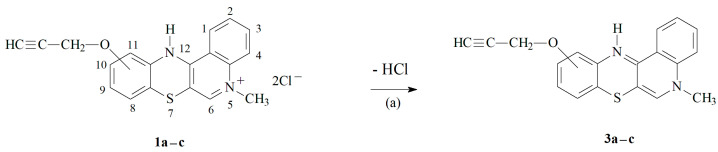
Synthesis of quinobenzothiazine derivative **3**. Reagents and conditions: (a) 5% NaHCO_3_/H_2_O, room temp.

The reactions were carried out in DMF/H_2_O (see Scheme 1) in the presence of a copper catalyst (CuSO_4_ × 5H_2_O, sodium ascorbate) at room temperature. However, the analysis of the obtained postreaction mixtures by MS mass spectrometry did not show the presence of or even trace amounts of the expected triazole quinobenzothiazinium derivative **2**. Compound **1** easily splits hydrogen chloride into the corresponding imine derivative **3**, as shown in Scheme 2. In order to exclude the possibility that the hydrogen chloride formed in the reaction medium could hinder the reactions of propargyl derivatives with azides, we decided to obtain product **3** and then carry out reaction tests with azides.

Compound **3** was prepared by treating aqueous solutions of derivative **1** with a 5% sodium bicarbonate solution. Subsequently, the obtained derivative **3** reacted with azides in analogical reaction conditions to form compound **1**. Unfortunately, the analysis of the obtained postreaction mixtures by MS mass spectrometry did not show the presence of or even trace amounts of the expected triazole quinobenzothiazinium derivatives as well. Such results made us decide to introduce the triazole moiety in a different reaction path, as was described in the synthesis of propargyl aniline derivatives [33]. We decided to use these compounds to synthesize aniline 1,2,3-triazole derivatives, which can be substrates in the preparation of 1,2,3-triazole quinobenzothiazinium derivatives.

**Scheme 3:**
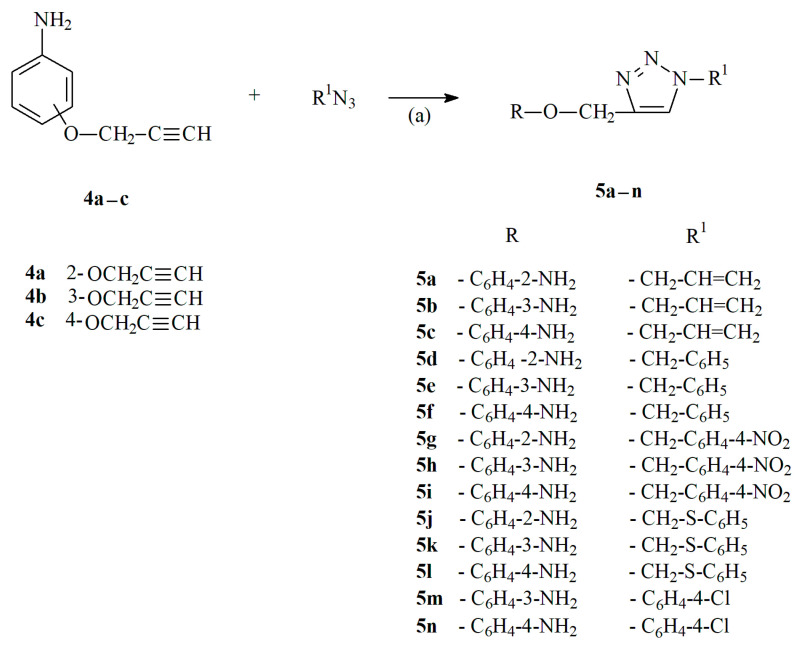
Synthesis of aniline derivatives of 1,2,3-triazoles **5**. Reagents and conditions: (a) DMF/H_2_O, CuSO_4_ × 5H_2_O, sodium ascorbate.

We carried out the reactions in DMF/H2O in the presence of a copper catalyst (CuSO_4_ × 5H_2_O, sodium ascorbate), as presented in Scheme 3. As a matter of fact, commercial azides were used. In the case of the synthesis of the allyl derivatives **5a**–**c**, the allyl azide was prepared by reacting allyl bromide with sodium azide immediately *prior to* the coupling reaction with the propargyl derivative **4**. The synthesis was performed in the one-pot procedure without isolating the azide from the reaction mixture. In practice, the reactions of propargyl derivatives **4b**,**c** with aromatic azide (4-chlorophenyl azide) were conducted in a microwave reactor at 80 °C. Some of the generated aniline 1,2,3-triazole derivatives were characterized previously [34,35]. The structure of the obtained compounds was confirmed by ^1^H- and ^13^C-NMR spectroscopy and HR-MS spectrometry (see Supplementary Materials (Figures S1–S62)).

**Scheme 4:**
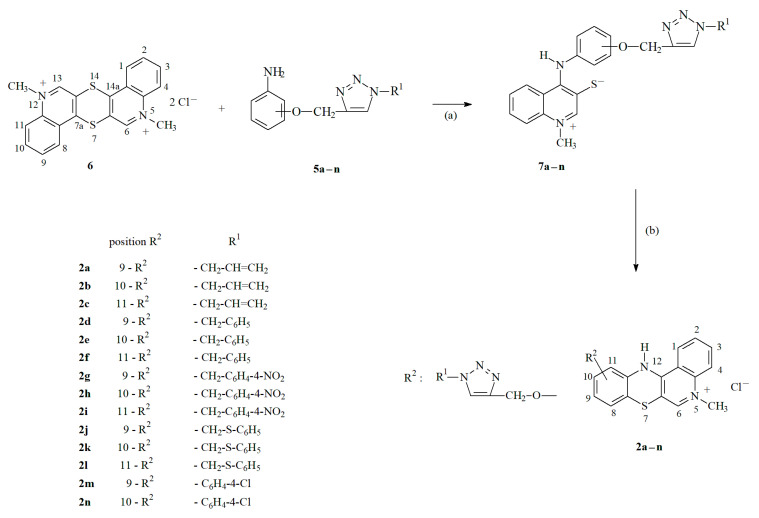
Preparation of 1,2,3-triazole quinobenzothiazinium derivatives **2a**–**n**. Reagents and conditions: (a) Py, 20 or 80 °C, (b) HCl, O_2_.

In the next stage, the reactions of 1,2,3-triazole derivatives **5a**–**n** with 5,12-(dimethyl)thioquinanthrenediinium **6** *bis*-chloride were carried out, as illustrated in Scheme 4. Similarly, to the previous reactions of *bis*-chloride **6** with aniline derivatives, the reaction was conducted in a pyridine environment, using a 2.5-fold excess of the aniline 1,2,3-triazole derivative **5a**–**n** in relation to *bis*-chloride **6**. The reaction was continued up to the complete reaction of salt **6**. The best yields were recorded when the reaction was carried out at 80 °C for 24 h. The course of the reaction and the structure of the products were dependent on the position of the substituents in the aniline ring of compounds **5a**–**n**. In the case of derivative **5**, which contains substituents at the positions *ortho* and *para* towards the amino group, the second stage of the reaction consisting of the cyclization of quinolinio-3-thiolate **7** formed in the first stage of the reaction proceeded selectively. It led to the appropriate product **2**, in which the substituents were in position 9 or 11 of the quinobenzothiazine system.

**Scheme 5:**
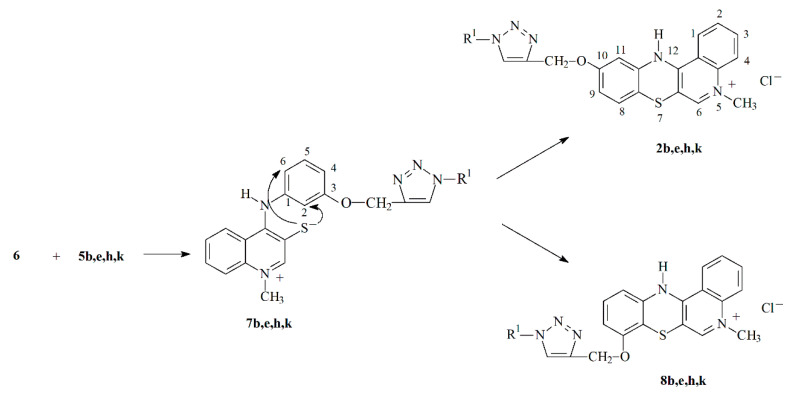
Directions of cyclization reactions of intermediate product **7**.

As shown in Scheme 5, product **7** is formed by the reaction of salt **6** with derivative **5** containing a substituent *meta* to the amino group. The cyclization reaction of compound **7** by nucleophilic substitution of a hydrogen atom with a thiolate sulfur atom might occur at position 2 or 6 of the ring on the phenylamino substituent. The reactions were carried out at 80 °C, leading to a mixture of compounds **2** and **8** containing substituents in position 8 or 10 of the quinobenzothiazinium system (the result of the hydrogen atom substitutions in both positions 2 and 6 of the phenyl ring). The composition of the resulting product mixtures was determined based on the signal integration in ^1^H-NMR spectra taken in DMSO solutions. The appropriate isomers **2** and **8** were formed in a molar ratio close to 1:3. Reaction tests of aniline 1,2,3-triazole derivatives **5b**,**e**,**h**,**k** with *bis*-chloride **6** under various temperature conditions were performed. It turned out that the reactions carried out at a temperature of 20 °C had a selective course and led to the formation of pure quinobenzothiazinium isomers **2b**,**e**,**h**,**k** that contain a substituent at position 10 of the quinobenzothiazinium system. It can be assumed that such a course of reaction could be related to the steric hindrance of the substituents in the aminophenyl group. In the case of the formation of isomers having substituents in position 8 of the quinobenzothiazine system, the cyclization of the quinoline-3-thiolate **7** proceeds by substituting a hydrogen atom between two substituents in the *meta* position to each other, which can be difficult at lower temperatures.

### 2.2. X-ray Structural Analysis

The structure of compound **2** was finally determined by X-ray crystallography. A single crystal of derivative **2f** was obtained by crystallization from ethanol. The X-ray structure of derivative **2f** is shown in Figure 2, where molecule **2f** is bent along the axis determined by the nitrogen and sulfur atoms of the thiazine ring at an angle of 145.9°. The angle formed by the C6a-S7-C7a atoms in the thiazine ring is 99.14°, while between the C11a-N12-C12a atoms, it is 121.08°. Most of the structural parameters of the tetracyclic quinobenzothiazinium system are similar to the parameters of the structure of 5-methyl-12*H*-quino [3,4-*b*][1,4]benzothiazinium chloride described earlier [9].

### 2.3. In Vitro Cytotoxic Activity

The cytotoxic activities of aniline 1,2,3-triazole derivatives **5a,f,h,i** and the 1,2,3-triazole quinobenzothiazinium derivatives **2a**–**n** against human cancer cell lines (A549, SNB-19, and T-47D) and a normal NHDF cell line are presented in Table 1 and Table 2. Measurements were conducted in triplicate (*n* = 3) and the data were reported with standard deviations (±std) calculated as the square root of variance. 

The synthesized 1,2,3-triazole **2a**–**n** quinobenzothiazinium derivatives revealed much higher anticancer activity compared to the previously analyzed quinobenzothiazinium derivatives [7], which emphasized the importance of the 1,2,3-triazole system for the cytotoxic activity of compound **2**.

**Scheme 6:**
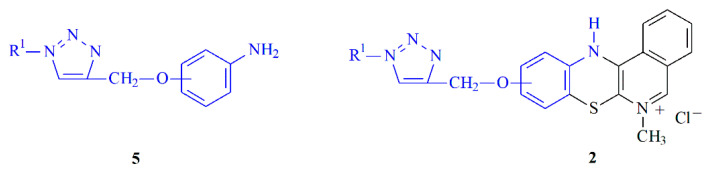
Structure of molecules **2** and **5** with a common substructure in blue.

At the same time, the cytotoxic activities of selected aniline 1,2,3-triazole derivatives **5a**,**f**,**h**,**i**, which are substrates for the preparation of compound **2**, were tested. Structural fragments are part of the molecule of the tetracyclic quinobenzothiazinium system, as illustrated in Scheme 6. It turned out that none of the tested cell lines, both cancerous and normal, was susceptible to the action of **5a**,**f**,**h**,**i** derivatives. For all tested compounds, IC_50_ values were above 100 µM. 

In fact, the key element determining the cytotoxic activity of compound **2** is the tetracyclic quinobenzothiazinium system. The SNB-19 cell line was the most susceptible to the effects of **2a**–**n** derivatives, and the T47D cell line was the least susceptible. The derivatives **2a**–**c**, containing an allyl substituent at the triazole ring, revealed very high activity against all tested cancer cell lines. 5-methyl-9-(1-allyl-1*H*-1,2,3-triazol-4-yl)methoxy-12*H*-quino [3,4-*b*][1,4] benzothiaziniu chloride (**2a**), with an IC_50_ range from 0.23 to 2.1 µM was the most active agent of all of the tested compounds. High anticancer activity was also demonstrated by 5-methyl-9-(1-benzyl-1*H*-1,2,3-triazol-4-yl)methoxy-12*H*-quino [3,4-*b*][1,4] benzothiazinium chloride (**2d**) against cell line SNB-19, 5-methyl-10-(1-allyl-1*H*-1,2,3-triazol-4-yl)methoxy-12*H*-quino [3,4-*b*][1,4] benzothiazinium chloride (**2b**) against cell lines SNB-19 and A549 and 5-methyl-11-[1-(4-nitrobenzyl)-1*H*-1,2,3-triazol-4-yl]methoxy-12*H*-quino [3,4-*b*][1,4] benzothiazinium chloride (**2i**) against the T47D cell line. Low cytotoxic activity was shown by **2m**,**n** derivatives containing a chlorophenyl substituent on the triazole ring. The **2a**–**n** derivatives revealed little cytotoxicity to normal NHDF cells. The IC_50_ of most of them was above 100 μM—only derivatives **2a**,**e**,**f**,**g**,**j** showed IC_50_ of 47.8, 56.6, 30.5, 27.6, and 28.7 μM against NHDF cells, respectively. It should also be noted that compounds **2a**–**n** had a stronger cytotoxic effect on the SNB-19 cell line than cisplatinum. The influence on the activity of both the nature of the substituents and their position in the quinobenzthothiazinium system was also observed. In the case of triazole substituents containing an allyl and benzyl group, the most active were derivatives containing a substituent in positions 9 and 10 of the quinobenzothiazinium system. On the other hand, derivatives with a *p*-nitrobenzyl and phenylthiomethyl substituent at the triazole ring do not show such a relationship.

### 2.4. In Vitro Antimicrobial Activity

All discussed compounds were tested in vitro for their antibacterial and antimycobacterial activity; therefore, the universally susceptible collection strains of *Staphylococcus aureus* ATCC 29213 and *Enterococcus faecalis* ATCC 29212 were selected. Subsequently, the efficacy was evaluated against resistant clinical isolates of human and veterinary origin, i.e., against methicillin-resistant *S. aureus* (MRSA) isolates SA 3202, SA 630, and 63718 carrying the mecA gene [36] and vancomycin-resistant *E. faecalis* (VRE) 342B, 368, and 725B isolates carrying the vanA gene [37]. Thus, the tested strains differed in the spectrum of antibiotic resistance, genetic equipment, and probably also the accessory genome. In addition, all compounds were evaluated in vitro against fast-growing *Mycobacterium smegmatis* ATCC 700084, and slow-growing *M. marinum* CAMP 5644 as a safe alternative to *M. tuberculosis* [38]. Activities are expressed as minimum inhibitory concentration (MIC) and minimum bactericidal concentration (MBC), as shown in Table 3. In order to determine whether a compound exhibits bactericidal activity against a particular test strain, it must meet the condition of MBC/MIC ≤ 4 [39]. MBC values that fulfill the above requirement (i.e., the compound is bactericidal) are reported in bold in Table 3.

Interestingly, the insertion of the triazole motif increased anticancer activity, but on the contrary, it is counterproductive for antimicrobial activity. Unfortunately, all compounds are inactive against facultative anaerobic enterococci [40], which are known for their high resistance to disinfection procedures and antibiotics [41,42]. Per contra, the compounds substituted in position 10 of the quinobenzothiazine system with either benzyl or phenylthiomethyl, e.g., 5-methyl-10-(1-benzyl-1*H*-1,2,3-triazol-4-yl)methoxy-12*H*-quino [3,4-*b*][1,4] benzothiazinium chloride (**2e**) and 5-methyl-10-(1-(phenylthio)methyl-1*H*-1,2,3-triazol-4-yl)methoxy-12*H*-quino [3,4-b][4*b*][1,4] benzothiazinium (**2k**) showed the highest antistaphylococcal activities (MICs ranged from 16.3 to 65.5 µM). From the point of view of antistaphylococcal activity, substitution with phenylthiomethyl appears to be more advantageous since the entire subseries were active. The acceptable values of activity against staphylococci were also revealed for molecule **2m** with a substitution at position 9 with chlorophenyl. Thus, it seems that the antistaphylococcal activity is primarily determined by the substituent on the triazole ring and subsequently by the position of the substituent on the quinobenzothiazine system, with position 11 and the allyl chain being the least favorable. On the other hand, the most antimycobacterially active agents were the chlorinated derivatives **2m**,**n** (MICs ranged from 8.19 to 32.7 µM), followed by **2j**–**l** (substitution with phenylthiomethyl), whereby substitution at position 9 of the tetracyclic skeleton again appears to be advantageous.

Overall, it can be summarized that rough SAR requirements for anticancer and antimicrobial activity go against each other. The anticancer activity is more pronounced than the antimicrobial one, which can be understood as a specific benefit when the drug is simultaneously able to suppress related infections arising as a result of immunodeficiency caused by cytostatic treatment. In this context, it should be noted that all of the active compounds demonstrated bactericidal activity.

Since the compounds showed more antimycobacterial than antibacterial activity, an attempt was made to estimate the mechanism of action of these compounds. Since *M. smegmatis* is particularly useful in the study of basic cellular processes of particular importance to pathogenic mycobacteria [43], a standard MTT assay with the most active derivatives was performed with it. The MTT assay can be used to assess cell growth by measuring respiration. For example, the antitubercular drug bedaquiline is a strong inhibitor of mycobacterial respiration [44]. The respiratory activity of bacterial cells (which is finally reflected in their viability) of less than 70% after exposure to the MIC values for each tested compound is considered as a positive result of this assay. This low level of cell oxidative metabolism indicates inhibition of cell growth by inhibition of respiration [45,46]. The lowest multiples of the MIC values by which the inhibition of *M. smegmatis* viability (%) greater than 70% was achieved are reported in Table 4. It can be concluded that evaluated compounds **2h**,**m**,**n** demonstrated a decrease in viability <70% at its MIC value, which suggests that their mechanism of action may be connected with inhibition of the respiratory chain.

### 2.5. Similarity-Related Property Assessment

**Figure 3:**
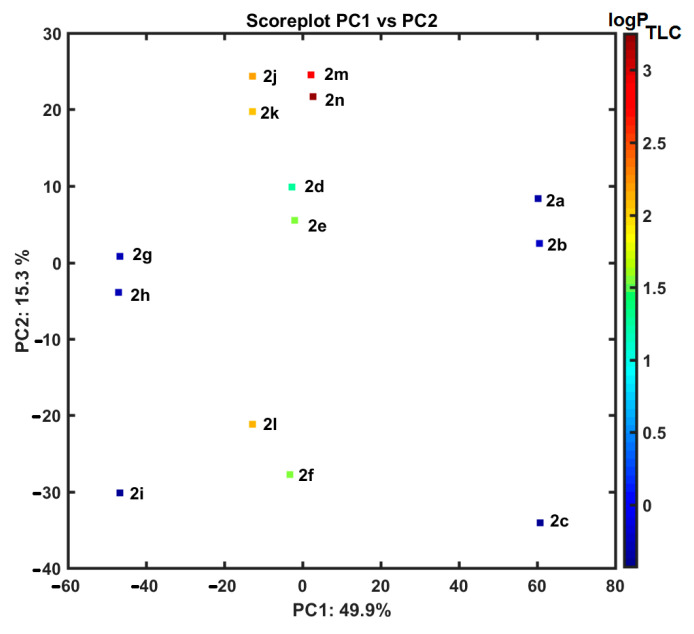
Projection of 1,2,3-triazol-quinobenzothiazine derivatives **2a**–**n** on a plane defined by PC1 and PC2. Colors code the empirical TLC lipophilicity.

The clustering potency of the descriptor-based data can be examined by tracing the (dis)similarities between objects (molecules **2a**–**n**) in the multidimensional (mD) variable space generated by Dragon 6.0 software [47]. From the initial number of selected parameters (4885), all columns with constant or nearly constant values (standard deviation < 10^−4^) and with missing values have been excluded at the preprocessing stage, resulting in the final set of 2789 descriptors. Thus, the distance-guided property evaluation was performed using the principal component analysis (PCA) and hierarchical clustering analysis (HCA) on a pool of 2789 variables, including topological, constitutional, 2D/3D, physicochemical variables. The software-retrieved descriptors were then organized into matrix X_14×2789_ with rows representing compounds **2a**–**n** (objects) and columns representing numerical parameters (descriptors). The resulting matrix was centered and standardized due to the calculated parameters’ range varying noticeably. PCA is a projection method that is designed to model multivariate data with a relatively small number of so-called principal components. Principal components (PCs) are constructed as a linear combination of original variables in order to maximize the description of data variance; therefore, the number of relevant PCs was selected, taking into account the percentage of the modeled data variance. The first four PCs account for 86.4% of the total data variance, whereas the first two PCs describe approximately 65.2%, respectively. The score plot with the projection of 1,2,3-triazol-quinobenzothiazine derivatives **2a**–**n** on the plane PC1 vs. PC2 color-coded according to the experimental TLC lipophilicity is presented in Figure 3.

As a matter of fact, the analyzed 1,2,3-triazol-quinobenzothiazine derivatives (**2a**–**n**) are grouped in three main clusters according to the first principal component (PC1). Not surprisingly, the derivatives **2a**–**c**, containing an allyl substituent at the triazole ring, compose the first group with PC1 > 50, while molecules with the nitrobenzyl group are separated from the remaining ones (PC1 < −50). The rest of the benzene-based analogs are clustered together along −20 < PC1 < 20 with higher values of the empirical TLC lipophilic values, as shown in Figure 3. Interestingly, the structural dissimilarity between the investigated molecules is also observed along the second principal component (PC2), where all *ortho*-substituted derivatives **2c**,**f**,**i**,**l** are grouped together with PC2 < −20 and separated from *meta/para*-based analogs (PC2 > −10), respectively. 

**Figure 4:**
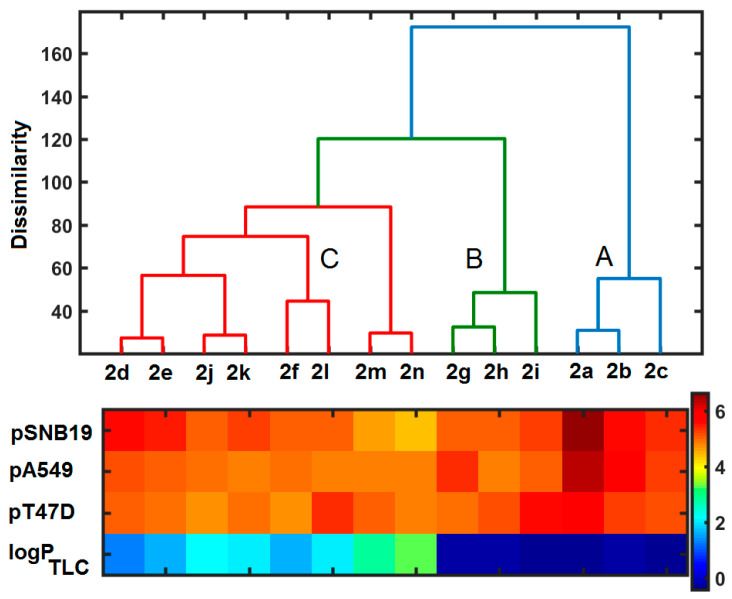
Dendrogram of 1,2,3-triazol-quinobenzothiazine derivatives **2a**–**n** in descriptor-based space with a color-coded map of experimental activities and lipophilicity values on a logarithmic scale. A,B,C—cluster names.

Overall, the exploratory HCA procedure generates the suboptimal clustering pattern of objects/molecules that is mainly dependent on the clusters’ linkage method employed [48]. Unfortunately, in-depth interpretability of the multidimensional data is impeded in the original descriptor space; therefore, the mutual (dis)similarities are illustrated as a 2D dendrogram produced in the Euclidean-based distance with the Ward linkage algorithm—the OX axis presents the order of objects or parameters, whereas the OY one shows the (dis)similarity between them. The dendrogram presented in Figure 4 confirms our previous PCA findings (see Figure 3), where derivatives **2a**–**c** with an allyl substituent at the triazole ring form cluster A that differs from collection B with the ortho/meta/para nitrobenzyl-based isomers **2g**,**h**,**i**. Likewise, the remaining benzene-containing analogs are grouped within cluster C, which is noticeably characterized by higher values of the experimental TLC lipophilic values, as shown in the color-coded map in Figure 4. Unfortunately, there are no evident SAR (structure-activity) relationships; however, molecules within clusters A and B are generally marked by slightly higher values of the cytotoxic activities against human cancer cell lines (A549, SNB-19, and T-47D) compared to the remaining ones (cluster C).

### 2.6. ClogP Estimation versus Experimental Lipophilicity

**Figure 5:**
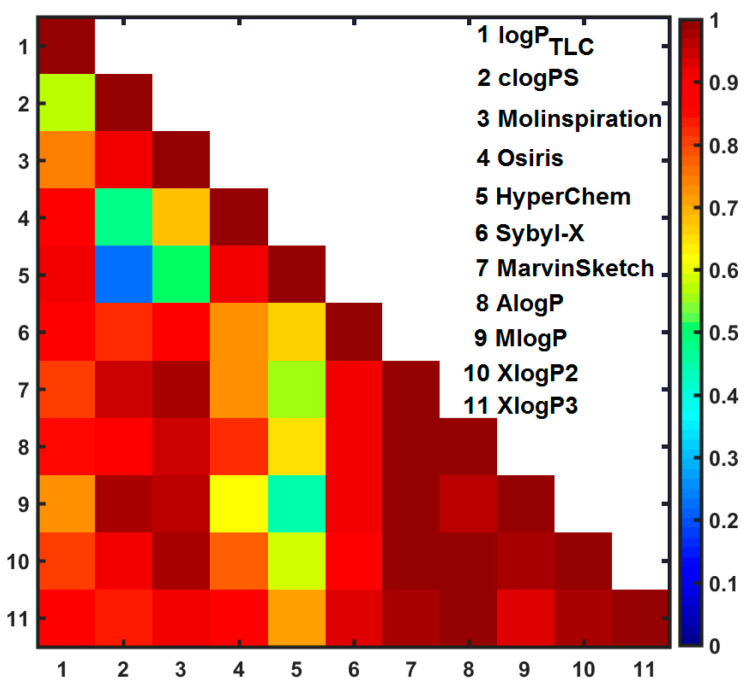
Matrix of correlation coefficients of linear relationships between experimental lipophilicity and calculated lipophilicity (clogP) for 1,2,3-triazol-quinobenzothiazine derivatives **2a**–**n**.

Understanding lipophilicity and its modulation has been recognized as a crucial factor in the description of both the pharmacokinetic (ADMET) and pharmacodynamic aspects of drug-receptor/enzyme interactions; therefore, the accurate and efficient measurement of experimental logP values is an important requirement in drug design. Alternatively, using empirical methods, some lipophilicity descriptors (clogP) have been proposed using mainly in silico predictive models. It is possible that some methods for theoretical calculation of lipophilicity might be more or less suitable for specific/heterogeneous series of compounds analyzed; thus, a variety of approaches should be employed in a *consensus* methodology and subsequently compared with the existing empirical data. Hence, the numerical approximation of clogP values for the examined series of 1,2,3-triazol-quinobenzothiazine derivatives **2a**–**n** was performed via a set of in silico logP estimators, including AlogPS, Molinspirations, Osiris, HyperChem 7.0, Sybyl-X, MarvinSketch 15, Dragon6.0, XlogP3, respectively. In order to compare the estimated clogP with the empirical lipophilic values, the thin-layer chromatography method (TLC) was used. In practice, the theoretically approximated partition coefficients (clogP) were (inter-)correlated with each other and cross-compared with the empirically specified logP_TLC_ parameters, as shown in Figure 5.

A relatively good correlation (ranging from r = 0.57 to r = 0.91) was observed between the generated clogP and the experimental lipophilic logP_TLC_ values for all used clogP predictors, with r > 0.90 recorded for the HyperChem and Sybyl-X programs. The averaged clogP values over the set of programs produced a value of r = 0.91, whereas the median resulted in r = 0.84 with the empirical data. Moreover, the iterative variable elimination procedure (IVE-PLS) was applied on the integrated clogP matrix (X_14×10_) and logP_TLC_ parameter, indicating clogPS, Sybyl-X, AlogP, and XlogP2 estimators as valid contributors to the linear QSPR model in namely *consensus* lipophilicity determination [49]. In consequence, the averaged values of the indicated clogP predictors were correlated with logP_TLC_ values, resulting in r = 0.84, because not only the best intercorrelated logP estimators were chosen. It should be emphasized that some variations in the clogP specification resulted probably from different computational algorithms (atom/fragment- or descriptor-based) implemented in the software and/or the training data used at the training step; therefore, the *consensus* approach seems advisable. 

## 3. Materials and Methods

### 3.1. Chemistry

Melting points are uncorrected. NMR spectra were recorded using a Bruker Ascend 600 spectrometer (Bruker, Billerica, MA, USA). The following 2D experiments were employed to assign the structures: ^1^H-^13^C gradient selected HSQC and HMBC sequences. Standard experimental conditions and standard Bruker programs were used. The ^1^H-NMR and ^13^C-NMR spectral data are provided relative to the TMS signal at 0.0 ppm. HR mass spectra were recorded with the Bruker Impact II (Bruker, Billerica, MA, USA).

#### 3.1.1. Synthesis of Propargiloxy-5-Methylo-12*H*-quino[3,4-*b*][1,4]benzothiazine Derivatives 2**a**–**c**

A total of 1 mmol of the corresponding propargyloxy-5-methyl-12*H*-quino[3,4-*b*][1,4]benzothiazinium chloride **1a**–**c** was dissolved in 50 mL of water at 50 °C. The resulting solution was filtered from solid residues. Then, 10 mL of 5% NaHCO_3_ aqueous solution was added dropwise to the solution while stirring. The resulting precipitate was suction-filtered and washed with water (3 × 10 mL). The obtained products were dried in a desiccator over anhydrous calcium chloride.

1. 9-propargyloxy-5-methyl-5H-quino [3,4-b][1,4]benzothiazine (**3a**): Yield: 90%; ^1^H NMR (DMSO_d-6_, 600 MHz), δ (ppm): 3.45 (s, 3H, CH_3_), 3.57–3.59 (m, 1H, CH), 4.68–4.75 (m, 2H, CH_2_), 6.35–6.40 (m, 1H, H_arom_), 6.48–6.53 (m, 1H, H_arom_), 6.70–6.76 (m, 1H, H_arom_), 6.97 (s, 1H, H6), 7.16–7.24 (m, 2H, H_arom_), 7.45–7.52 (m, 1H, H_arom_), 8.09–8.13 (m, 1H, H_arom_); ^13^C NMR (DMSO_d-6,_ 150.9 MHz), δ (ppm): 40.52 (CH_3_), 56.06 (CH_2_), 78.77, 79.68, 102.11, 112.33, 113.70, 115.67, 121.52, 121.92, 123.78, 124.79, 127.62, 131.74, 132.64, 139.01, 140.84, 151.73, 155.44; ESI-HRMS Calcd for C_19_H_15_N_2_OS ([M + H]^+^): 319.0905, found: 319.0901.

2. 10-propargyloxy-5-methyl-5H-quino [3,4-b][1,4]benzothiazine (**3b**): Yield: 95%; ^1^H NMR (DMSO_d-6_, 600 MHz), δ (ppm): 3.51 (s, 3H, CH_3_), 3.55–3.59 (m, 1H, CH), 4.68–4.72 (m, 2H, CH_2_), 6.41–6.45 (m, 2H, H_arom_), 6.56–6.61 (m, 1H, H_arom_), 7.07 (s, 1H, H6), 7.21–7.27 (m, 1H, H_arom_), 7.28–7.31 (m, 1H, H_arom_), 7.51–7.56 (m, 1H, H_arom_), 8.16–8.21 (m, 1H, H_arom_); ^13^C NMR (DMSO_d-6,_ 150.9 MHz), δ (ppm): 40.43 (CH_3_), 55.79 (CH_2_), 78.68, 79.85, 103.49, 112.09, 112.40, 113.30, 115.92, 121.25, 124.03, 125.02, 125.99, 132.05, 133.22, 140.74, 146.35, 154.27, 157.38; ESI-HRMS Calcd for C_19_H_15_N_2_OS ([M + H]^+^): 319.0905, found: 319.0906.

3. 11-propargyloxy-5-methyl-5H-quino [3,4-b][1,4]benzothiazine (**3c**): Yield: 88%; ^1^H NMR (DMSO_d-6_, 600 MHz), δ (ppm): 3.47 (s, 3H, CH_3_), 3.51–3.55 (m, 1H, CH), 4.81 (m, 2H, CH_2_), 6.31–6.36 (m, 1H, H_arom_), 6.60–6.67 (m, 1H, H_arom_), 6.68–6.75 (m, 1H, H_arom_), 6.98 (s, 1H, H6), 7.18–7.27 (m, 2H, H_arom_), 7.47–7.55 (m, 1H, H_arom_), 8.11–8.19 (m, 1H, H_arom_); ^13^CNMR (DMSO_d-6,_ 150.9 MHz), δ (ppm): 40.38 (CH_3_), 57.95 (CH_2_), 78.44, 80.41, 103.02, 115.82, 116.97, 119.94, 121.78, 121.84, 124.01, 125.13, 125.62, 131.96, 133.02, 136.12, 140.86, 151.75, 152.94; ESI-HRMS Calcd for C_19_H_15_N_2_OS ([M + H]^+^): 319.0905, found: 319.0911.

#### 3.1.2. Synthesis of Aniline Derivatives of 1,2,3-triazole **5a**–**n**

##### Procedure A: Preparation of Derivatives **5a**–**c**

Two 5 mL of anhydrous DMF, 1.5 mmol of allyl bromide, and 1.5 mmol (98 mg) of sodium azide were added, and the resulting suspension was stirred on a magnetic stirrer at room temperature for 24 h. Then, 1 mmol (147 mg) of the appropriate propargyloxyaniline **4a**–**c** was added. A solution of 40 mg of sodium ascorbate in 1 mL of distilled water and a solution of 25 mg of copper (II) sulfate pentahydrate in 1 mL of distilled water were prepared. The resulting aqueous solutions were mixed and added to the reaction mixture. The whole thing was stirred at room temperature for 24 h. The reaction mixture was then poured into 50 mL of water and extracted with chloroform (4 × 20 mL). The combined extracts were dried over anhydrous sodium sulfate. After evaporation of the solvent in a vacuum evaporator, the crude products obtained were purified by silica column chromatography. Chloroform was used as an eluent, followed by chloroform:ethanol *v/v* 10:1. 

##### Procedure B: Preparation of Derivatives **5d**–**l**

To 10 mL of anhydrous DMF was added 1.5 mmol of the appropriate azide: benzyl azide (3 mL 0.5 M solution in dichloromethane) or (phenylthio)methyl azide 248 mg and 1 mmol (147 mg) of the appropriate propargyloxy aniline **4a**–**c**. A solution of 40 mg of sodium ascorbate in 1 mL of distilled water and a solution of 25 mg of copper (II) sulfate (VI) pentahydrate in 1 mL of distilled water were prepared. The resulting aqueous solutions were mixed and added to the reaction mixture. The whole thing was stirred at room temperature for 24 h. The reaction mixture was then poured into 50 mL of water and extracted with chloroform (4 × 20 mL). The combined extracts were dried over anhydrous sodium sulfate. After evaporation of the solvent in a vacuum evaporator, the crude products obtained were purified by silica column chromatography. Chloroform was used as an eluent, followed by chloroform:ethanol *v/v* 10:1. 

##### Procedure C: Preparation of Derivatives **5m**–**n**

Then, 10 mL of anhydrous DMF, 1.5 mmol of 4-chlorophenyl azide (3 mL of a 0.5 M solution in tert-butyl methyl ether), and 1 mmol (147 mg) of the appropriate propargyloxyaniline **4b** or **4c** were placed in a microwave reactor tube. A solution of 40 mg of sodium ascorbate in 1 mL of distilled water and a solution of 25 mg of copper (II) sulfate (VI) pentahydrate in 1 mL of distilled water were prepared. The resulting aqueous solutions were mixed and added to the reaction mixture. The reaction was carried out in a microwave reactor at 100 °C for 45 min. The solution was then cooled to room temperature and poured into 50 mL of water. The whole was extracted with chloroform (4 × 20 mL). The combined extracts were dried over anhydrous sodium sulfate. After evaporation of the solvent in vacuo, the crude products were purified by silica column chromatography. Chloroform was used as an eluent, followed by chloroform:ethanol *v/v* 10:1.

1. 1-allyl-4-(2-aminophenoxy)methyl-1H-1,2,3-triazole (**5a**): Procedure A. Yield: 67%; ^1^H NMR (CDCl_3_, 600 MHz), δ (ppm): 3.83 (s, 2H, NH_2_), 4.91–4.98 (m, 2H, NCH_2_), 5.16 (s, 2H, OCH_2_), 5.22–5.28 (m, 1H, CH=CH_2_), 5.30–5.35 (m, 1H, CH=CH_2_), 5.95–6.03 (m, 1H, CH=CH_2_), 6.65–6.72 (m, 2H, H_arom_), 6.76–6.81 (m, 1H, H_arom_), 6.89–6.92 (m, 1H, H_arom_), 7.60 (s, 1H, CH); ^13^C NMR (CDCl_3_, 150.9 MHz), δ (ppm): 52.72, 62.42, 112.44, 115.32, 118.31, 120.25, 121.89, 122.71, 131.14, 136.71, 144.38, 145.84; ESI-HRMS Calcd for C_12_H_15_N_4_O ([M + H]^+^): 231.1246, found: 231.1235.

2. 1-allyl-4-(3-aminophenoxy)methyl-1H-1,2,3-triazole (**5b**): Procedure A. Yield: 72%; ^1^H NMR (CDCl_3_, 600 MHz), δ (ppm): 4.11 (s, 2H, NH_2_), 4.96–5.01 (m, 2H, NCH_2_), 5.16 (s, 2H, OCH_2_), 5.28–5.32 (m, 1H, CH=CH_2_), 5.33–5.39 (m, 1H, CH=CH_2_), 5.95–6.05 (m, 1H, CH=CH_2_), 6.32–6.45 (m, 3H, H_arom_), 7.02–7.10 (m, 1H, H_arom_), 7.62 (s, 1H, CH); ^13^C NMR (CDCl_3_, 150.9 MHz), δ (ppm): 51.76, 60.87, 101.09, 104.19, 107.71, 119.32, 121.52, 129.09, 129.17, 130.08, 143.55, 146.12; ESI-HRMS Calcd for C_12_H_15_N_4_O ([M + H]^+^): 231.1246, found: 231.1252.

3. 1-allyl-4-(4-aminophenoxy)methyl-1H-1,2,3-triazole (**5c**): Procedure A. Yield: 75%; ^1^H NMR (CDCl_3_, 600 MHz), δ (ppm): 3.57 (s, 2H, NH_2_), 4.82–4.88 (m, 2H, NCH_2_), 5.00 (s, 2H, OCH_2_), 5.14–5.18 (m, 1H, CH=CH_2_), 5.20–5.22 (m, 1H, CH=CH_2_), 5.85–5.95 (m, 1H, CH=CH_2_), 6.50–6.55 (m, 2H, H_arom_), 6.68–6.72 (m, 2H, H_arom_), 7.54 (s, 1H, CH); ^13^C NMR (CDCl_3_, 150.9 MHz), δ (ppm): 52.59, 62.61, 115.92, 116.17, 120.04, 122.76, 131.20, 140.56, 144.56, 151.03; ESI-HRMS Calcd for C_12_H_15_N_4_O ([M + H]^+^): 231.1246, found: 231.1250.

4. 1-benzyl-4-(2-aminophenoxy)methyl-1H-1,2,3-triazole (**5d**): Procedure B. Yield: 59%; ^1^H NMR (CDCl_3_, 600 MHz), δ (ppm): 3.73 (s, 2H, NH_2_), 5.19 (s, 2H, CH_2_), 5.52 (s, 2H, CH_2_), 6.70–6.75 (m, 2H, H_arom_), 6.77–6.83 (m, 1H, H_arom_), 6.90–6.92 (m, 1H, H_arom_), 7.25–7.30 (m, 2H, H_arom_), 7.33–7.40 (m, 3H, H_arom_), 7.55 (s, 1H, CH); ^13^C NMR (CDCl_3_, 150.9 MHz), δ (ppm): 54.21 (CH_2_), 62.54 (CH_2_), 112.64, 115.45, 118.49, 121.96, 122.75, 128.09, 128.81, 129.15, 134.52, 136.58, 144.62, 145.91; ESI-HRMS Calcd for C_16_H_17_N_4_O ([M + H]^+^): 281.1402, found: 281.1403.

5. 1-benzyl-4-(3-aminophenoxy)methyl-1H-1,2,3-triazole (**5e**): Procedure B. Yield: 74%; ^1^H NMR (CDCl_3_, 600 MHz), δ (ppm): 3.99 (s, 2H, NH_2_), 5.12 (s, 2H, CH_2_), 5.51 (s, 2H, CH_2_), 6.28–6.35 (m, 2H, H_arom_), 6.35–6.39 (m, 1H, H_arom_), 7.01–7.08 (m, 1H, H_arom_), 7.21–7.31 (m, 2H, H_arom_), 7.31–7.39 (m, 3H, H_arom_), 7.54 (s, 1H, CH); ^13^C NMR (CDCl_3_, 150.9 MHz), δ (ppm): 54.20 (CH_2_), 61.91 (CH_2_), 102.07, 105.11, 108.68, 122.67, 128.12, 128.77, 129.12, 130.16, 134.51, 144.75, 147.29, 159.34; ESI-HRMS Calcd for C_16_H_17_N_4_O ([M + H]^+^): 281.1402, found: 281.1400.

6. 1-benzyl-4-(4-aminophenoxy)methyl-1H-1,2,3-triazole (**5f**): Procedure B. Yield: 64%; ^1^H NMR (CDCl_3_, 600 MHz), δ (ppm): 3.29 (s, 2H, NH_2_), 5.03 (s, 2H, CH_2_), 5.45 (s, 2H, CH_2_), 6.52–6.61 (m, 2H, H_arom_), 6.68–6.77 (m, 2H, H_arom_), 7.15–7.21 (m, 2H, H_arom_), 7.22–7.32 (m, 3H, H_arom_), 7.43 (s, 1H, CH); ^13^C NMR (CDCl_3_, 150.9 MHz), δ (ppm): 53.18 (CH_2_), 61.86 (CH_2_), 115.03, 115.45, 121.48, 127.09, 127.76, 128.11, 133.47, 139.17, 144.02, 150.42; ESI-HRMS Calcd for C_17_H_16_N_4_O ([M + H]^+^): 281.1402, found: 281.1438.

7. 1-(4-nitrobenzyl)-4-(2-aminophenoxy)methyl-1H-1,2,3-triazole (**5g**): Procedure A. Yield: 72%; ^1^H NMR (CDCl_3_, 600 MHz), δ (ppm): 5.26 (s, 2H, CH_2_), 5.66 (s, 2H, CH_2_), 6.69–6.76 (m, 2H, H_arom_), 6.82–6.86 (m, 1H, H_arom_), 6.90–6.93 (m, 1H, H_arom_), 7.38–7.43 (m, 2H, H_arom_), 7.61 (s, 1H, CH), 8.21–8.28 (m, 2H, H_arom_); ^13^C NMR (CDCl_3_, 150.9 MHz), δ (ppm): 53.16 (CH_2_), 62.43 (CH_2_), 112.65, 115.50, 118.52, 122.13, 122.97, 124.35, 128.59, 136.63, 141.54, 145.25, 145.68, 148.08; ESI-HRMS Calcd for C_16_H_16_N_5_O_3_ ([M + H]^+^): 326.1253, found: 326.1249.

8. 1-(4-nitrobenzyl)-4-(3-aminophenoxy)methyl-1H-1,2,3-triazole (**5h**): Procedure A. Yield: 59%; ^1^H NMR (CDCl_3_, 600 MHz), δ (ppm): 5.18 (s, 2H, CH_2_), 5.65 (s, 2H, CH_2_), 6.28–6.35 (m, 2H, H_arom_), 6.35–6.39 (m, 1H, H_arom_), 7.02–7.09 (m, 1H, H_arom_), 7.38–7.43 (m, 2H, H_arom_), 7.50–7.56 (m, 1H, H_arom_), 8.02 (s, 1H, CH), 8.18–8.25 (m, 3H, H_arom_); ^13^C NMR (CDCl_3_, 150.9 MHz), δ (ppm): 53.15 (CH_2_), 61.85 (CH_2_), 101.76, 104.67, 108.51, 122.89, 124.33, 128.52, 128.62, 130.24, 141.58, 145.45, 147.91, 159.22; ESI-HRMS Calcd for C_16_H_16_N_5_O_3_ ([M + H]^+^): 326.1253, found: 326.1266.

9. 1-(4-nitrobenzyl)-4-(4-aminophenoxy)methyl-1H-1,2,3-triazole (**5i**): Procedure A. Yield: 52%; ^1^H NMR (DMSO_d-6_, 600 MHz), δ (ppm): 4.66 (s, 2H, NH_2_), 4.99 (s, 2H, CH_2_), 5.80 (s, 2H, CH_2_), 6.49–6.52 (d, ^3^*J* = 8,4 Hz, 2H, H_arom_), 6.68–6.73 (d, ^3^*J*= 8,4 Hz, 2H, H_arom_), 7.48–7.53 (m, 2H, H_arom_), 8.21–8.28 (m, 2H, H_arom_), 8.29 (s, 1H, CH); ^13^C NMR (DMSO_d-6_, 150.9 MHz), δ (ppm): 56.49 (CH_2_), 62.18 (CH_2_), 115.27, 116.24, 124.41, 125.33, 129.45, 143.30, 143.97, 144.27, 147.69, 149.68; ESI-HRMS Calcd for C_16_H_16_N_5_O_3_ ([M + H]^+^): 326.1253, found: 326.1242.

10**.** 1-(phenylthio)methyl-4-(2-aminophenoxy)methyl-1H-1,2,3-triazole (**5j**): Procedure B. Yield: 70%; ^1^H NMR (CDCl_3_, 600 MHz), δ (ppm): 5.17 (s, 2H, CH_2_), 5.59 (s, 2H, CH_2_), 6.60–6.72 (m, 2H, H_arom_), 6.78–6.85 (m, 1H, H_arom_), 6.86–6.92 (m, 1H, H_arom_), 7.23–7.31 (m, 5H, H_arom_), 7.60 (s, 1H, CH); ^13^C NMR (CDCl_3_, 150.9 MHz), δ (ppm): 53.96 (CH_2_), 62.37 (CH_2_), 112.42, 115.60, 118.51, 121.85, 122.50, 128.77, 129.51, 131.72, 132.39, 136.42, 144.91, 145.94; ESI-HRMS Calcd for C_16_H_17_N_4_OS ([M + H]^+^): 313.1123, found: 313.1139.

11**.** 1-(phenylthio)methyl-4-(3-aminophenoxy)methyl-1H-1,2,3-triazole (**5k**): Procedure B. Yield: 68%; ^1^H NMR (CDCl_3_, 600 MHz), δ (ppm): 3.84 (s, 2H, NH_2_), 5.14 (s, 2H, CH_2_), 5.60 (s, 2H, CH_2_), 6.28–6.34 (m, 2H, H_arom_), 6.35–6.40 (m, 1H, H_arom_), 7.03–7.08 (m, 1H, H_arom_), 7.26–7.32 (m, 5H, H_arom_), 7.60 (s, 1H, CH), ^13^C NMR (CDCl_3_, 150.9 MHz), δ (ppm): 53.99 (CH_2_), 61.80 (CH_2_), 101.96, 104.93, 108.57, 122.27, 128.78, 129.53, 130.19, 131.73, 132.39, 145.01, 147.60, 159.24; ESI-HRMS Calcd for C_16_H_17_N_4_OS ([M + H]^+^): 313.1123, found: 313.1122.

12**.** 1-(phenylthio)methyl-4-(3-aminophenoxy)methyl-1H-1,2,3-triazole (**5l**): Procedure B. Yield: 75%; ^1^H NMR (CDCl_3_, 600 MHz), δ (ppm): 5.12 (s, 2H, CH_2_), 5.62 (s, 2H, CH_2_), 6.61–6.68 (m, 2H, H_arom_), 6.78–6.82 (m, 2H, H_arom_), 7.28–7.35 (m, 5H, H_arom_), 7.61 (s, 1H, CH); ^13^C NMR (CDCl_3_, 150.9 MHz), δ (ppm): 53.98 (CH_2_), 62.73 (CH_2_), 116.08, 116.36, 122.16, 128.78, 129.53, 131.75, 132.36, 140.55, 145.30, 151.21; ESI-HRMS Calcd for C_16_H_17_N_4_OS ([M + H]^+^): 313.1123, found: 313.1114.

13**.** 1-(4-chlorophenyl)-4-(3-aminophenoxy)methyl-1H-1,2,3-triazole (**5m**): Procedure C. Yield: 58%; ^1^H NMR (DMSO_d-6_, 600 MHz), δ (ppm): 5.09 (s, 2H, NH_2_), 5.11 (s, 2H, CH_2_), 6.15–6.25 (m, 3H, H_arom_), 6.89–6.95 (m, 1H, H_arom_), 7.67–7.72 (m, 2H, H_arom_), 7.95–8.02 (m, 2H, H_arom_), 8.96 (s, 1H, CH); ^13^C NMR (DMSO_d-6_, 150.9 MHz), δ (ppm): 61.03 (CH_2_), 100.68, 102.45, 107.79, 122.29, 123.25, 130.09, 130.36, 133.47, 135.85, 144.88, 150.52, 159.46; ESI-HRMS Calcd for C_15_H_14_ClN_4_O ([M + H]^+^): 301.0856, found: 301.0857.

14**.** 1-(4-chlorophenyl)-4-(4-aminophenoxy)methyl-1H-1,2,3-triazole (**5n**): Procedure C. Yield: 67%; ^1^H NMR (CDCl_3_, 600 MHz), δ (ppm): 3.50 (s, 2H, NH_2_), 5.24 (s, 2H, CH_2_), 6.63–6.72 (d, ^2^*J* = 8,4 Hz, 2H, H_arom_), 6.83–6.91 (d, ^2^*J* = 8,4 Hz, 2H, H_arom_), 7.50–7.55 (d, ^2^*J*= 9 Hz, 2H, H_arom_), 7.69–7.72 (d, ^2^*J* = 9 Hz, 2H, H_arom_), 8.03 (s, 1H, CH); ^13^C NMR (DMSO_d-6_, 150.9 MHz), δ (ppm): 62.74 (CH_2_), 116.03, 116.39, 120.69, 121.75, 129.97, 134.63, 135.49, 140.69, 145.76, 151.22; ESI-HRMS Calcd for C_15_H_14_ClN_4_O ([M + H]^+^): 301.0856, found: 301.0842.

#### 3.1.3. Synthesis of 1,2,3-Triazole Derivatives of 5-Methyl-12*H*-quino[3,4-*b*][1,4]benzothiazinium Chlorides **2a**–**n**

##### Procedure A

A 2.5 mmol of the appropriate aniline 1,2,3-triazole derivative **5** was added to a suspension of 1 mmol (419 mg) of 5,12-(dimethyl)thioquinanthrene diinium *bis*-chloride **6** in 5 mL of anhydrous pyridine. The whole thing was heated at 80 °C for 24 h with intensive stirring. The mixture was cooled to room temperature. The resulting precipitate was suction-filtered and washed with anhydrous ether (3 × 15 mL). The crude products were purified by alumina column chromatography using chloroform:ethanol *v/v* 10:1 as an eluent. 

##### Procedure B

A 2.5 mmol of the appropriate aniline 1,2,3-triazole derivative **5** was added to a suspension of 1 mmol (419 mg) of 5,12-(dimethyl)thioquinanthrenediinium *bis*-chloride **6** in 5 mL of anhydrous pyridine. The reaction mixture was stirred vigorously for 7 days at room temperature. The resulting precipitate was filtered off under reduced pressure and washed with anhydrous ether (3 × 15mL). The crude products were purified by alumina column chromatography using chloroform:ethanol *v/v* 10:1 as an eluent.

1. 5-Methyl-9-(1-allyl-1H-1,2,3-triazol-4-yl)methoxy-12H-quino [3,4-b][1,4]benzothiazinium chloride (**2a**): Procedure A. Yield: 68%; ^1^H NMR (DMSO_d-6_, 600 MHz), δ (ppm): 4.09 (s, 3H, CH_3_), 5.00–5.09 (d, *J* = 5,4 Hz, 2H, CH_2_-CH), 5.10 (s, 2H, OCH_2_), 5.16–5.21 (m, 1H, CH=CH_2_), 5.22–5.32 (m, 1H, CH=CH_2_), 6.00–6.11 (m, 1H, CH=CH_2_), 6.70–6.82 (m, 2H, H_arom_), 7.55–7.62 (m, 1H, H_arom_), 7.70–7.78 (m, 1H, H_arom_), 7.92–8.03 (m, 2H, H_arom_), 8.24 (s, 1H, CH), 8.52–8.61 (m, 1H, H_arom_), 9.05–9.11 (m, 1H, H_arom_), 11.26 (s, 1H, NH); ^13^C NMR (DMSO_d-6_, 150.9 MHz), δ (ppm): 42.81 (CH_3_), 52.17 (CH-CH_2_), 61.85 (OCH_2_), 105.00, 113.43, 114.42, 115.82, 118.54, 118.91 (CH=CH_2_), 120.37, 124.57, 124.79, 125.21 (C=CH), 127.85, 129.78, 133.22 (CH=CH_2_), 134.70, 139.07, 142.81, 142.97 (C6), 151.06, 157.31; ESI-HRMS Calcd for C_22_H_20_N_5_OS ([M]^+^): 402.1389, found: 402.1390.

2. 5-Methyl-10-(1-allyl-1H-1,2,3-triazol-4-yl)methoxy-12H-quino [3,4-b][1,4]benzothiazinium chloride (**2b**): Procedure B. Yield: 42%; ^1^H NMR (CD_3_OD, 600 MHz), δ (ppm): 4.21 (s, 3H, CH_3_), 5.04–5.11 (d, *J* = 6,0 Hz, 2H, CH_2_-CH), 5.22–5.30 (m, 1H, CH=CH_2_), 5.31–5.38 (m, 1H, CH=CH_2_), 5.39 (s, 2H, OCH_2_), 6.05–6.12 (m, 1H, CH=CH_2_), 6.61–6.68 (m, 1H, H_arom_), 7.04–7.11 (m, 2H, H_arom_), 7.79–7.85 (m, 1H, H_arom_), 8.02–8.11 (m, 2H, H_arom_), 8.16 (s, 1H, C=CH), 8.31–8.34 (m, 1H, H_arom_), 8.45 (s, 1H, H6); ^13^C NMR (CD_3_OD, 150.9 MHz), δ (ppm): 42.26 (CH_3_), 52.28 (CH-CH_2_), 62.55 (OCH_2_), 108.08, 113.56, 115.79, 118.22, 118.48, 118.56, 118.67, 119.45, 122.46, 124.15, 125.65, 127.47, 128.18, 131.73, 134.62, 139.13, 142.78, 147.03, 151.74; ESI-HRMS Calcd for C_22_H_20_N_5_OS ([M]^+^): 402.1389, found: 402.1392.

3. 5-Methyl-11-(1-allyl-1H-1,2,3-triazol-4-yl)methoxy-12H-quino [3,4-b][1,4]benzothiazinium chloride (**2c**): Procedure A. Yield: 72%; ^1^H NMR (DMSO_d-6_, 600 MHz), δ (ppm): 4.18 (s, 3H, CH_3_), 5.02–5.08 (d, *J* = 4,8 Hz, 2H, CH_2_-CH), 5.10–5.18 (m, 1H, CH=CH_2_), 5.22–5.30 (m, 1H, CH=CH_2_), 5.37 (s, 2H, OCH_2_), 6.01–6.09 (m, 1H, CH=CH_2_), 6.74–6.83 (m, 1H, H_arom_), 7.06–7.13 (m, 1H, H_arom_), 7.18–7.22 (m, 1H, H_arom_), 7.80–7.88 (m, 1H, H_arom_), 8.05–8.16 (m, 2H, H_arom_), 8.32 (s, 1H, C=CH), 8.38–8.45 (m, 1H, H_arom_), 8.81 (s, 1H, H6), 9.89 (s, 1H, NH); ^13^C NMR (DMSO_d-6_, 150.9 MHz), δ (ppm): 43.35 (CH_3_), 52.16 (CH-CH_2_), 63.83 (OCH_2_), 107.71, 114.99, 116.25, 118.99, 119.08 (CH=CH_2_), 119.34, 120.23, 123.71, 125.15 (C=CH), 126.32, 127.99, 128.46, 133.26 (CH=CH_2_), 135.04, 139.12, 143.02(C6), 144.25, 147.71, 151.89; ESI-HRMS Calcd for C_22_H_20_N_5_OS ([M]^+^): 402.1389, found: 402.1370.

4. 5-Methyl-9-(1-benzyl-1H-1,2,3-triazol-4-yl)methoxy-12H-quino [3,4-b][1,4]benzothiazinium chloride (**2d**): Procedure A. Yield: 63%; ^1^H NMR (DMSO_d-6_, 600 MHz), δ (ppm): 4.09 (s, 3H, CH_3_), 5.11 (s, 2H, CH_2_), 5.63 (s, 2H, CH_2_), 6.78–6.84 (m, 2H, H_arom_), 7.28–7.50 (m, 6H, H_arom_), 7.75–7.83 (m, 1H, H_arom_), 7.95–8.07 (m, 2H, H_arom_), 8.31 (s, 1H, C=CH), 8.53–8.62 (m, 1H, H_arom_), 8.91 (s, 1H, H6), 11.03 (s, 1H, NH); ^13^C NMR (DMSO_d-6_, 150.9 MHz), δ (ppm): 42.85 (CH_3_), 53.31 (CH_2_), 61.90 (OCH_2_), 105.12, 113.59, 114.60, 115.84, 118.55, 119.04, 120.25, 124.43, 125.33, 127.98, 128.44, 128.66, 129.26, 134.77, 136.47, 139.12, 143.00, 143.08, 148.87, 151.11, 157.37; ESI-HRMS Calcd for C_26_H_22_N_5_OS ([M]^+^): 452.1545, found: 452.1547.

5. 5-Methyl-10-(1-benzyl-1H-1,2,3-triazol-4-yl)methoxy-12H-quino [3,4-b][1,4]benzothiazinium chloride (**2e**): Procedure B. Yield: 52%; ^1^H NMR (DMSO_d-6_, 600 MHz), δ (ppm): 4.15 (s, 3H, CH_3_), 5.12 (s, 2H, CH_2_), 5.63 (s, 2H, CH_2_), 6.78–6.83 (m, 1H, H_arom_), 6.98–7.05 (m, 1H, H_arom_), 7.21–7.41 (m, 6H, H_arom_), 7.81–7.89 (m, 1H, H_arom_), 8.05–8.12 (m, 2H, H_arom_), 8.36 (s, 1H, C=CH), 8.69 (s, 1H, H6), 8.85–9.05 (m, 1H, H_arom_), 10.95 (s, 1H, NH); ^13^C NMR (CD_3_OD, 150.9 MHz), δ (ppm): 42.11 (CH_3_), 53.60 (CH_2_), 61.17 (OCH_2_), 105.70, 107.79, 108.15, 113.07, 115.76, 118.23, 122.53, 124.19, 127.31, 127.74, 128.09, 128.25, 128.65, 134.41, 135.31, 137.17, 139.08, 142.63, 143.33, 152.18, 158.93; ESI-HRMS Calcd for C_26_H_22_N_5_OS ([M]^+^): 452.1545, found: 452.1571. 

6. 5-Methyl-11-(1-benzyl-1H-1,2,3-triazol-4-yl)methoxy-12H-quino [3,4-b][1,4]benzothiazinium chloride (**2f**): Procedure A. Yield: 67%; ^1^H NMR (DMSO_d-6_, 600 MHz), δ (ppm): 4.17 (s, 3H, CH_3_), 5.35 (s, 2H, CH_2_), 5.63 (s, 2H, OCH_2_), 6.73–6.79 (m, 1H, H_arom_), 7.05–7.14 (m, 1H, H_arom_), 7.14–7.22 (m, 1H, H_arom_), 7.26–7.42 (m, 5H, H_arom_), 7.75–7.83 (m, 1H, H_arom_), 8.04–8.17 (m, 2H, H_arom_), 8.32–8.42 (m, 2H, H_arom_), 8.79 (s, 1H, H6), 9.86 (s, 1H, NH); ^13^C NMR (DMSO_d-6_, 150.9 MHz), δ (ppm): 43.33 (CH_3_), 53.30 (CH_2_), 63.87 (OCH_2_), 107.70, 115.09, 116.22, 118.97, 119.31, 120.25, 123.72, 125.32, 126.34, 127.97, 128.31, 128.41, 128.63, 129.23, 135.03, 136.48, 139.10, 143.17, 144.22, 147.66, 151.84; ESI-HRMS Calcd for C_26_H_22_N_5_OS ([M]^+^): 452.1545, found: 452.1524.

7. 5-Methyl-9-[1-(4-nitrobenzyl)-1H-1,2,3-triazol-4-yl]methoxy-12H-quino [3,4-b][1,4]benzothiazinium chloride (**2g**): Procedure A. Yield: 75%; ^1^H NMR (DMSO_d-6_, 600 MHz), δ (ppm): 4.09 (s, 3H, CH_3_), 5.14 (s, 2H, CH_2_), 5.83 (s, 2H, CH_2_), 6.78–6.89 (m, 2H, H_arom_), 7.45–7.51 (m, 1H, H_arom_), 7.51–7.54 (m, 2H, H_arom_), 7.73–7.81 (m, 1H, H_arom_), 7.99–8.08 (m, 2H, H_arom_), 8.22–8.31 (m, 2H, H_arom_), 8.39 (s, 1H, C=CH), 8.57 (s, 1H, H6), 8.91–8.99 (m, 1H, H_arom_), 11.08 (s, 1H, NH); ^13^C NMR (DMSO_d-6_, 150.9 MHz), δ (ppm): 42.85 (CH_3_), 52.39 (CH_2_), 61.87 (CH_2_), 105.1, 113.61, 114.65, 115.85, 118.56, 119.36, 120.28, 124.42, 124.48, 125.78, 127.98, 129.53, 129.81, 134.78, 139.12, 143.08, 143.17, 143.87, 147.71, 151.12, 157.31; ESI-HRMS Calcd for C_26_H_21_N_6_O_3_S ([M]^+^): 497.1396, found: 497.1395.

8. 5-Methyl-10-[1-(4-nitrobenzyl)-1H-1,2,3-triazol-4-yl]methoxy-12H-quino [3,4-b][1,4]benzothiazinium chloride (**2h**): Procedure B. Yield: 54%; ^1^H NMR (CD_3_OD, 600 MHz), δ (ppm): 4.21 (s, 3H, CH_3_), 5.20 (s, 2H, CH_2_), 5.81 (s, 2H, CH_2_), 6.87–6.92 (m, 1H, H_arom_), 6.92–6.95 (m, 1H, H_arom_), 7.49–7.54 (m, 2H, H_arom_), 7.82–7.87 (m, 1H, H_arom_), 8.05–8.11 (m, 2H, H_arom_), 8.20–8.28 (m, 4H, H_arom_), 8.42 (s, 1H, C=CH), 8.51–8.56 (m, 1H, H_arom_); ^13^C NMR (CD_3_OD, 150.9 MHz), δ (ppm): 42.13 (CH_3_), 52.51 (CH_2_), 61.16 (CH_2_), 105.80, 107.85, 108.12, 113.24, 115.74, 118.24, 122.52, 123.62, 124.72, 127.30, 128.11, 128.61, 134.43, 137.15, 139.08, 142.59, 142.64, 143.56, 147.90, 152.12, 158.80; ESI-HRMS Calcd for C_26_H_21_N_6_O_3_S ([M]^+^): 497.1396, found: 497.1398.

9. 5-Methyl-11-[1-(4-nitrobenzyl)-1H-1,2,3-triazol-4-yl]methoxy-12H-quino [3,4-b][1,4]benzothiazinium chloride (**2i**): Procedure A. Yield: 70%; ^1^H NMR (DMSO_d-6_, 600 MHz), δ (ppm): 4.17 (s, 3H, CH_3_), 5.38 (s, 2H, CH_2_), 5.84 (s, 2H, CH_2_), 6.72–6.84 (m, 1H, H_arom_), 7.07–7.15 (m, 1H, H_arom_), 7.15–7.22 (m, 1H, H_arom_), 7.45–7.55 (m, 2H, H_arom_), 7.72–7.82 (m, 1H, H_arom_), 8.02–8.17 (m, 2H, H_arom_), 8.17–8.28 (m, 2H, H_arom_), 8.35–8.42 (m, 1H, H_arom_), 8.47 (s, 1H, C=CH), 8.79 (s, 1H, H6), 9.85 (s, 1H, NH); ^13^C NMR (DMSO_d-6_, 150.9 MHz), δ (ppm): 43.35 (CH_3_), 52.40 (CH_2_), 63.77 (CH_2_), 107.71, 115.09, 116.23, 118.95, 119.33, 120.29, 123.70, 124.37, 125.76, 126.35, 127.99, 128.42, 129.41, 135.00, 139.11, 143.34, 143.93, 144.22, 147.63, 147.68, 151.84; ESI-HRMS Calcd for C_26_H_21_N_6_O_3_S ([M]^+^): 497.1396, found: 497.1395.

10. 5-Methyl-9-(1-(phenylthio)methyl-1H-1,2,3-triazol-4-yl)methoxy-12H-quino [3,4-b][1,4]benzothiazinium chloride (**2j**): Procedure A. Yield: 76%; ^1^H NMR (DMSO_d-6_, 600 MHz), δ (ppm): 4.03 (s, 3H, CH_3_), 4.93 (s, 2H, CH_2_), 5.61 (s, 2H, CH_2_), 5.91–5.94 (m, 1H, H_arom_), 6.40–6.48 (m, 1H, H_arom_), 7.18–7.28 (m, 5H, H_arom_), 7.29–7.35 (m, 1H, H_arom_), 7.35–7.43 (m, 1H, H_arom_), 7.43–7.50 (m, 1H, H_arom_), 7.70 (s, 1H, C=CH lub C6), 7.76 (s, 1H, H6 lub C=CH), 7.96–8.01 (m, 1H, H_arom_), 9.30–9.37 (m, 1H, H_arom_), 11.39 (s, 1H, NH); ^13^C NMR (DMSO_d-6_, 150.9 MHz), δ (ppm): 43.01 (CH_3_), 53.94 (SCH_2_), 61.84 (CH_2_), 105.33, 112.66, 113.49, 115.44, 116.62, 118.32, 121.38, 123.01, 126.54, 127.28, 128.76, 129.07, 129.60, 131.81, 132.05, 134.15, 138.51, 140.40, 143.71, 150.56, 157.00; ESI-HRMS Calcd for C_26_H_22_N_5_OS_2_ ([M]^+^): 484.1266, found: 484.1276.

11. 5-Methyl-10-(1-(phenylthio)methyl-1H-1,2,3-triazol-4-yl)methoxy-12H-quino [3,4-b][1,4] benzothiazinium chloride (**2k**): Procedure B. Yield: 45%; ^1^H NMR (DMSO_d-6_, 600 MHz), δ (ppm): 4.14 (s, 3H, CH_3_), 5.10 (s, 2H, SCH_2_), 5.98 (s, 2H, CH_2_), 6.71–6.80 (m, 1H, H_arom_), 6.92–7.01 (m, 1H, H_arom_), 7.22–7.45 (m, 6H, H_arom_), 7.80–7.85 (m, 1H, H_arom_), 7.98–8.12 (m, 2H, H_arom_), 8.28 (s, 1H, C=CH), 8.67 (s, 1H, C6), 8.98–9.08 (m, 1H, H_arom_), 11.10 (s, 1H, NH); ^13^C NMR (DMSO_d-6_, 150.9 MHz), δ (ppm): 43.13 (CH_3_), 52.18 (SCH_2_), 61.52 (CH_2_), 106.78, 107.03, 107.50, 113.39, 116.10, 119.20, 124.49, 127.93, 128.27, 129.75, 130.99, 132.82, 134.84, 137.13, 137.88, 139.05, 143.01, 143.68, 149.69, 151.70, 158.51; ESI-HRMS Calcd for C_26_H_22_N_5_OS_2_ ([M]^+^): 484.1266, found: 484.1278.

12. 5-Methyl-11-(1-(phenylthio)methyl-1H-1,2,3-triazol-4-yl)methoxy-12H-quino [3,4-b][1,4] benzothiazinium chloride (**2l**): Procedure A. Yield: 67%; ^1^H NMR (CD_3_OD, 600 MHz), δ (ppm): 4.18 (s, 3H, CH_3_), 5.34 (s, 2H, CH_2_), 5.82 (s, 2H, CH_2_), 6.56–6.62 (m, 1H, H_arom_), 7.01–7.06 (m, 2H, H_arom_), 7.22–7.28 (m, 3H, H_arom_), 7.29–7.33 (m, 2H, H_arom_), 7.72–7.78 (m, 1H, H_arom_), 8.00–8.08 (m, 2H, H_arom_), 8.16 (s, 1H, C=CH), 8.26–8.31 (m, 1H, H_arom_), 8.40 (s, 1H, H6); ^13^C NMR (DMSO_d-6_, 150.9 MHz), δ (ppm): 42.20 (CH_3_), 53.40 (CH_2_), 62.47 (CH_2_), 108.05, 113.93, 115.82, 118.19, 118.50, 122.49, 123.79, 125.91, 127.10, 127.47, 128.21, 129.03, 131.97, 134.60, 139.14, 142.30, 142.80, 143.10, 145.96, 146.83, 151.81; ESI-HRMS Calcd for C_26_H_22_N_5_OS_2_ ([M]^+^): 484.1266, found: 484.1273.

13. 5-Methyl-9-(1-(4-chlorophenyl)-1H-1,2,3-triazol-4-yl)methoxy-12H-quino [3,4-b][1,4]benzothiazinium chloride (**2m**): Procedure A. Yield: 92%; ^1^H NMR (DMSO_d-6_, 600 MHz), δ (ppm): 4.10 (s, 3H, CH_3_), 5.21 (s, 2H, CH_2_), 6.80–6.88 (m, 2H, H_arom_), 7.40–7.50 (m, 1H, H_arom_), 7.65–7.70 (m, 2H, H_arom_), 7.72–7.80 (m, 1H, H_arom_), 7.95–7.98 (m, 2H, H_arom_), 7.98–8.05 (m, 2H, H_arom_), 8.58 (s, 1H, C=CH), 8.87–8.95 (m, 1H, H_arom_), 9.02 (s, 1H, H6), 11.07 (s, 1H, NH); ^13^C NMR (DMSO_d-6_, 150.9 MHz), δ (ppm): 42.87 (CH_3_), 61.81 (CH_2_), 105.13, 113.63, 114.64, 115.84, 118.62, 119.04, 120.31, 122.33, 123.62, 124.47, 127.99, 129.91, 130.38, 133.56, 134.79, 135.79, 139.11, 143.11, 144.06, 151.14, 157.29; ESI-HRMS Calcd for C_25_H_19_ClN_5_OS ([M]^+^): 472.0999, found: 472.0969.

14. 5-Methyl-10-(1-(4-chlorophenyl)-1H-1,2,3-triazol-4-yl)methoxy-12H-quino [3,4-b][1,4]benzothiazinium chloride (**2n**): Procedure A. Yield: 48%; ^1^H NMR (DMSO_d-6_, 600 MHz), δ (ppm): 4.15 (s, 3H, CH_3_), 5.22 (s, 2H, CH_2_), 6.79–6.85 (m, 1H, H_arom_), 6.98–7.02 (m, 1H, H_arom_), 7.40–7.45 (m, 1H, H_arom_), 7.65–7.72 (m, 2H, H_arom_), 7.79–7.87 (m, 1H, H_arom_), 7.92–8.10 (m, 4H, H_arom_), 8.68 (s, 1H, C=CH), 9.02–9.11 (m, 1H, H_arom_), 9.18 (s, 1H, H6), 11.18 (s, 1H, NH); ^13^C NMR (DMSO_d-6_, 150.9 MHz), δ (ppm): 43.14 (CH_3_), 61.51 (CH_2_), 106.60, 107.04, 107.69, 113.61, 116.14, 119.20, 122.30, 123.83, 124.59, 128.06, 128.28, 130.38, 133.54, 134.85, 135.79, 137.99, 139.07, 143.70, 143.88, 151.80, 158.51; ESI-HRMS Calcd for C_25_H_19_ClN_5_OS ([M]^+^): 472.0999, found: 472.0976.

### 3.2. X-ray Structural Analysis

C_26_H_22_N_5_OS^+^Cl^–^, *M*_r_ = 487.99, red plate, 0.038 × 0.164 × 0.293 mm, monoclinic, space group *Cc*, *a* = 4.4070(7), *b* = 27.253(4), *c* = 22.678(3) Å, *β*= 94.224(5)°, *V* = 2716.3(7) Å^3^, *Z* = 4, *D*_c_ = 1.193 g·cm^–3^, *F*(000) = 1016, Bruker AXS D8 VENTURE, Mo*Kα* radiation, *λ* = 0.71073 Å, *T* = 293(2) K, 2*θ*_max_ = 55.38°, 32,586 reflections collected, 5911 unique (*R*_int_ = 0.081). The structure was solved and refined using the XT, VERSION 2018/2 and SHELXL-2019/1 [50] programs, respectively. Final *GooF* = 1.13, *R* = 0.105, *wR* = 0.248, *R* indices based on 4025 reflections with *I* > 2*σ*(*I*) (refinement on *F*^2^), 328 parameters, 0 restraints. Lp and absorption corrections applied, *μ* = 0.24 mm^–1^.

### 3.3. Biological Evaluation

#### 3.3.1. Cell Culture

Compounds were evaluated for their antiproliferative activity using three cultured cell lines: A549 (human lung carcinoma, ATCC, Manassas, VA, USA), SNB-19 (human glioblastoma, DSMZ, German Collection of Microorganisms and Cell Cultures), T-47D (human breast cancer, ATCC, Manassas, VA, USA), and NHDF (normal human dermal fibroblasts, ATCC, Manassas, VA, USA). The cultured cells were kept at 37 °C and 5% CO_2_. The cells were seeded (1 × 10^4^ cells/well/100 μL DMEM supplemented with 10% FCS, streptomycin, and penicillin) using 96-well plates (corning). Cells were counted in a hemocytometer (Burker chamber) using a phase contrast Olympus IX50 microscope equipped with a Sony SSC-DC58 AP camera and an Olympus DP10 digital camera.

#### 3.3.2. Proliferation Assay

The antiproliferative effect of the compounds exerted on cancer and normal cells was determined using the Cell Proliferation Reagent WST-1 assay (Roche Molecular Biochemicals, Mannheim, Germany). This assay is based on a colorimetric method using the enzymatic ability of viable cells to cause the bright, red-colored stable tetrazolium monosodium salt [2-(4-iodophenyl)-3-(4-nitrophenyl)-5-(2,4-disulfophenyl)–2H-tetra-zolium] to transform into the dark, red-colored soluble formazan. A greater number of viable cells resulted in a greater overall activity of mitochondrial dehydrogenases in the measured sample. An increase in the amount of formazan dye formed correlated with the number of metabolically active cells in the culture. The formazan dye produced by metabolically active cells was quantified by absorbance readings at appropriate wavelengths. The examined cells were exposed to the tested compounds (1 mM DMSO stock) for 72 h at various concentrations (0.1–100 μM). The control was performed in order to eliminate the DMSO effect at the concentration used. Cell cultures were incubated with WST-1 (10 μL) for 1 h. The absorbance of the samples was measured against a background control at 450 nm using a microplate reader with a reference wavelength at 600 nm. The obtained results are expressed as the means of at least two independent experiments performed in triplicate. The antiproliferative activity of the tested compounds was compared to that of cisplatinum. The values of IC_50_ (a concentration of a compound that is required for 50% inhibition) were calculated from the dose-response relationship with respect to control. The results were reported as the mean (±std) of three separate experiments.

#### 3.3.3. In Vitro Antibacterial Evaluation

In vitro antibacterial activity of the synthesized compounds was evaluated against representatives of multidrug-resistant bacteria, three clinical isolates of methicillin-resistant *S. aureus*: clinical isolates of animal origin, MRSA 63718 (Department of Infectious Diseases and Microbiology, Faculty of Veterinary Medicine, University of Veterinary Sciences Brno, Czech Republic), carrying the mecA gene [51]; and MRSA SA 630 and MRSA SA 3202 [52] (National Institute of Public Health, Prague, Czech Republic), both of human origin. These three clinical isolates were classified as vancomycin-susceptible (but with a higher MIC of vancomycin equal to 2 μg/mL (VA2-MRSA), within the susceptible range for MRSA 63718) and methicillin-resistant *S. aureus* (VS-MRSA). Vancomycin- and methicillin-susceptible *S. aureus* ATCC 29213 and vancomycin-susceptible *Enterococcus faecalis* ATCC 29212, obtained from the American Type Culture Collection, were used as the reference and quality control strains. Three vanA gene-carrying vancomycin-resistant isolates of *E. faecalis* (VRE 342B, VRE 368, VRE 725B) were provided by Oravcova et al. [37].

The minimum inhibitory concentrations (MICs) were evaluated by the microtitration broth method according to the CLSI, with some modifications [53,54]. The compounds were dissolved in DMSO (Sigma, St. Louis, MO, USA) to obtain a concentration of 10 µg/mL and diluted in a microtitration plate in an appropriate medium, i.e., Cation Adjusted Mueller–Hinton Broth (CaMH, Oxoid, Basingstoke, UK) for staphylococci, and Brain Heart Infusion Broth (BHI, Oxoid) for enterococci, to reach the final concentration of 256–0.125 µg/mL. Microtiter plates were inoculated with test micro-organisms so that the final concentration of bacterial cells was 10^5^. Ampicillin and ciprofloxacin (Sigma) were used as reference drugs. A drug-free control and a sterility control were included. The plates were incubated for 24 h at 37 °C for staphylococci and enterococci. After static incubation in the darkness in an aerobic atmosphere, the MIC was visually evaluated as the lowest concentration of the tested compound, which completely inhibited the growth of the micro-organism. The experiments were repeated three times. The results are summarized in Table 3.

#### 3.3.4. In Vitro Antimycobacterial Evaluation

The compounds’ in vitro antimycobacterial activity was evaluated against *Mycobacterium marinum* CAMP 5644 and *M. smegmatis* ATCC 700084. The broth dilution micro-method in Middlebrook 7H9 medium (Difco, Lawrence, KS, USA) supplemented with ADC enrichment (Difco) was used to determine the minimum inhibitory concentration (MIC), as previously described [54]. The compounds were dissolved in DMSO (Sigma), and the final concentration of DMSO did not exceed 2.5% of the total solution composition. The final concentrations of the evaluated compounds, ranging from 256 μg/mL to 0.125 μg/mL, were obtained by twofold serial dilution of the stock solution in a microtiter plate with sterile medium. Ciprofloxacin and rifampicin (Sigma) were used as reference antibacterial drugs. Bacterial inocula were prepared by transferring colonies from culture to sterile water. The cell density was adjusted to 0.5 McFarland units using a densitometer (Densi-La-Meter, LIAP, Riga, Latvia). The final inoculum was made by a 1:1000 dilution of the suspension with sterile water. Drug-free controls, sterility controls, and controls consisting of medium and DMSO alone were included. The results were determined visually after 3 days of static incubation in the darkness at 37 °C in an aerobic atmosphere for *M. smegmatis* and after 21 days of static incubation in the darkness at 28 °C in an aerobic atmosphere for *M. marinum*. The minimum inhibitory concentrations (MICs) were defined as the lowest concentration of the compound at which no visible bacterial growth was observed. The MIC value is routinely and widely used in bacterial assays and is a standard detection limit according to the CLSI [53]. The results are summarized in Table 3.

#### 3.3.5. Determination of Minimum Bactericidal Concentrations

For the above-mentioned strains/isolates, the agar aliquot subculture method was used as a test for bactericidal agents [55,56]. After the MIC value determination, the inoculum was transferred to CaMH (Oxoid) for staphylococci, BHI (Oxoid) for enterococci, and Middlebrook 7H9 (Difco) for mycobacteria medium using a multipoint inoculator. As indicated above, the plates were incubated on a thermostat at 37 °C for the appropriate time for each micro-organism. The lowest concentration of test compound at which ≤5 colonies were obtained was then evaluated as MBC, corresponding to a 99.9% decrease in CFU relative to the original inoculum.

#### 3.3.6. MTT Assay

Compounds were prepared, as previously stated, and diluted in Middlebrook media to achieve the desired final concentrations. *Mycobacterium smegmatis* ATCC 700,084 was suspended in ODAC-supplemented Middlebrook broth at a MacFarland standard of 1.0 and then diluted 1:10, using Middlebrook broth as a diluent. The diluted mycobacteria (50 µL) were added to each well containing the compound to be tested. Diluted mycobacteria in broth free from inhibiting compounds were used as a growth control. As a positive control, ciprofloxacin (Sigma) was used. All compounds and controls were prepared in duplicate. Plates were incubated at 37 °C for 3 days. After the incubation period, 10% of the well volume of MTT (3-(4,5-dimethylthiazol-2-yl)-2,5-diphenyltetrazolium bromide) reagent (Sigma) was mixed into each well and incubated at 37 °C for 4 h in the dark. Then 100 µL of 17% sodium dodecyl sulfate in 40% dimethylformamide was added to each well. The plates were read at 570 nm. The absorbance readings from the cells grown in the presence of the tested compounds were compared with uninhibited cell growth to determine the relative percent viability. The percent inhibition was determined through the MTT assay. The percent viability is calculated through a comparison of a measured value against that of the uninhibited control: % viability = OD_570E_/OD_570P_ × 100, where OD_570E_ is the reading from the compound-exposed cells, while OD_570P_ is the reading from the uninhibited cells (positive control). The cytotoxic potential is determined by a percent viability of <70% [57]. Ciprofloxacin (Sigma) was used as a positive control. The results are summarized in Table 4.

#### 3.3.7. TLC-Based Lipophilicity Determination

The lipophilic studies were performed on the chromatographic plates for RP-TLC analysis purchased from Merck (Darmstadt, Germany): TLC Silica gel 60 RP-18 F_254S_. The lipophilicity determination of the investigated compounds was carried out on chromatographic plates 10 × 10 cm developed using mobile phases (50 mL) that were prepared by mixing the respective amounts of the organic modifier methanol and water. In the case of the organic modifier concentrations (volume fraction, *v*/*v*), they varied in a range from 0.60 to 1.00 in constant steps of 0.10. Using glass capillaries, five drops of compounds were applied to the same chromatographic plates. Chromatography was performed in a classical developing chamber, which had previously been saturated with mobile phase vapors for 30 min. The migration distance was 7.0 cm, which takes approximately 20 min for the complete development of the chromatographic plates. Next, the plates were dried at room temperature (23 ± 1 °C) and visualized in UV light (λ = 254 nm). All analyses were repeated in triplicate in order to calculate the averaged values of R_F_ (retardation factor) that were subsequently converted into R_M_ values.

## 4. Conclusions

To sum up, a new methodology for the synthesis of hybrid combinations of 1,2,3-triazoles with a tetracyclic quinobenzothiazinium system was proposed in order to obtain a series of new azaphenothiazine derivatives, where the 1,2,3-triazole motif is attached to different positions of the benzene ring using the methoxylene linker. In practice, the laboratory procedure relies on the reaction of triazole aniline derivatives with thioquinanthrenediinium *bis*-chloride. It should be emphasized that triazole quinobenzothiazinium derivatives cannot be synthesized directly using the propargyl quinobenzothiazinium derivatives with organic azides. The structure of novel products was confirmed by the ^1^H-NMR, ^13^C-NMR spectroscopy, and HR-MS spectrometry, respectively. Furthermore, the three-dimensional geometry of the new products and the composition of atoms in the crystal (unit cell) were determined using X-ray diffraction. 

In fact, the biological potency of the obtained triazole quinobenzothiazinium derivatives was examined as a function of the 1,2,3-triazole chemical character and the spatial arrangement of substituents (their isomeric forms) in a tetracyclic quinobenzothiazinium system. In this context, the anticancer activity profiles of the newly synthesized compounds were tested in vitro against human cancer cells of the A549, SNB-19, and T47D lines and the normal NHDF cell line. It seems that the key element determining the cytotoxic activity of such compounds is the tetracyclic quinobenzothiazinium system—the SNB-19 cell line was the most susceptible, while the T47D ones were the least sensitive to the effects of substituent change. The allyl-containing triazole ring analogs revealed very high activity against all tested cancer cell lines, whereas low cytotoxic activity was manifested by derivatives containing a chlorophenyl substituent on the triazole ring. It should be highlighted that all new molecules demonstrated little cytotoxicity to normal NHDF cells. In the case of triazole substituents containing an allyl and benzyl group, the most active were derivatives based on a substituent in positions 9 and 10 of the quinobenzothiazinium system. On the other hand, products with a *p*-nitrobenzyl and phenylthiomethyl substituent at the triazole ring do not show such a relationship.

Additional tests of antibacterial activities against methicillin-sensitive and methicillin-resistant *staphylococci*, vancomycin-sensitive and vancomycin-resistant *enterococci*, and two mycobacterial strains were also conducted. As a matter of fact, the dependence of anticancer and antibacterial activity on both the type of substituent and its position (isomeric forms) in the quinobenzothiazinium system was recorded. Interestingly, the insertion of the triazole motif increased the anticancer activity, but on the contrary, it was counterproductive for the antimicrobial activity. The anticancer activity is more pronounced than the antimicrobial one, which can be understood as a specific benefit when the drug is simultaneously able to suppress related infections arising as a result of immunodeficiency caused by cytostatic treatment. In this context, it should be noted that all of the active compounds demonstrated bactericidal activity.

In order to combine the experimental findings with in silico methods, the distance-guided property evaluation was performed using principal component analysis and hierarchical clustering analysis on the pool of the calculated descriptors. The projection of 1,2,3-triazol-quinobenzothiazine derivatives on a plane defined by PC1 and PC2 revealed that an allyl-based isomers compose the one group, while molecules with the nitrobenzyl substituent are separated from the remaining ones. The rest of the benzene-based analogs are clustered together along the first principal component with higher empirical TLC lipophilic values. Interestingly, the structural dissimilarity between the investigated molecules is also observed along the second principal component, where all *ortho*-substituted derivatives are grouped together and separated from *meta/para*-based analogs, respectively. In fact, the HCA analysis confirmed the PCA findings. Moreover, the theoretically approximated partition coefficients (clogP) were (inter-)correlated with each other and cross-compared with the empirically specified logP_TLC_ parameters.

## Figures and Tables

**Table 1:** The cytotoxic activity with standard deviation (±std) of the aniline 1,2,3-triazole derivatives **5a**,**f**,**h**,**i**.

IC_50_ [µM]				
No.	SNB-19	A549	T47D	NHDF
**5a**	>100	>100	>100	>100
**5f**	>100	>100	>100	>100
**5h**	>100	>100	>100	>100
**5i**	>100	>100	>100	>100
**CIS**	16.7 ± 1.3	3.0 ± 0.2	9.0 ± 0.7	29.9 ± 3.3
**DOX**	1.7 ± 0.2	1.3 ± 0.1	0.8 ± 0.1	5.5 ± 0.5

SNB-19 = human glioblastoma cells; A549 = lung adenocarcinoma cells; T47D = breast cancer cells; NHDF = normal human dermal fibroblasts; CIS = cisplatin; DOX = doxorubicin.

**Table 2:** In vitro studies on the cytotoxic activity (IC_50_) with a standard deviation (±std) of 1,2,3-triazole derivatives of 5-methyl-12*H*-quino [3,4-*b*][1,4] benzothiazinium chlorides **2a**–**n**.

IC_50_ [µM]				
No.	SNB-19	A549	T47D	NHDF
**2a**	0.23 ± 0.06	0.45 ± 0.08	2.1 ± 0.5	47.8 ± 3.5
**2b**	2.7 ± 0.4	1.8 ± 0.2	5.3 ± 0.6	>100
**2c**	4.1 ± 0.5	5.7 ± 0.9	7.6 ± 0.4	>100
**2d**	2.5 ± 0.1	6.3 ± 0.4	8.6 ± 0.7	>100
**2e**	3.4 ± 0.3	8.4 ± 0.6	12.5 ± 0.8	56.6 ± 4.2
**2f**	8.5 ± 0.5	11.5 ± 0.6	20.5 ± 1.4	30.5 ± 2.1
**2g**	8.4 ± 0.7	4.5 ± 0.3	11.8 ± 0.5	27.6 ± 0.6
**2h**	9.3 ± 0.5	14.6 ± 0.6	6.3 ± 0.3	>100
**2i**	4.9 ± 0.3	9.0 ± 0.4	2.8 ± 0.2	>100
**2j**	8.8 ± 0.4	10.5 ± 0.7	19.3 ± 1.2	28.7 ± 1.2
**2k**	6.1 ± 0.3	16.4 ± 0.9	10.6 ± 0.9	>100
**2l**	9.6 ± 0.8	14.1 ± 0.6	4.3 ± 0.2	>100
**2m**	23.4 ± 1.5	16.8 ± 1.2	8.9 ± 0.9	>100
**2n**	42.9 ± 1.8	14.3 ± 0.8	15.4 ± 1.1	>100
**CIS**	16.7 ± 1.3	3.0 ± 0.2	9.0 ± 0.7	29.9 ± 3.3
**DOX**	1.7 ± 0.2	1.3 ± 0.1	0.8 ± 0.1	5.5 ± 0.5

SNB-19 = human glioblastoma cells; A549 = lung adenocarcinoma cells; T47D = breast cancer cells; NHDF = normal human dermal fibroblasts; CIS = cisplatin; DOX = doxorubicin.

**Table 3:** In vitro antibacterial activities (MIC/MBC [μM]) of the studied compounds **2a**–**n** compared to ampicillin (AMP) and ciprofloxacin (CPX) and antimycobacterial activities (MIC/MBC) compared to ciprofloxacin (CPX) and rifampicin (RIF).

No.	MIC [μM]MBC [μM]									
	SA	MRSA1	MRSA2	MRSA3	EF	VRE1	VRE2	VRE3	MS	MM
**2a**	131	262	262	131	262	524	262	262	32.7	65.5
	**131**	NT	**262**	**131**	NT	NT	NT	NT	32.7	65.5
**2b**	524	>524	>524	262	>524	>524	>524	524	65.5	65.5
	NT	NT	NT	**262**	NT	NT	NT	NT	65.5	65.5
**2c**	524	524	524	524	>524	>524	>524	524	65.5	131
	NT	NT	NT	NT	NT	NT	NT	NT	65.5	131
**2d**	131	131	131	65.5	>524	>524	>524	>524	32.7	32.7
	**65.5**	**131**	**131**	**65.5**	NT	NT	NT	NT	32.7	32.7
**2e**	32.8	65.5	65.5	16.3	524	524	524	524	32.7	32.7
	**65.5**	**65.5**	**65.5**	**16.3**	NT	NT	NT	NT	32.7	32.7
**2f**	>524	>524	>524	524	>524	>524	>524	>524	65.5	65.5
	NT	NT	NT	NT	NT	NT	NT	NT	65.5	65.5
**2g**	262	262	262	65.5	262	524	262	131	32.7	65.5
	262	262	NT	65.5	NT	NT	NT	NT	65.5	131
**2h**	131	262	262	131	>524	>524	>524	>524	16.3	32.7
	131	262	262	131	NT	NT	NT	NT	16.3	32.7
**2i**	524	524	524	524	>524	>524	>524	524	65.5	131
	NT	NT	NT	NT	NT	NT	NT	NT	65.5	131
**2j**	65.5	65.5	65.5	32.7	524	524	524	524	16.3	16.3
	**65.5**	**65.5**	**65.5**	**32.7**	NT	NT	NT	NT	16.3	16.3
**2k**	65.5	65.5	65.5	16.3	524	>524	524	524	32.7	32.7
	**65.5**	**65.5**	**65.5**	**16.3**	NT	NT	NT	NT	32.7	32.7
**2l**	65.5	65.5	65.5	32.7	524	524	524	524	16.3	16.3
	**65.5**	**131**	**65.5**	**32.7**	NT	NT	NT	NT	16.3	16.3
**2m**	65.5	131	131	32.7	65.5	>524	524	524	8.19	16.3
	**65.5**	**131**	**131**	**32.7**	NT	NT	524	NT	8.19	16.3
**2n**	>524	>524	>524	262	>524	>524	>524	>524	16.3	32.7
	NT	NT	NT	**262**	NT	NT	NT	NT	16.3	32.7
**AMP**	5.72	>45.8	>45.8	45.8	2.81	11.5	11.5	11.5	–	–
	5.72	>45.8	>45.8	45.8	2.81	11.5	11.5	11.5		
**CPX**	3.02	24.1	192	24.1	1.51	1.51	3.02	193	0.38	0.38
	3.02	24.1	386	24.1	3.02	3.02	3.02	386	0.38	0.38
**RIF**	–	–	–	–	–	–	–	–	19.4	2.43
									–	–

SA = *Staphylococcus aureus* ATCC 29213; MRSA1–3 = clinical isolates of methicillin-resistant *S. aureus* SA 3202, SA 630 (National Institute of Public Health, Prague, Czech Republic) and 63718 (Department of Infectious Diseases and Microbiology, Faculty of Veterinary Medicine, University of Veterinary Sciences Brno, Czech Republic); EF = *Enterococcus faecalis* ATCC 29213 and vancomycin-resistant enterococci VRE1–3 = VRE 342B, VRE 368, and VRE 725B; MS = *Mycobacterium smegmatis* ATCC 700084; MM = *Mycobacterium marinum* CAMP 5644. NT = not tested. The real bactericidal values required by the MBC/MIC ≤ 4 rule are in bold.

**Table 4:** Lowest MIC ([μM]) values with at least 70% inhibition of *Mycobacterium smegmatis* ATCC 700084 respiratory activity.

No.	Conc.	*M. smegmatis* Respiration Inhibition [%]
**2h**	1× MIC (1× MBC)	81.4
**2j**	2× MIC (2× MBC)	87.6
**2l**	2× MIC (2× MBC)	87.3
**2m**	1× MIC (1× MBC)	87.1
**2n**	1× MIC (1× MBC)	85.9
**CPX**	32× MIC (32× MBC)	85.9

## Data Availability

Crystal data are available free of charge via http://www.ccdc.cam.ac.uk/conts/retrieving.html (accessed on 13 June 2023), or from the Cambridge Crystallographic Data Centre, 12 Union Road, Cambridge CB2 1EZ, UK; Fax: +44-1223-336-033; or e-mail: deposit@ccdc.cam.ac.uk. Deposition number: CCDC 2282631.

## Supplementary Materials

The supporting information can be downloaded at: https://www.mdpi.com/article/10.3390/ijms241713250/s1.
