# Supplementary material for: Towards Anticancer and Antibacterial Agents: Design and Synthesis of 1,2,3-Triazol-quinobenzothiazine Derivatives
Source: Int J Mol Sci. 2023 Aug 26;24(17):13250. doi: 10.3390/ijms241713250 (PMC10487436; doi:10.3390/ijms241713250)
Supplement: Supplementary file 1 [file ijms-24-13250-s001.zip › ijms-2550408-supplementary.pdf]

Supplementary materials

Towards anticancer and antibacterial agents: Design and synthesis of 1,2,3-triazol-quinobenzothiazine derivatives

Ewa Kisiel-Nawrot [1], Dominika Pindjakova [2], Malgorzata Latocha [3], Andrzej Bak [4], Violetta Kozik [4], Kinga Suwinska [5], Alois Cizek [6], Josef Jampilek [2,7]* and Andrzej Zieba [1,]*

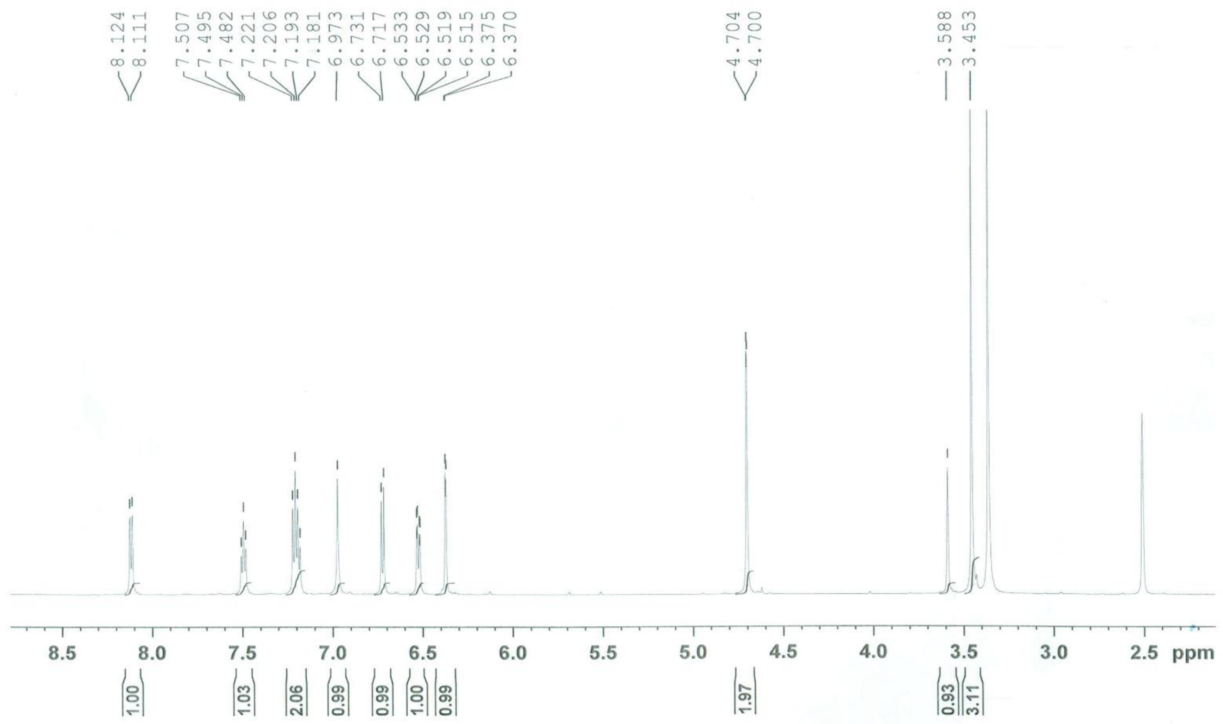

Fig. S1 ^{1}H NMR spectrum of 9-propargyloxy-5-methyl-5*H*-quino[3,4-b][1,4]benzothiazine **3a** in w DMSO.

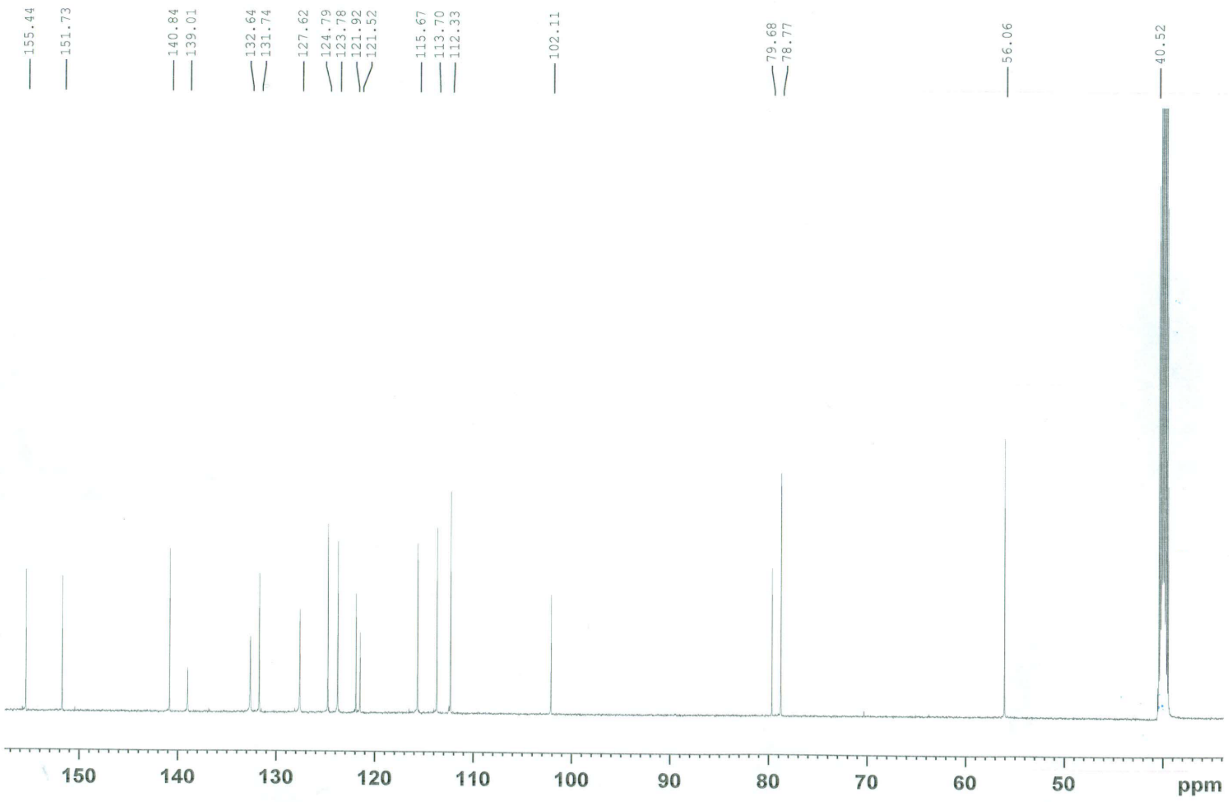

Fig. S2 ^{13}C NMR spectrum of 9-propargyloxy-5-methyl-5*H*-quino[3,4-*b*][1,4]benzothiazine **3a** in w DMSO.

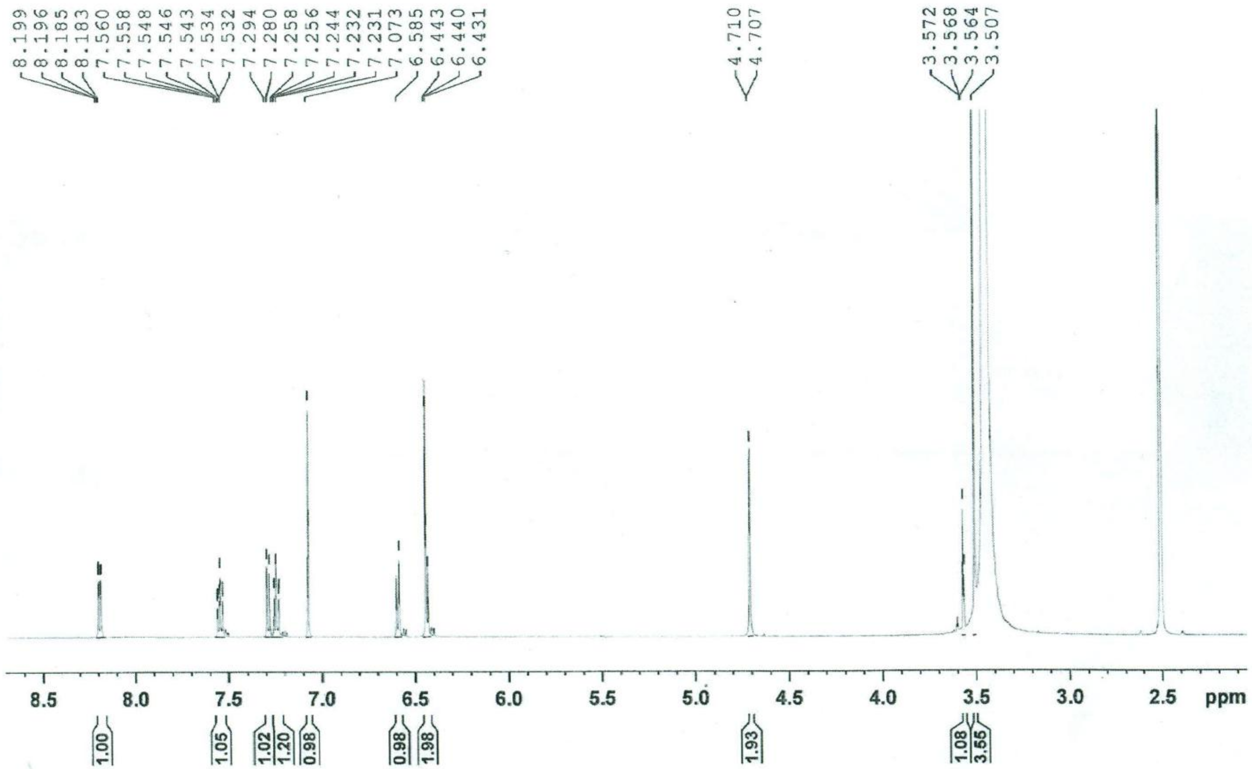

Fig. S3 ^{1}H NMR spectrum of 10-propargyloxy-5-methyl-5*H*-quino[3,4-*b*][1,4]benzothiazine **3b** in DMSO.

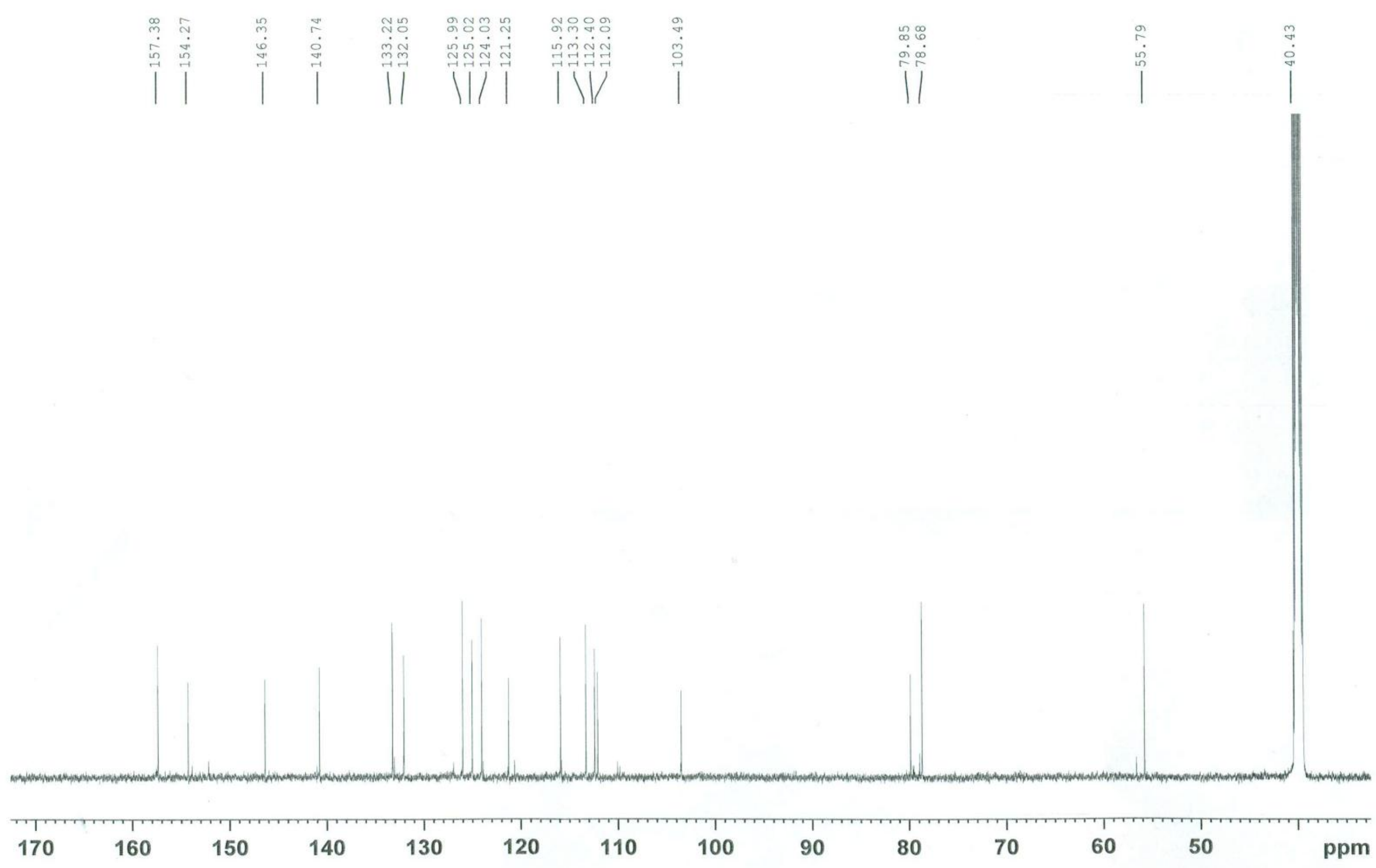

Fig. S4 ^{13}C NMR spectrum of 10-propargyloxy-5-methyl-5*H*-quino[3,4-*b*][1,4]benzothiazine **3b** in DMSO.

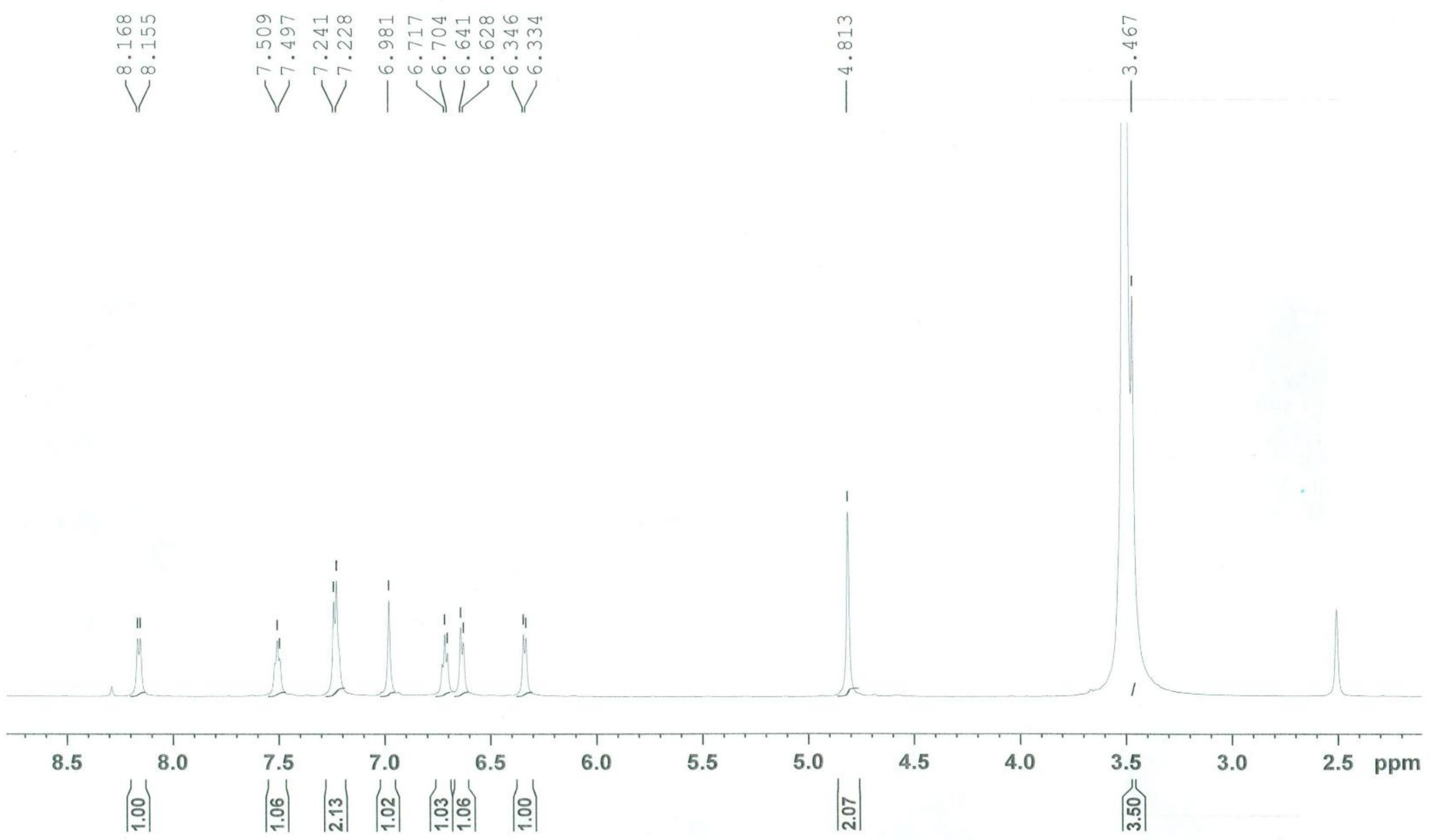

Fig. S5 ^{1}HNMR spectrum of 11-propargyloxy-5-methyl-5*H*-quino[3,4-*b*][1,4]benzothiazine **3c** in DMSO

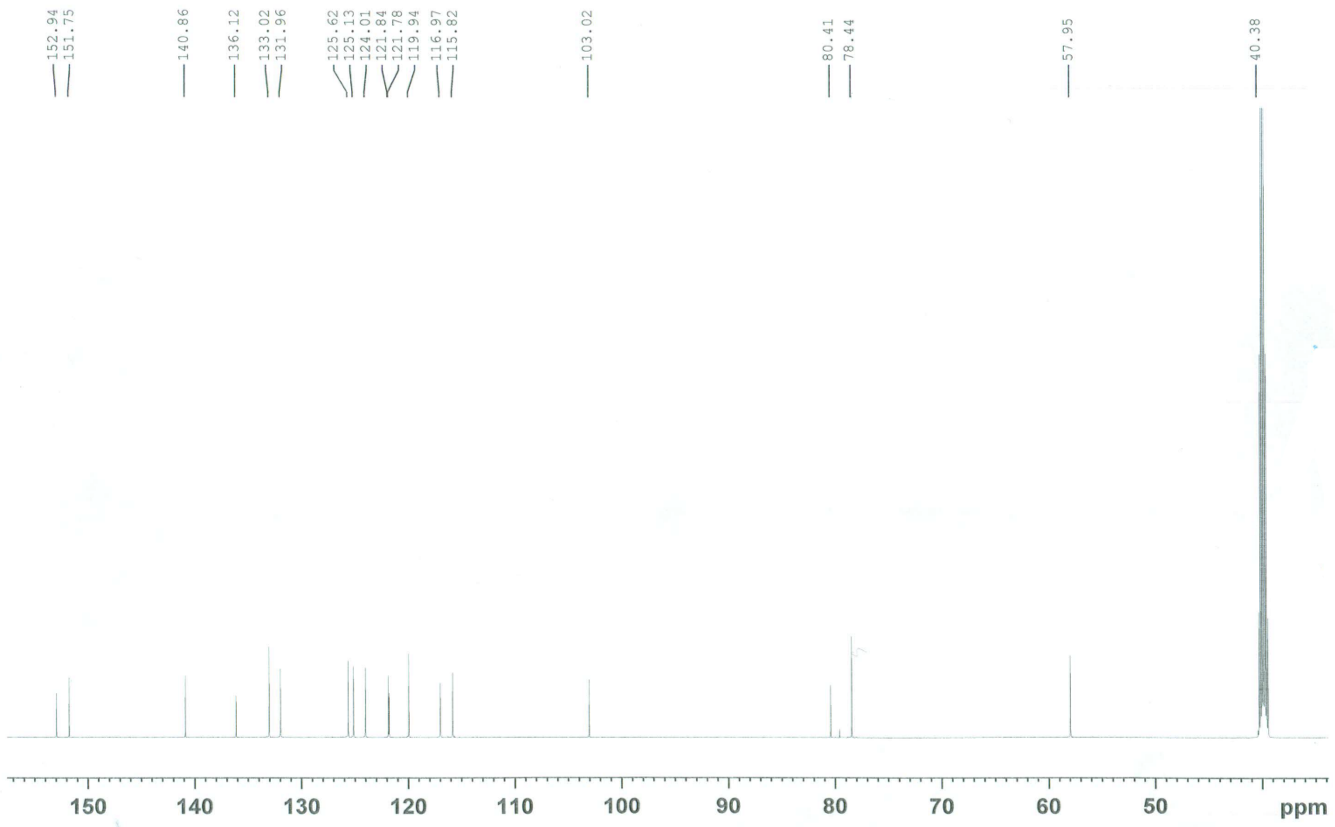

Fig. S6 ^{13}CNMR spectrum of 11-propargyloxy-5-methyl-5*H*-quino[3,4-*b*][1,4]benzothiazine **3c** in DMSO.

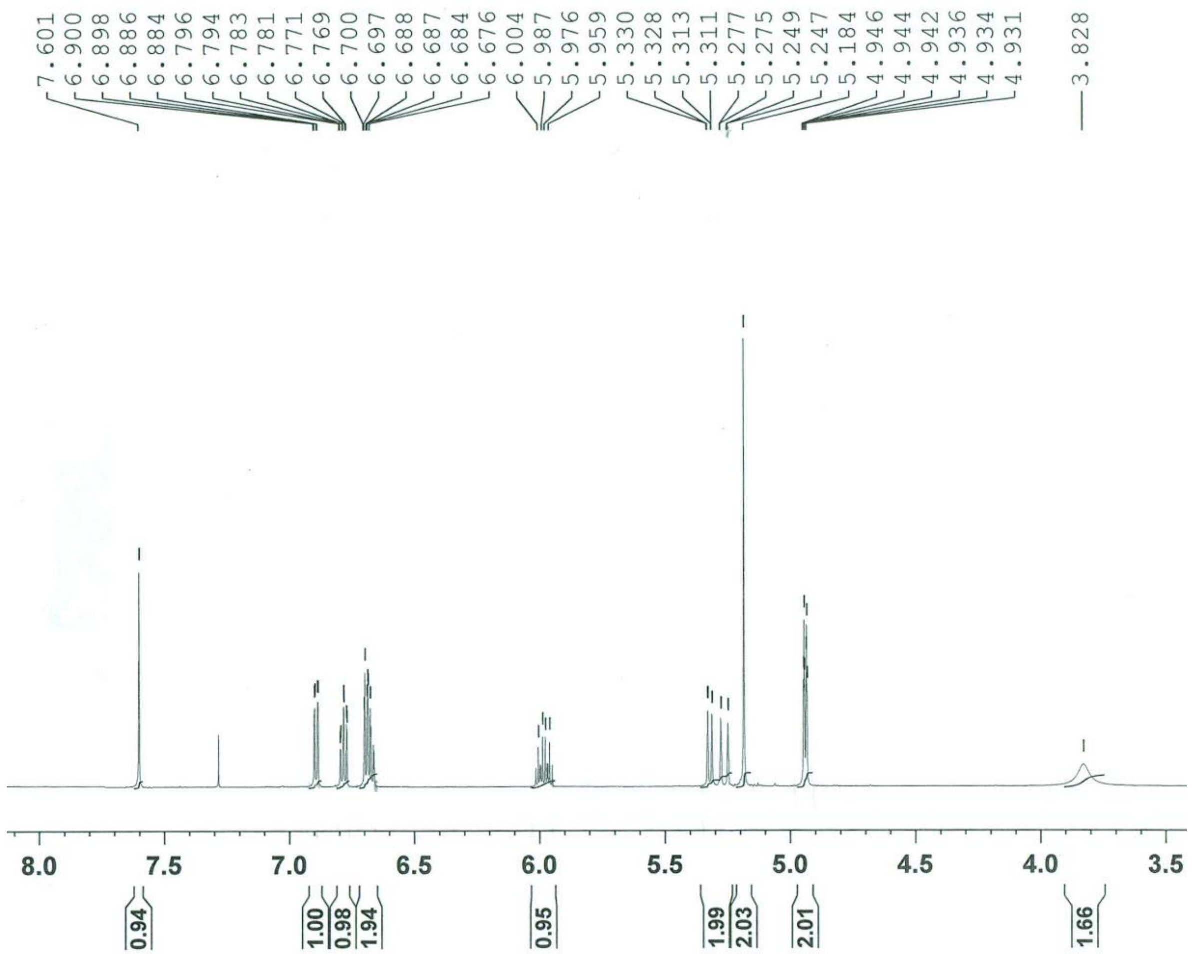

Fig. S7. H NMR Spectrum of 1-allyl-4-(2-aminophenoxy)methyl-1*H*-1,2,3-triazole **5a** in $CDCl_3$.

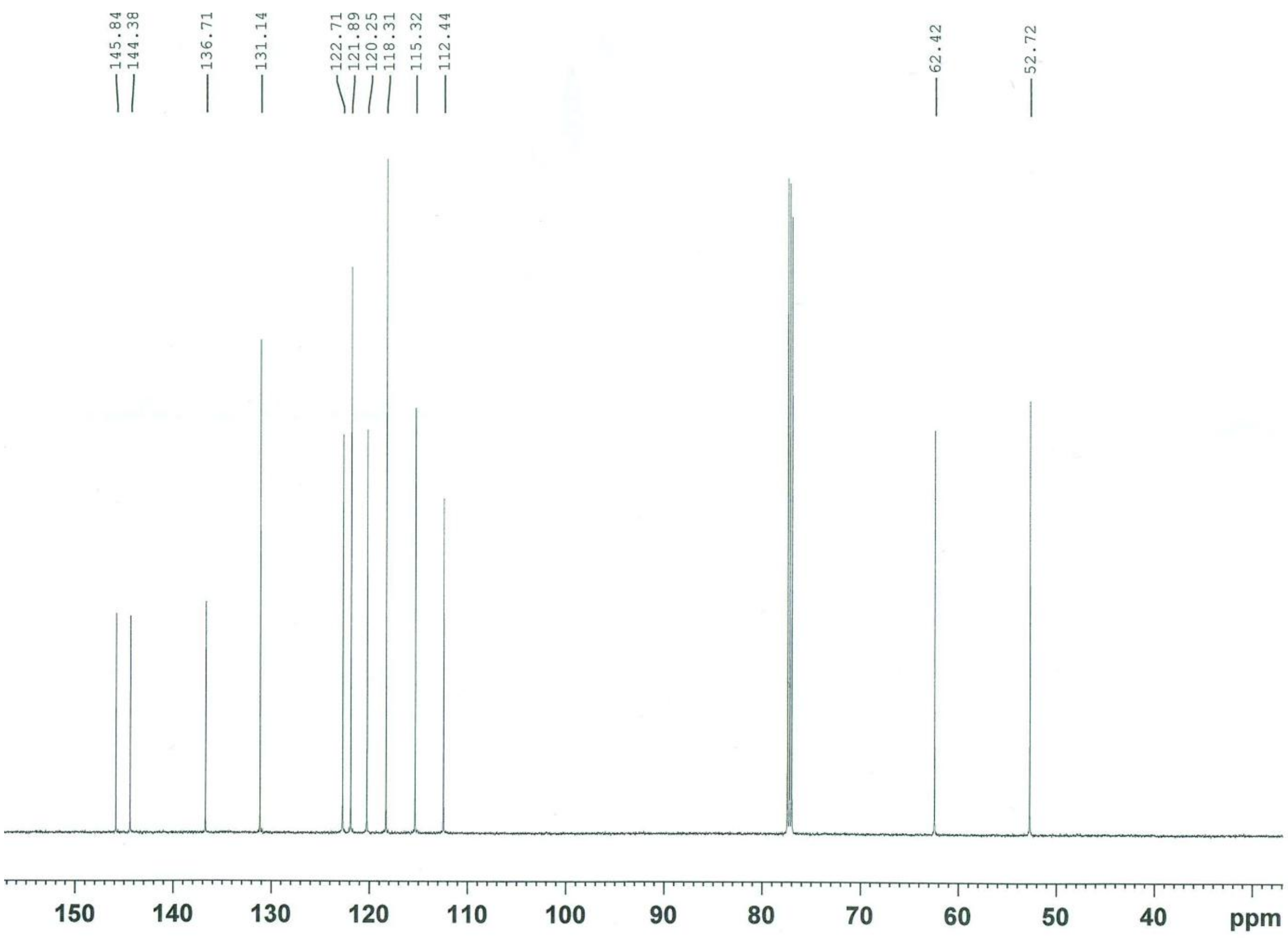

Fig. S8. ^{13}C NMR Spectrum of 1-allyl-4-(2-aminophenoxy)methyl-1*H*-1,2,3-triazole **5a** in $CDCl_3$.

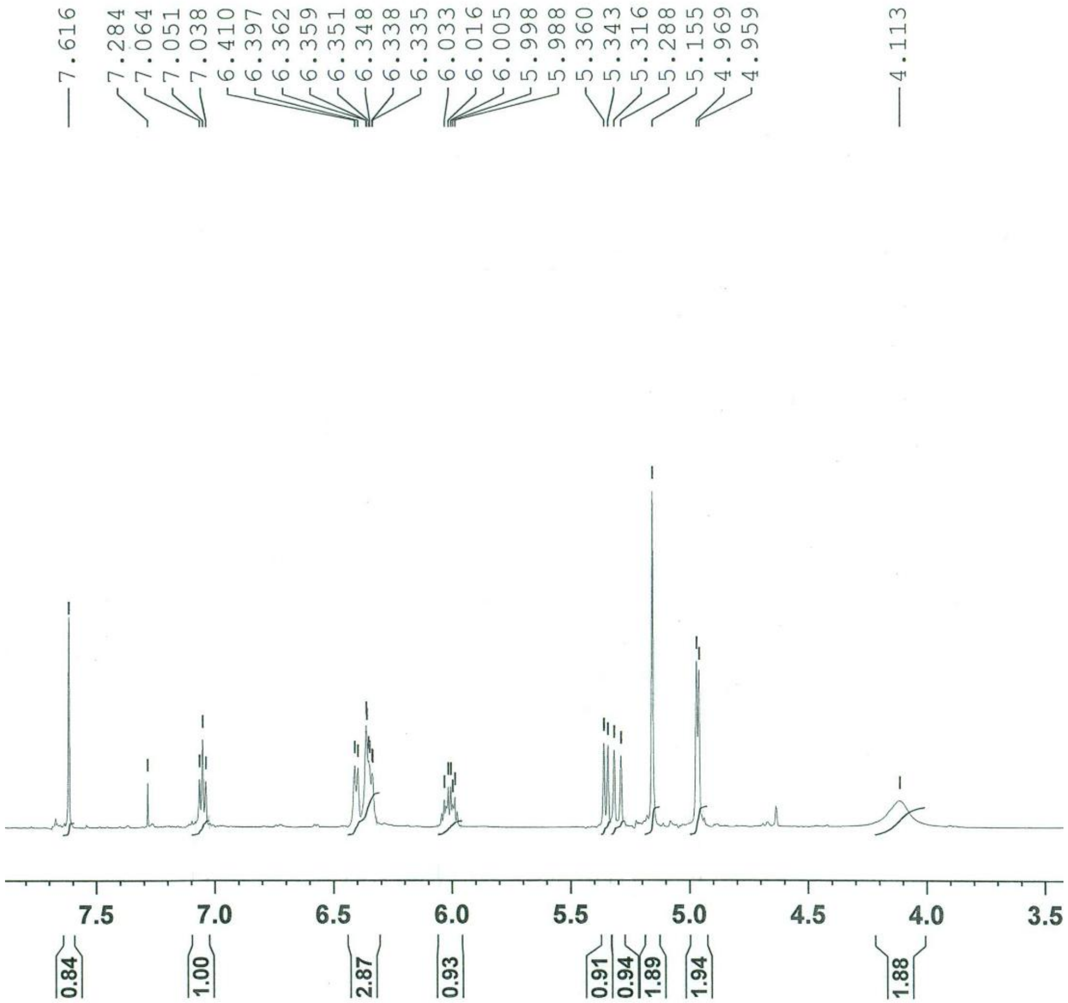

Fig. S9. ^{1}H NMR spectrum of 1-allyl-4-(3-aminophenoxy)methyl-1*H*-1,2,3-triazole **5b** in $CDCl_3$.

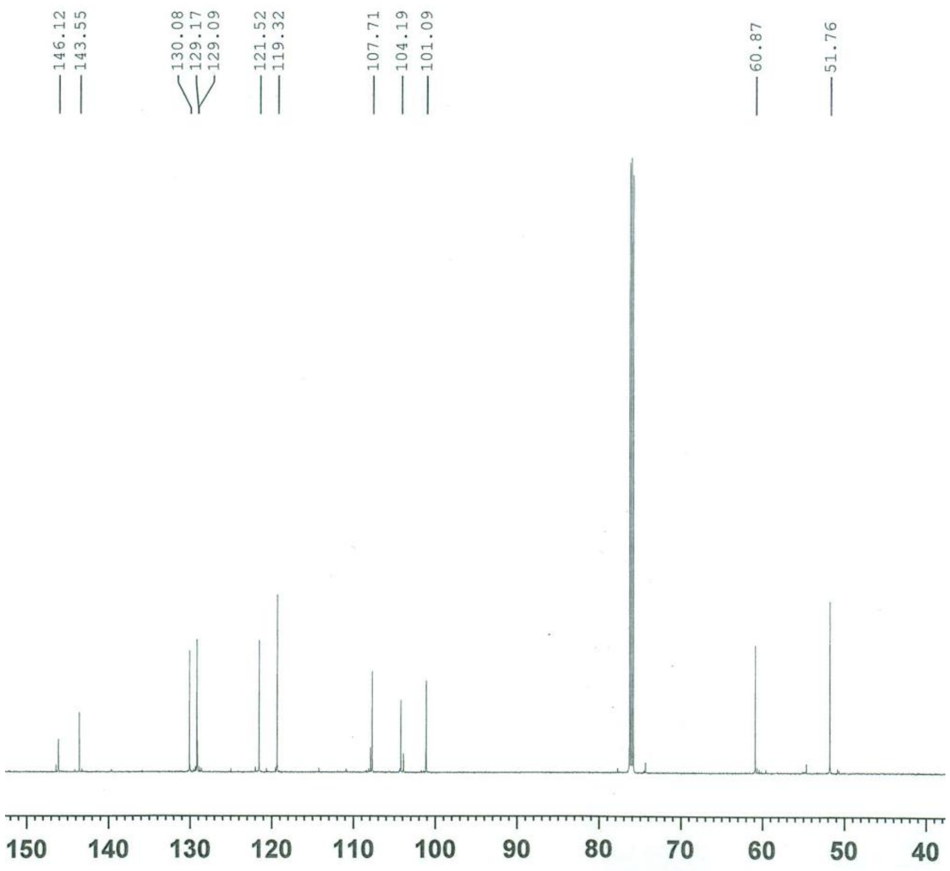

Fig. S10. ^{13}C NMR spectrum 1-allyl-4-(3-aminophenoxy)methyl-1*H*-1,2,3-triazole **5b** in $CDCl_3$

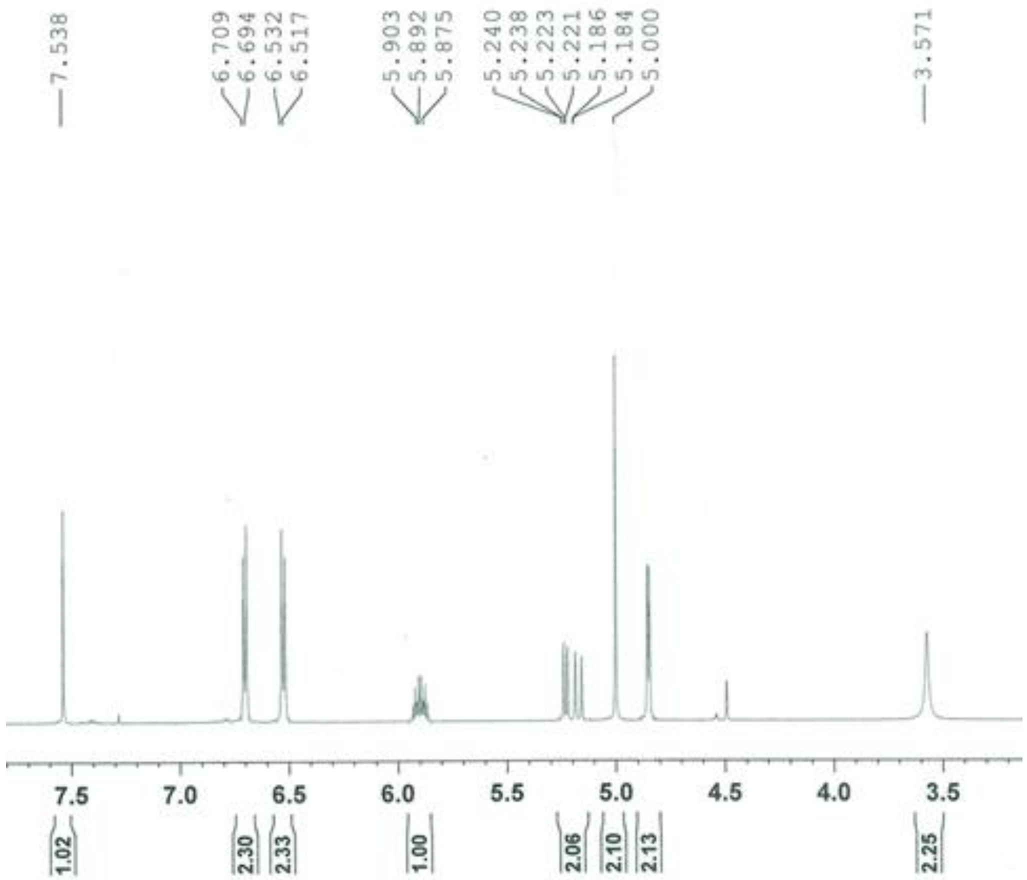

Fig. S11. ^{1}H NMR spectrum of 1-allyl-4-(4-aminophenoxy)methyl-1*H*-1,2,3-triazole **5c** in $CDCl_3$.

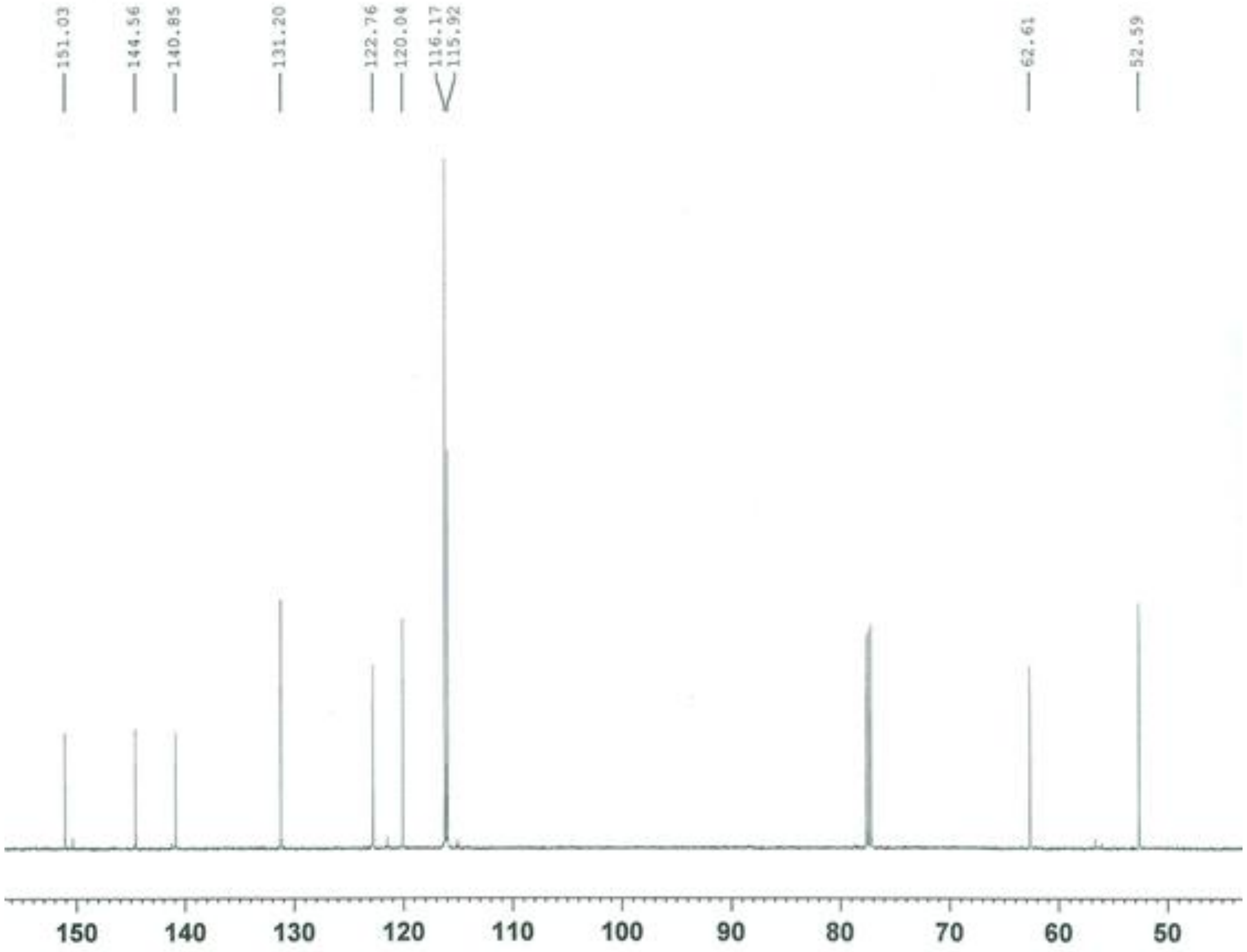

Fig. S12. ^{13}C NMR spectrum of 1-allyl-4-(4-aminophenoxy)methyl-1*H*-1,2,3-triazole **5c** in $CDCl_3$

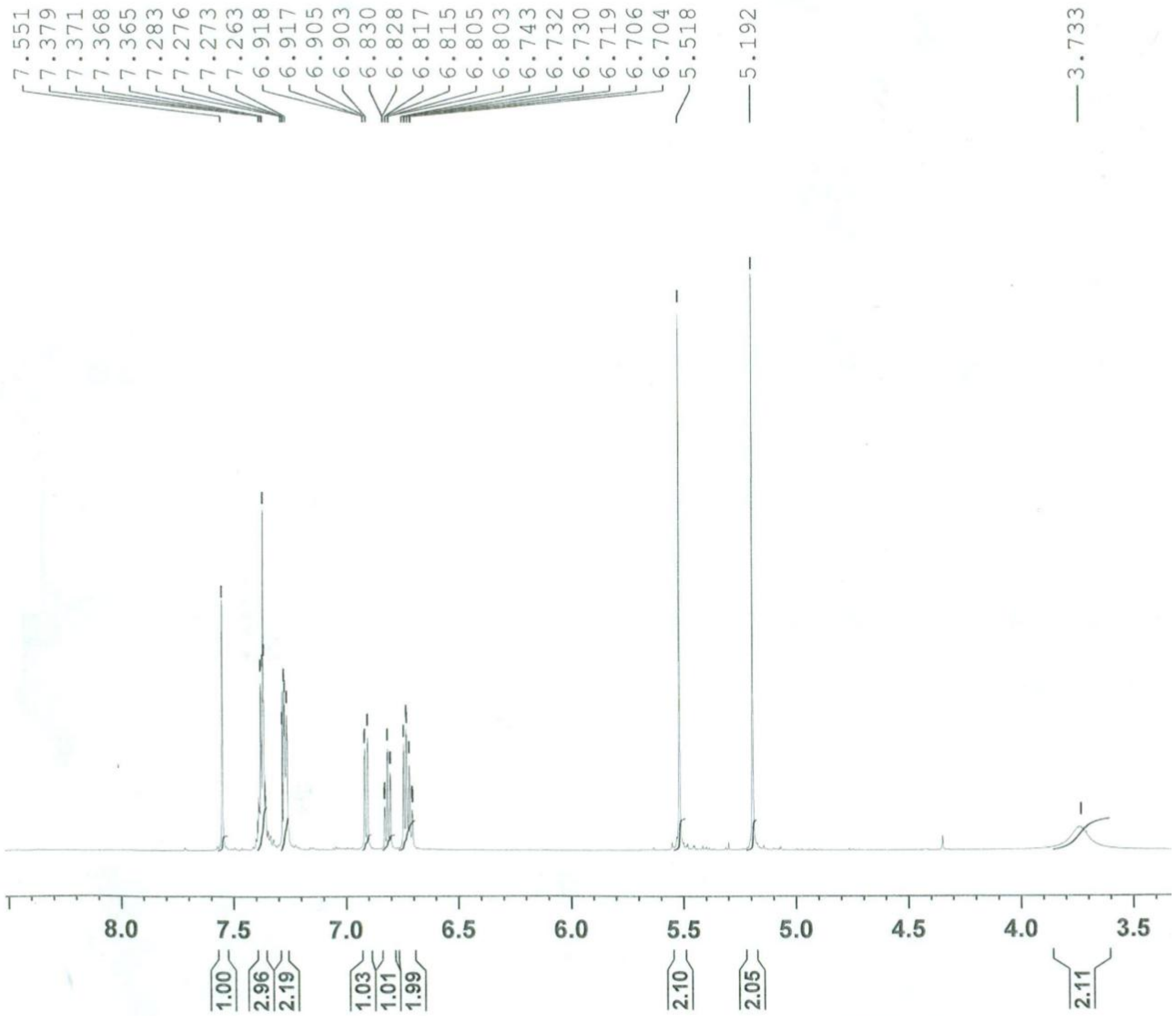

Fig. S13. ^{1}H NMR spectrum of 1-benzyl-4-(2-aminophenoxy)methyl-1*H*-1,2,3-triazole **5d** in $CDCl_3$

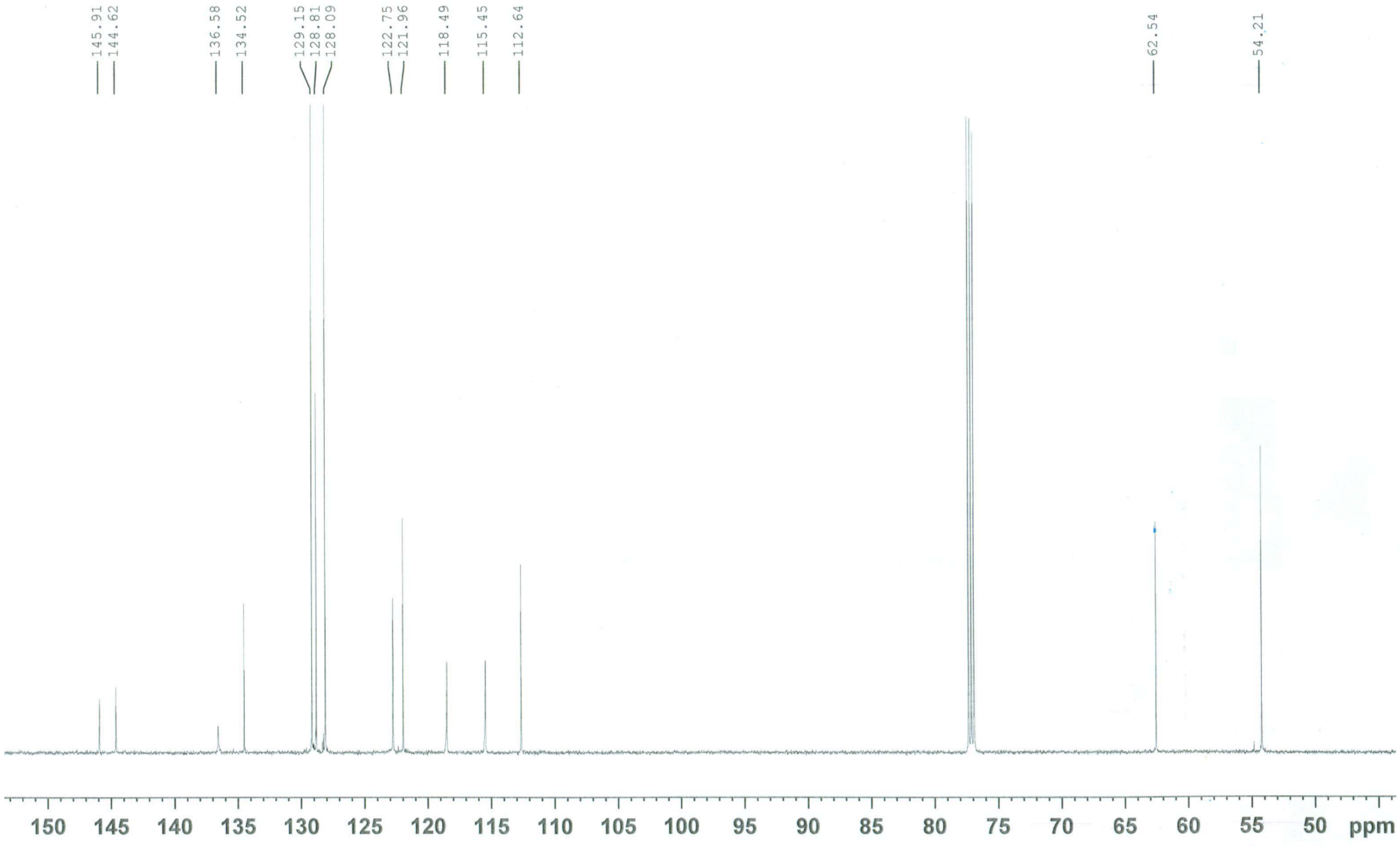

Fig. S14. ^{13}C NMR spectrum of 1-benzyl-4-(2-aminophenoxy)methyl-1*H*-1,2,3-triazole **5d** in $CDCl_3$

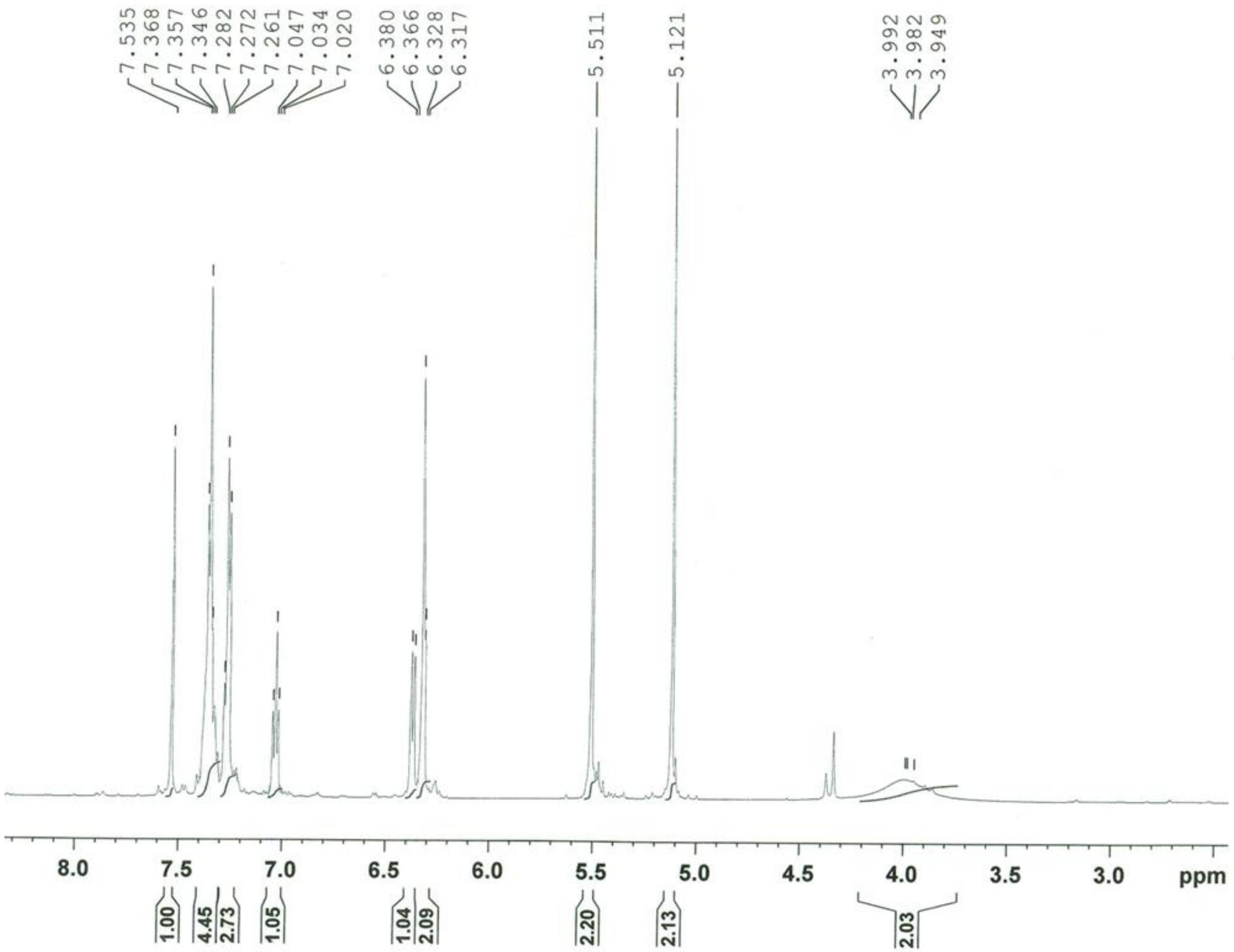

Fig. S15. ^{1}H NMR spectrum of 1-benzyl-4-(3-aminophenoxy)methyl-1*H*-1,2,3-triazole **5e** in $CDCl_3$.

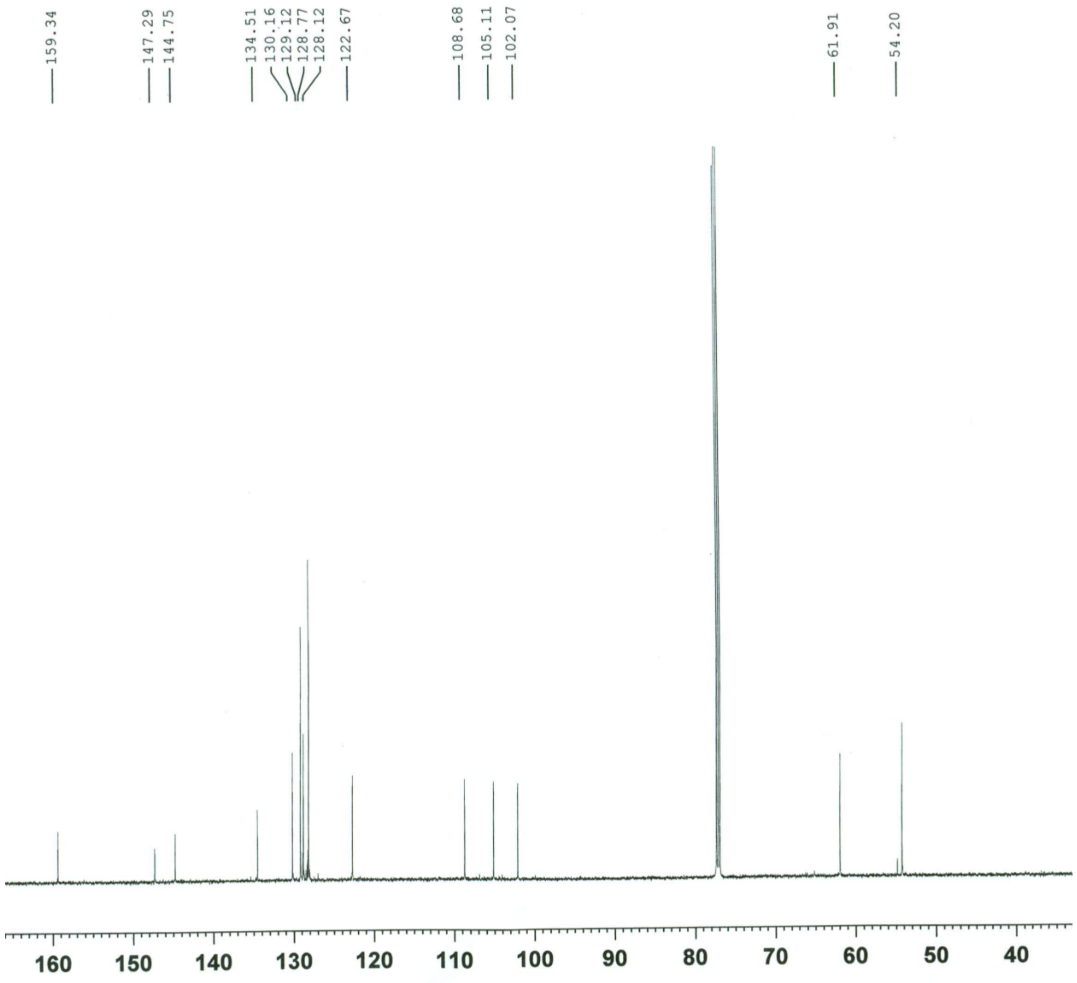

Fig. S16. ^{1}H NMR spectrum of 1-benzyl-4-(3-aminophenoxy)methyl-1*H*-1,2,3-triazole **5e** in $CDCl_3$

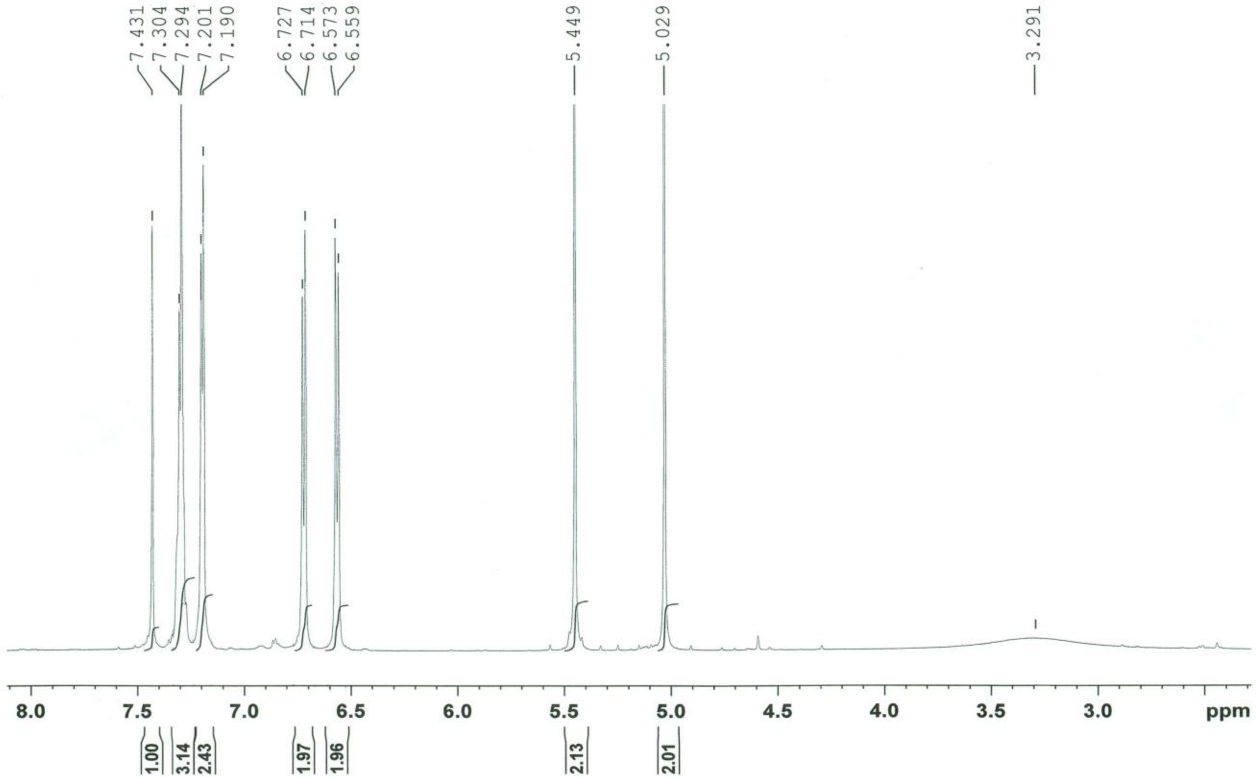

Fig. S17. ^{1}H NMR spectrum of 1-benzyl-4-(4-aminophenoxy)methyl-1H-1,2,3-triazole **5f** in $CDCl_3$

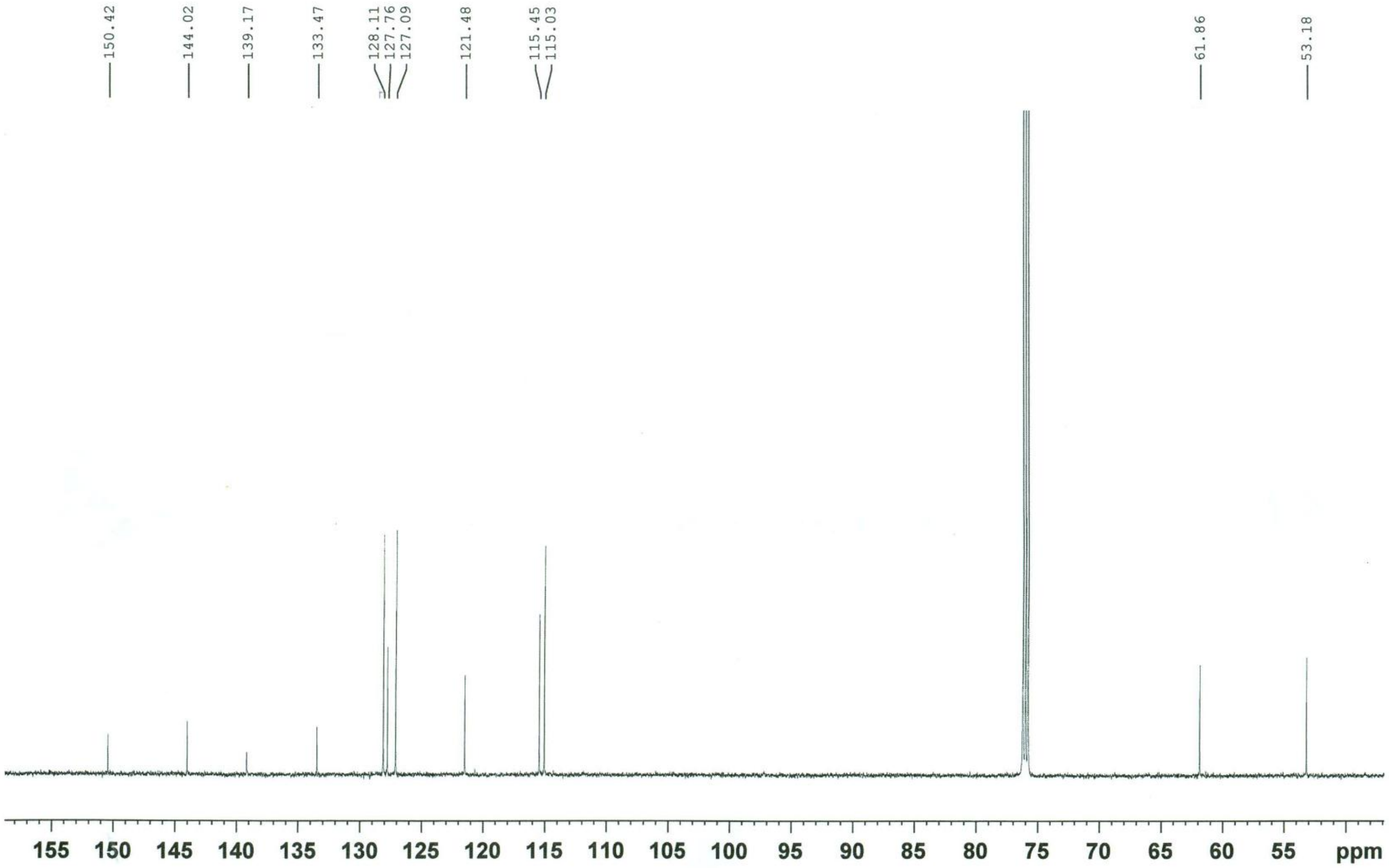

Fig. S18. ^{13}C NMR spectrum of 1-benzyl-4-(4-aminophenoxy)methyl-1*H*-1,2,3-triazole **5f** in $CDCl_3$

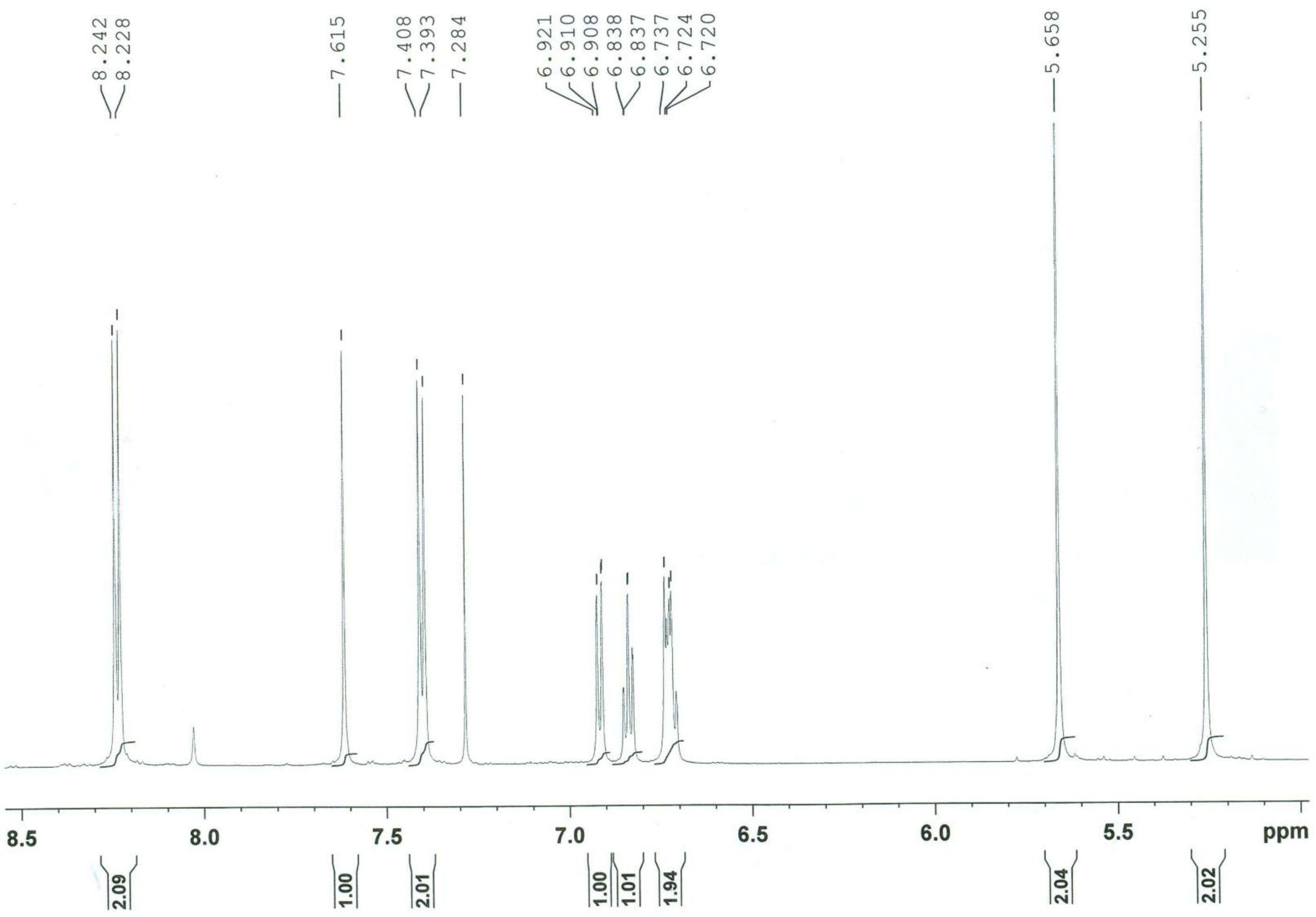

Fig. S19. ^{1}H NMR spectrum of 1-nitrobenzyl-4-(2-aminophenoxy)methyl-1*H*-1,2,3-triazole **5g** in $CDCl_3$

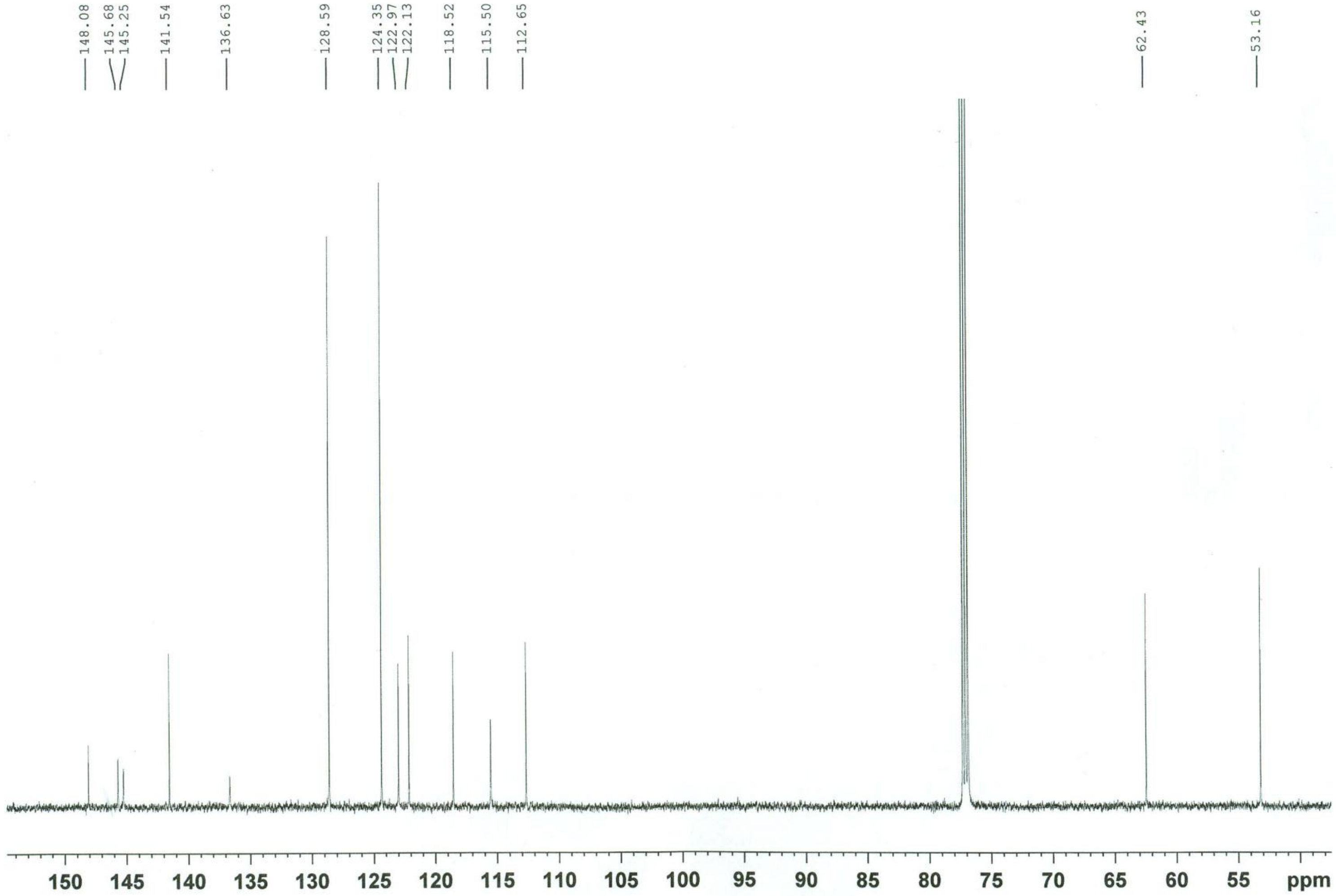

Fig. S20. ^{1}H NMR spectrum of 1-nitrobenzyl-4-(2-aminophenoxy)methyl-1*H*-1,2,3-triazole **5g** in $CDCl_3$

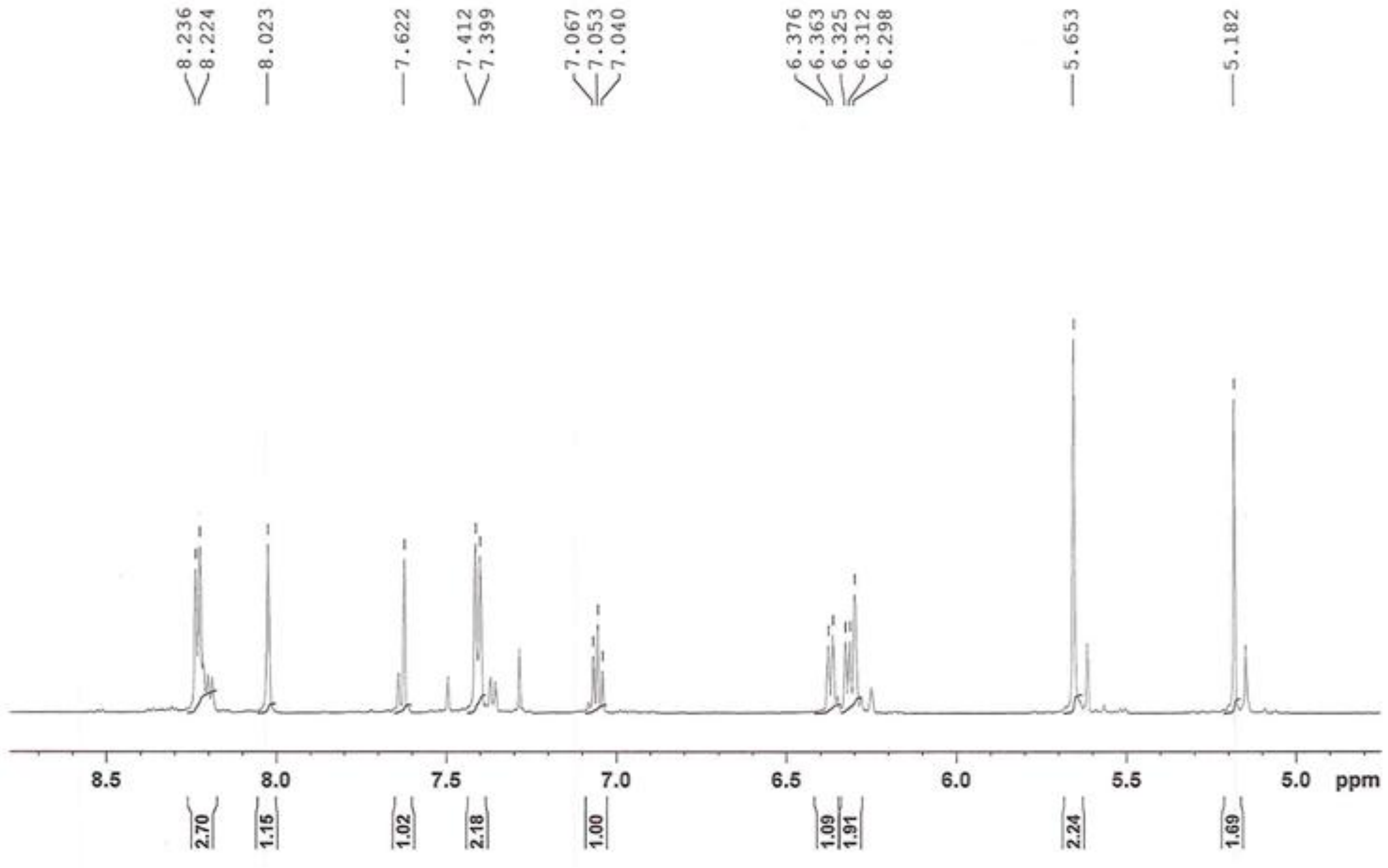

Fig. S21. ^{1}H NMR spectrum of 1-nitrobenzyl-4-(3-aminophenoxy)methyl-1*H*-1,2,3-triazole **5h** in $CDCl_3$

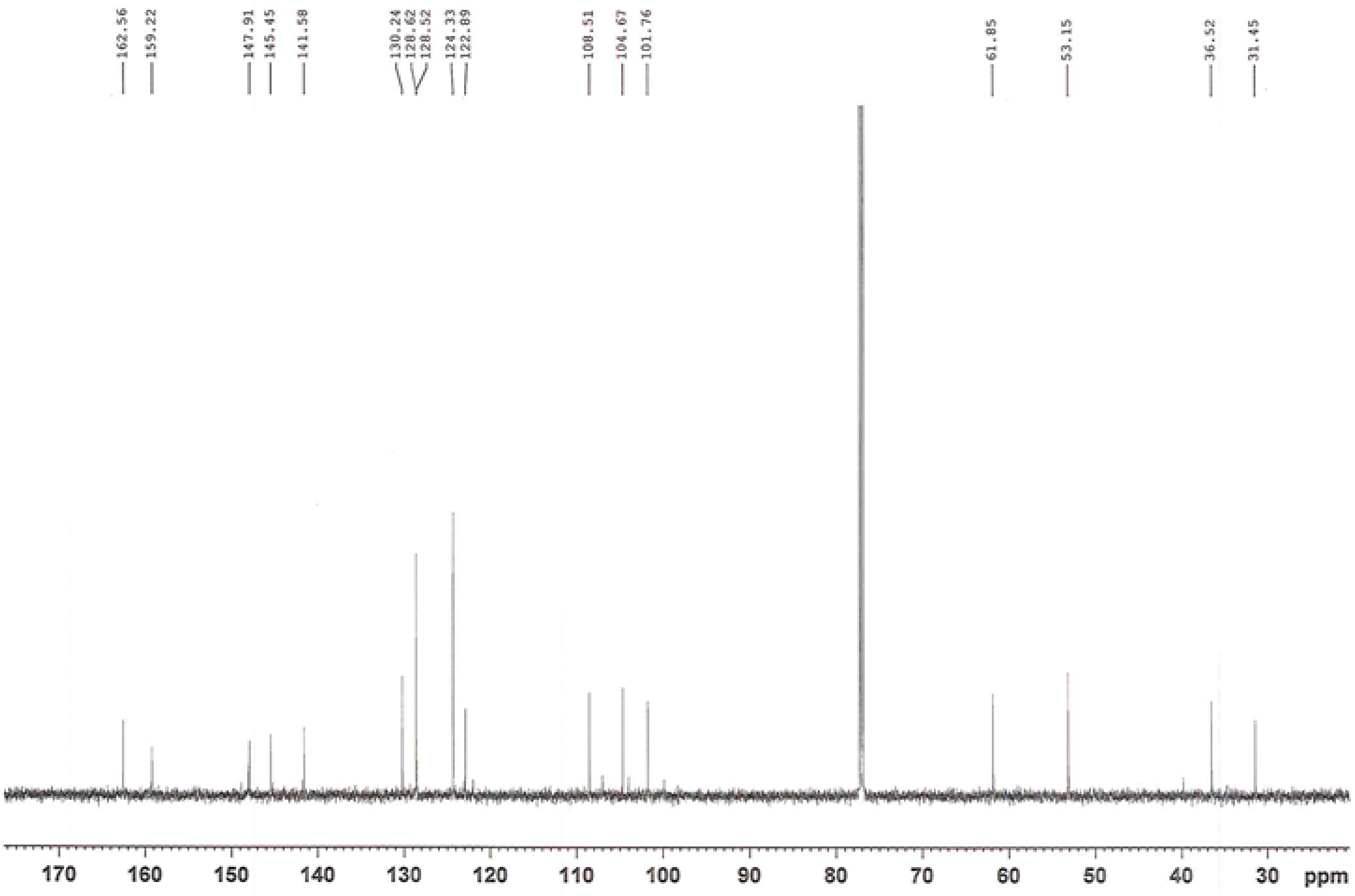

Fig. S22 ^{13}C NMR spectrum of 1-(4-nitrobenzyl)-4-(3-aminophenoxy)methyl-1*H*-1,2,3-triazole **5h** in $CDCl_3$

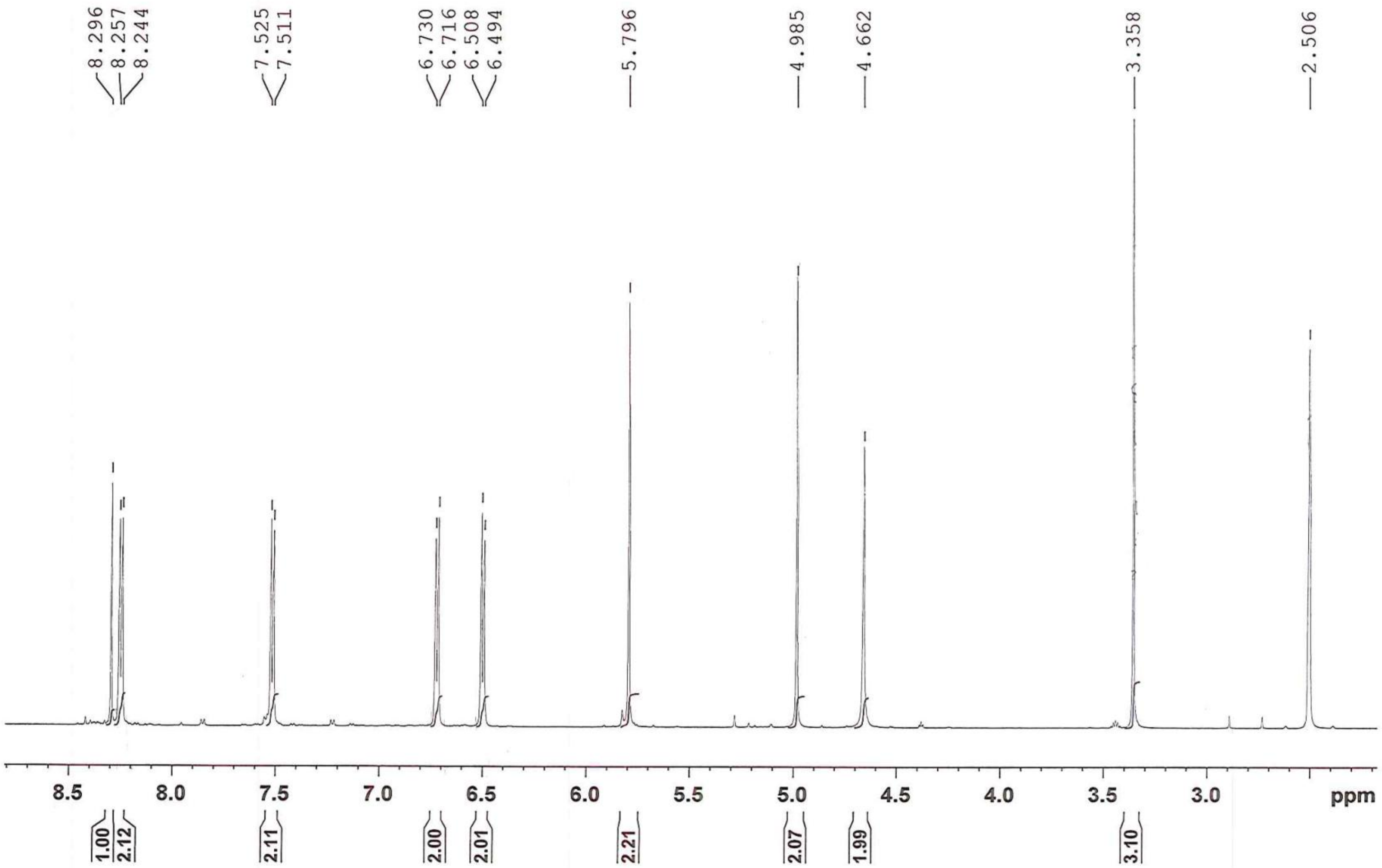

Fig.S23 ^{1}H NMR spectrum of 1-nitrobenzyl-4-(4-aminophenoxy)methyl-1*H*-1,2,3-triazole **5i** in DMSO.

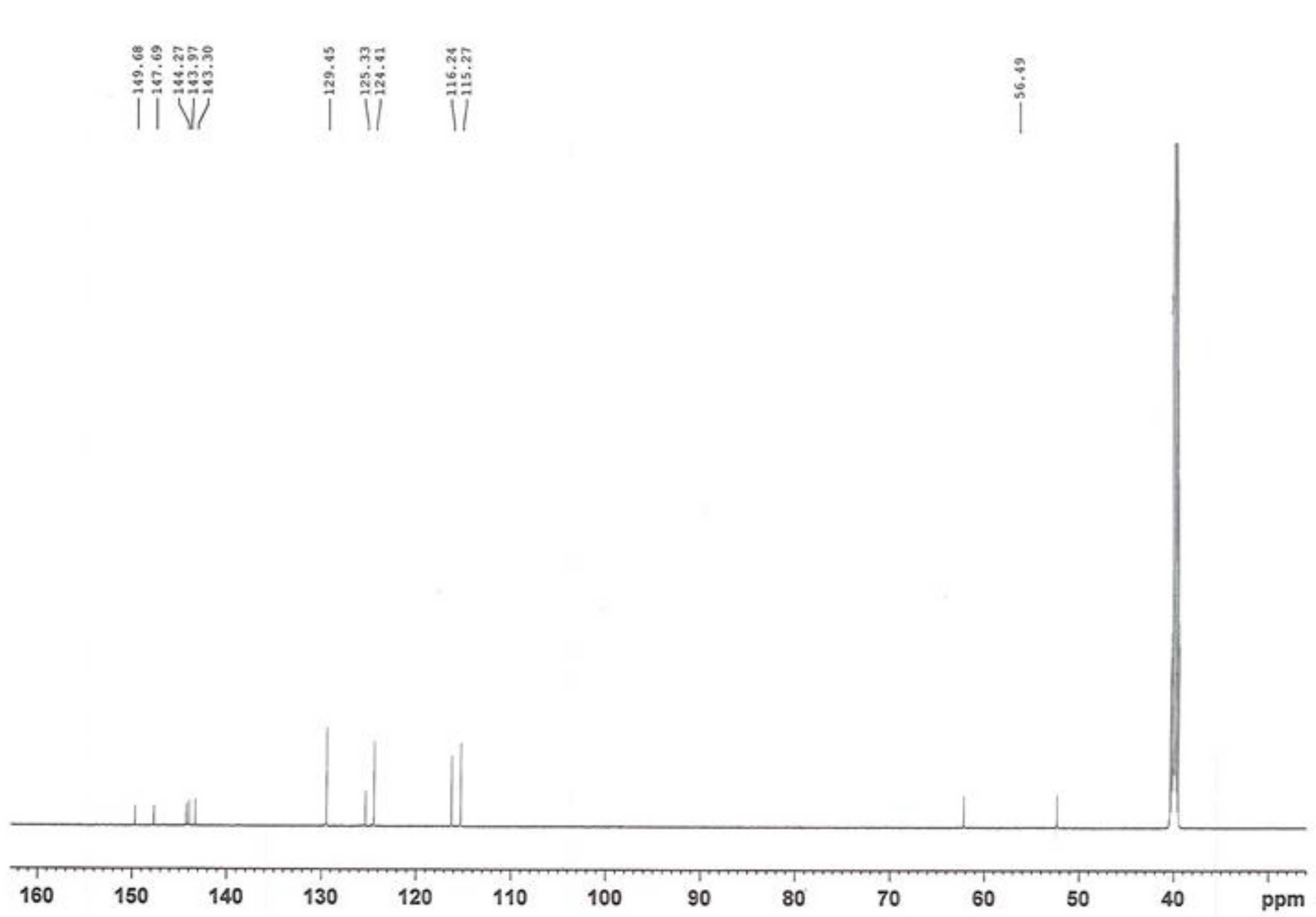

Fig. S24 ^{13}C NMR spectrum of 1-(4-nitrobenzyl)-4-(4-aminophenoxy)methyl-1*H*-1,2,3-triazole **5i** in DMSO.

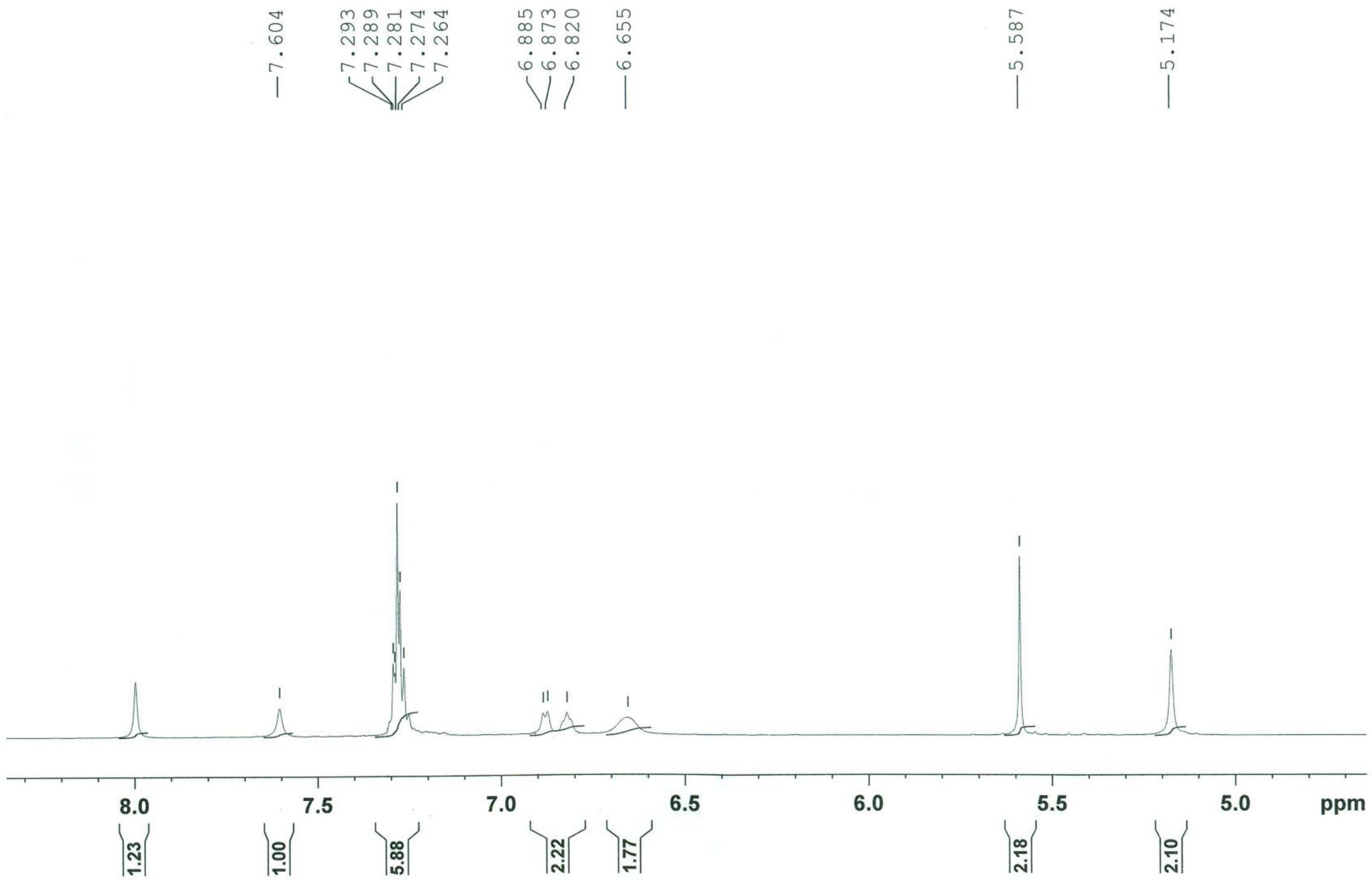

Fig. S25 ^{1}H NMR spectrum of 1-(phenylthio)methyl-4-(2-aminophenoxy)methyl-1*H*-1,2,3-triazole **5j** in w $CDCl_3$.

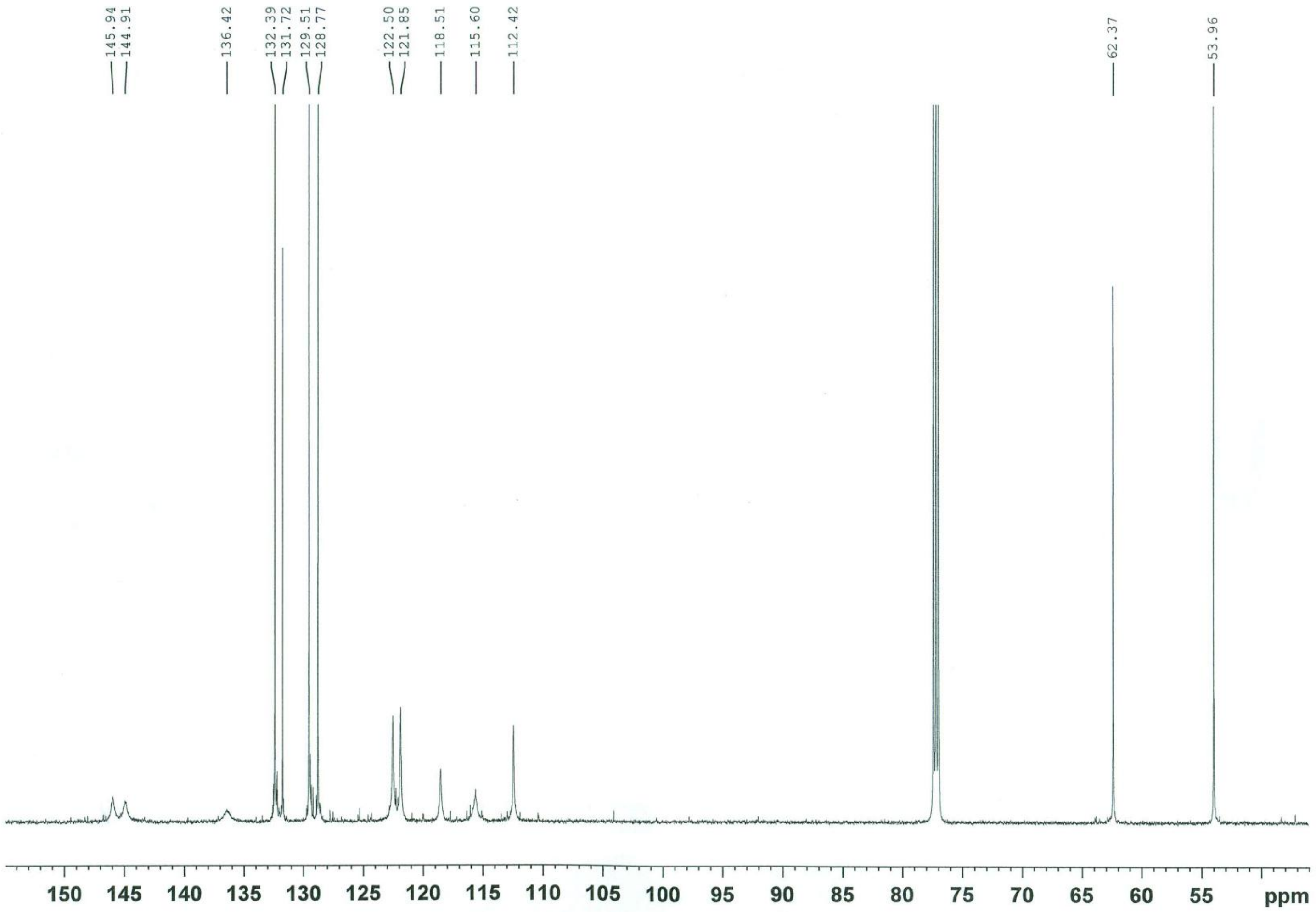

Fig. S26 ^{13}C NMR spectrum of 1-(phenylthio)methyl-4-(2-aminophenoxy)methyl-1*H*-1,2,3-triazole **5j** in w $CDCl_3$

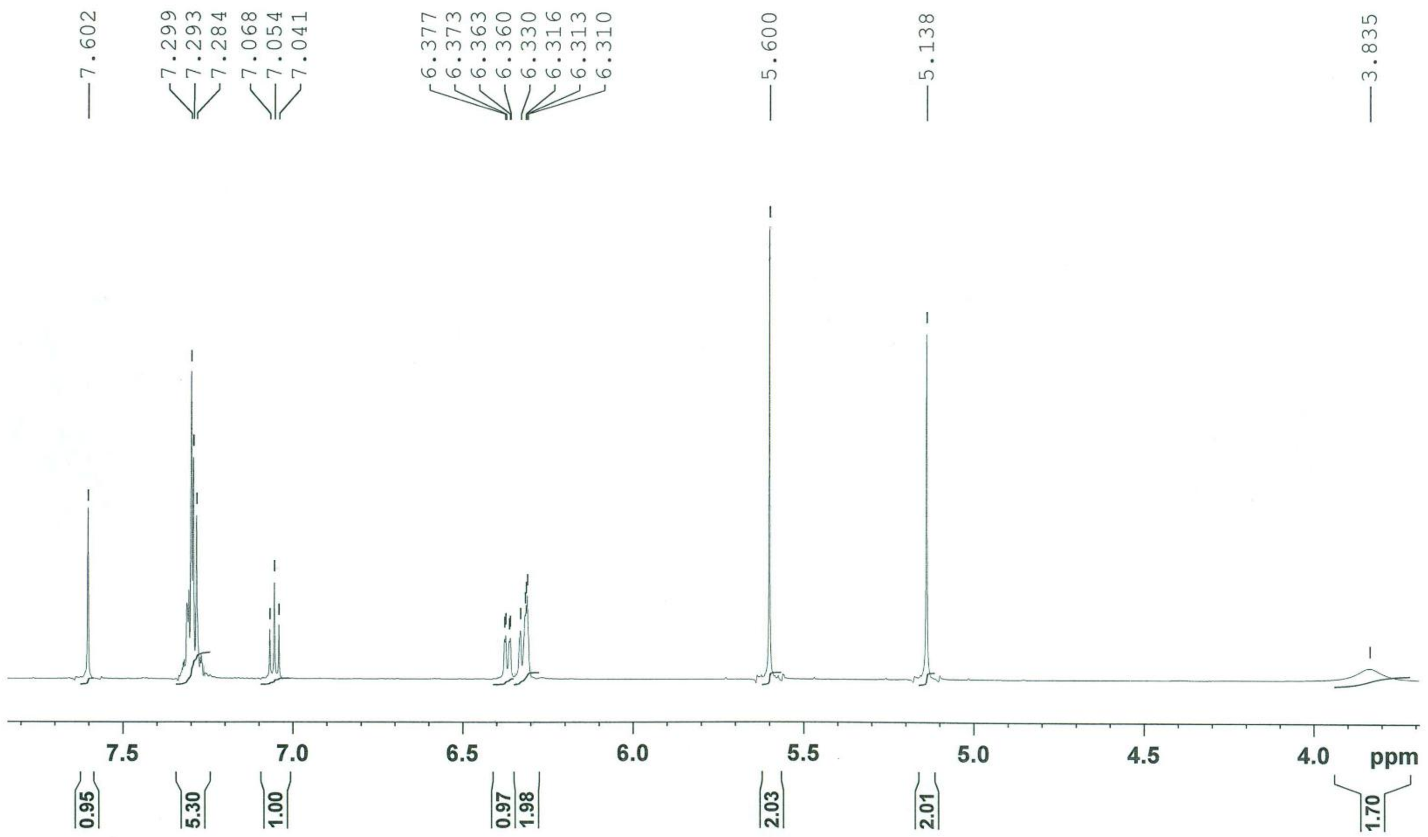

Fig. S27 ^{1}H NMR spectrum of of 1-(phenylthio)methyl-4-(3-aminophenoxy)methyl-1*H*-1,2,3-triazole **5k** in w $CDCl_3$

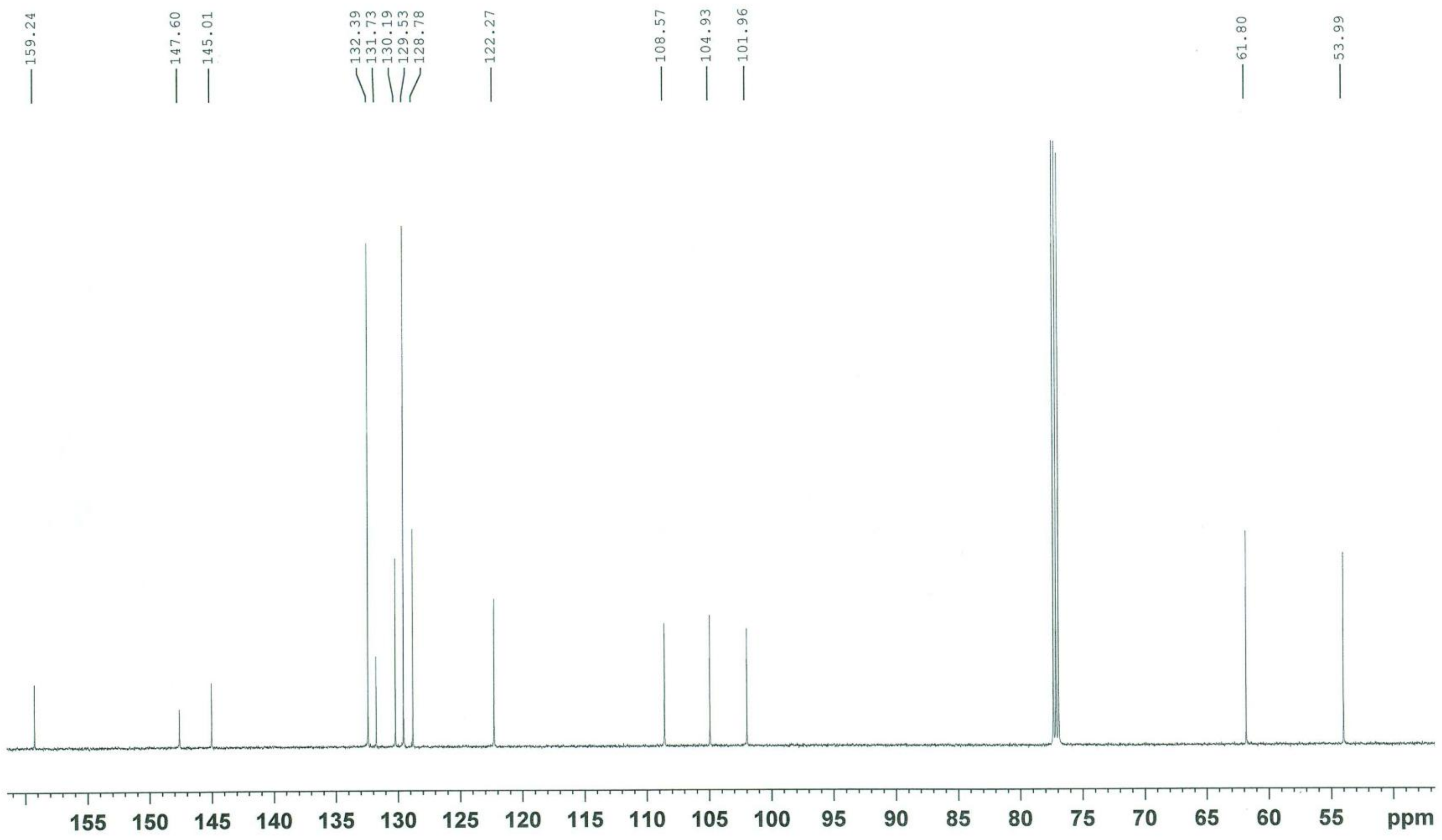

Fig. S28 ^{13}C NMR spectrum of of 1-(phenylthio)methyl-4-(3-aminophenoxy)methyl-1*H*-1,2,3-triazole **5k** in w $CDCl_3$

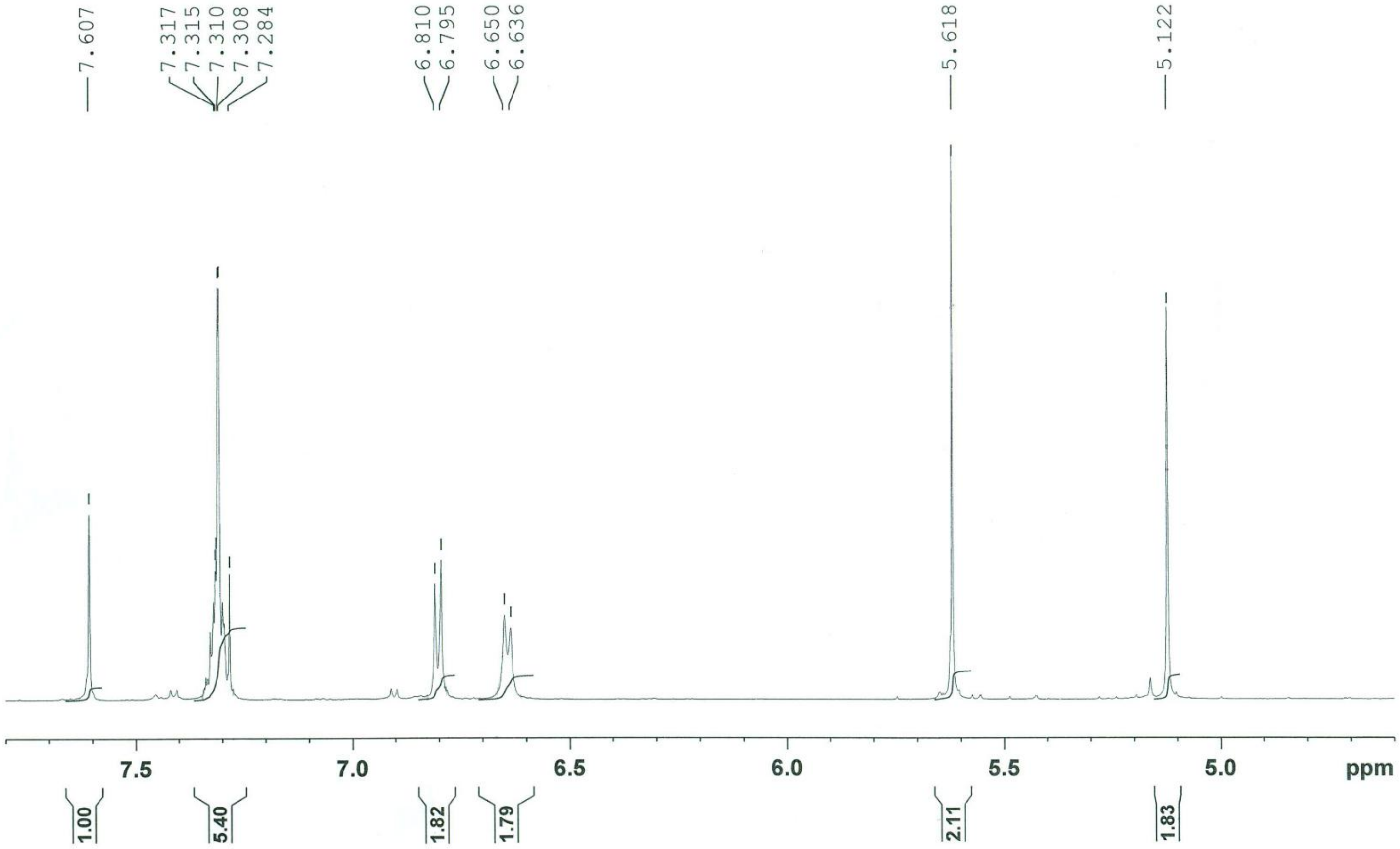

Fig. S29 ^{1}H NMR spectrum of of 1-(phenylthio)methyl-4-(4-aminophenoxy)methyl-1*H*-1,2,3-triazole **5l** in w $CDCl_3$

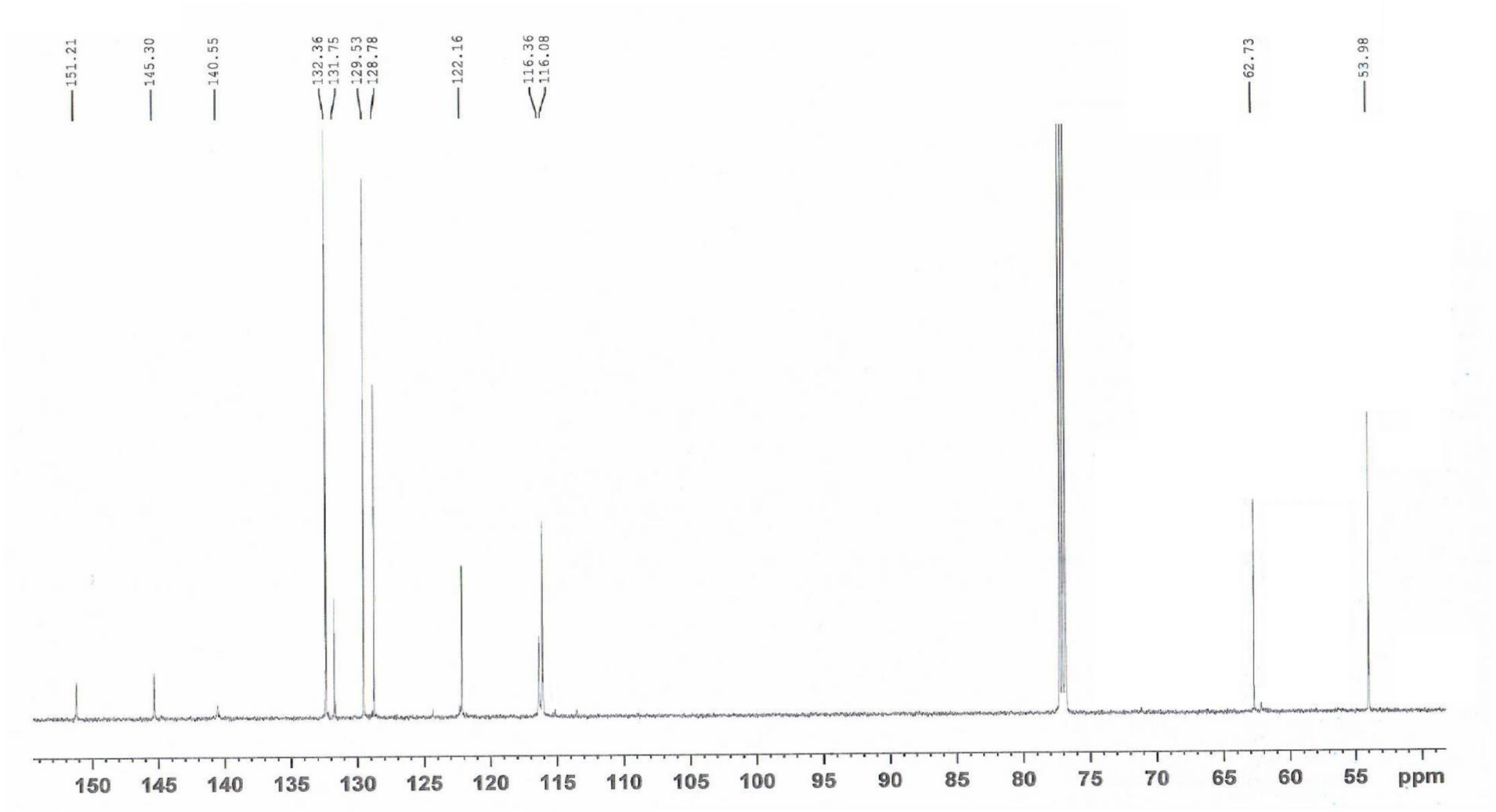

Fig. S30 ^{13}C NMR spectrum of of 1-(phenylthio)methyl-4-(4-aminophenoxy)methyl-1*H*-1,2,3-triazole **5l** in w $CDCl_3$

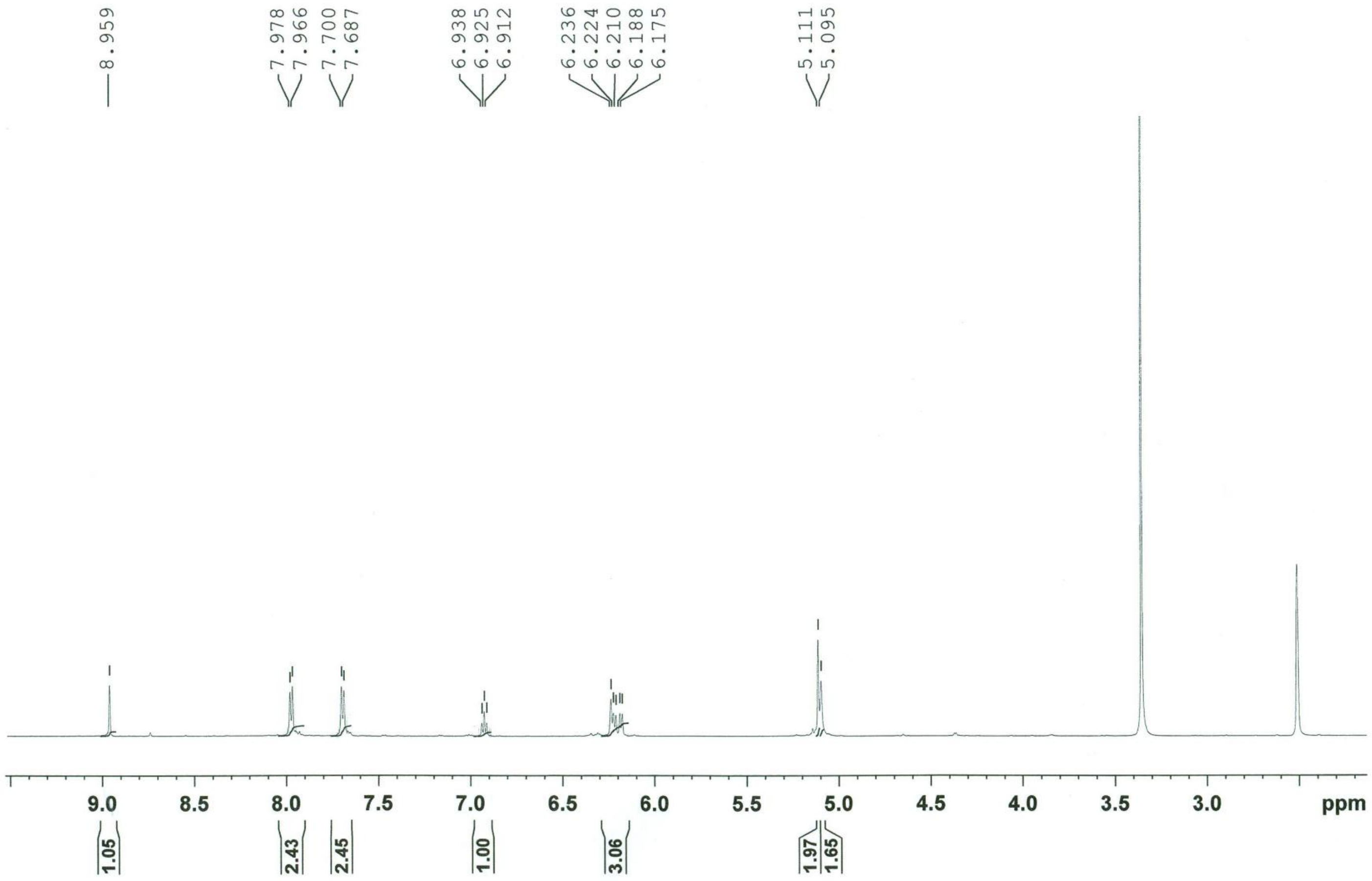

Fig S31 ^{1}H NMR spectrum of of 1-(-chlorophenyl)-4-(3-aminophenoxy)methyl-1*H*-1,2,3-triazole **5n** in DMSO

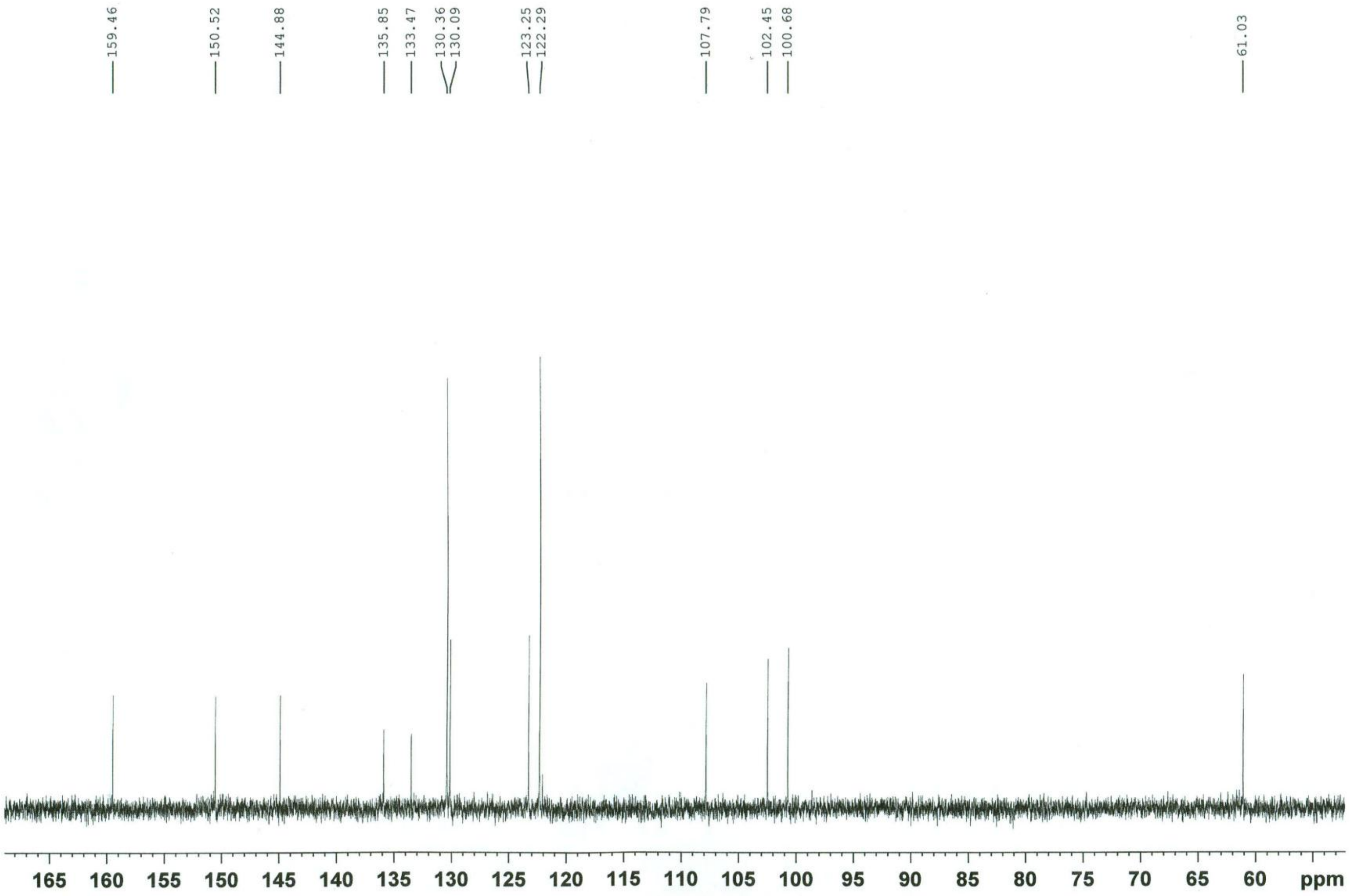

Fig. S32 ^{13}C NMR spectrum of 1-(4-chlorophenyl)-4-(3-aminophenoxy)methyl-1*H*-1,2,3-triazole **5m** in DMSO

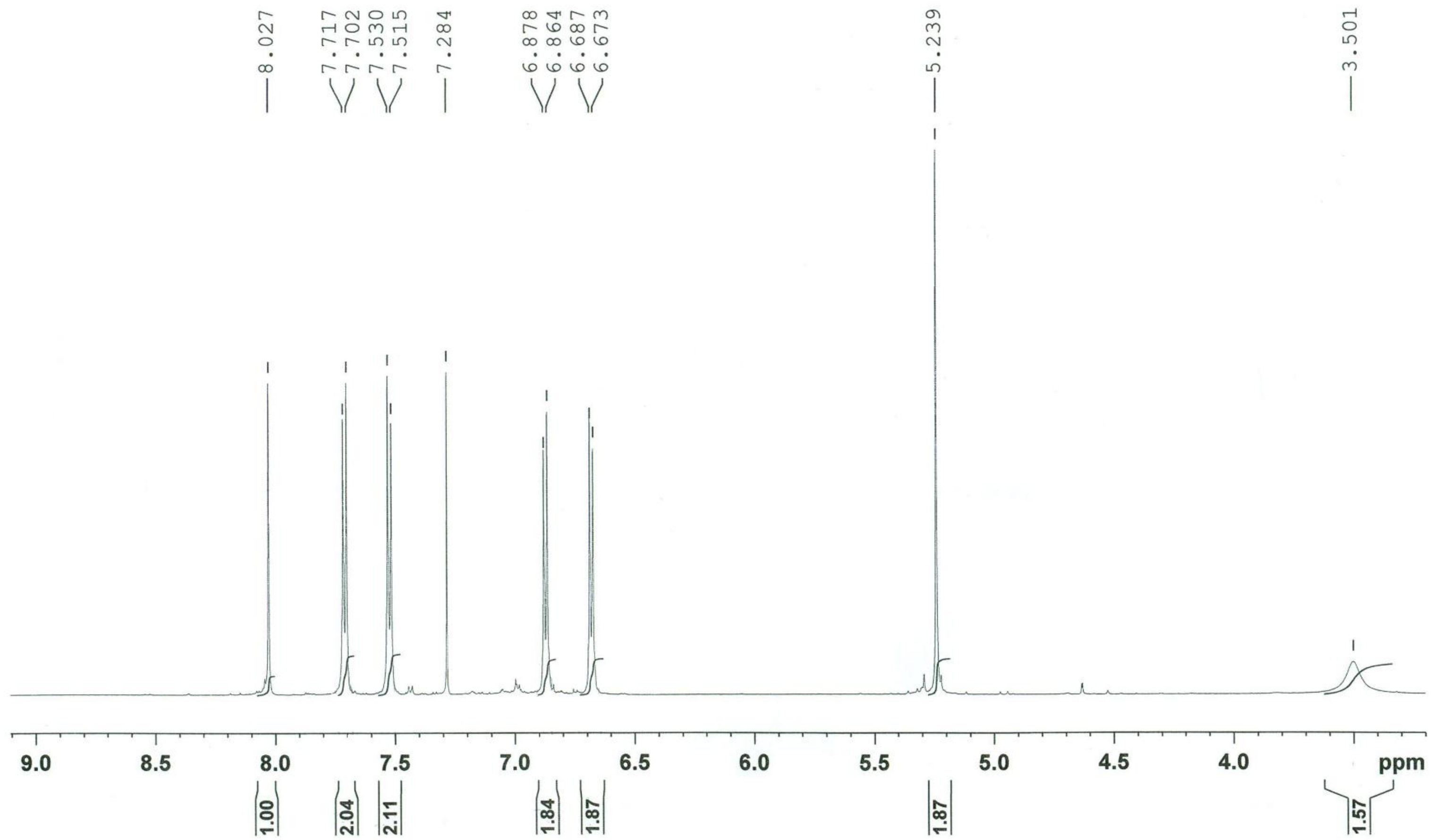

Fig. S33 ^{1}H NMR spectrum of 1-(4-chlorophenyl)-4-(4-aminophenoxy)methyl-1*H*-1,2,3-triazole **4n** in $CDCl_3$

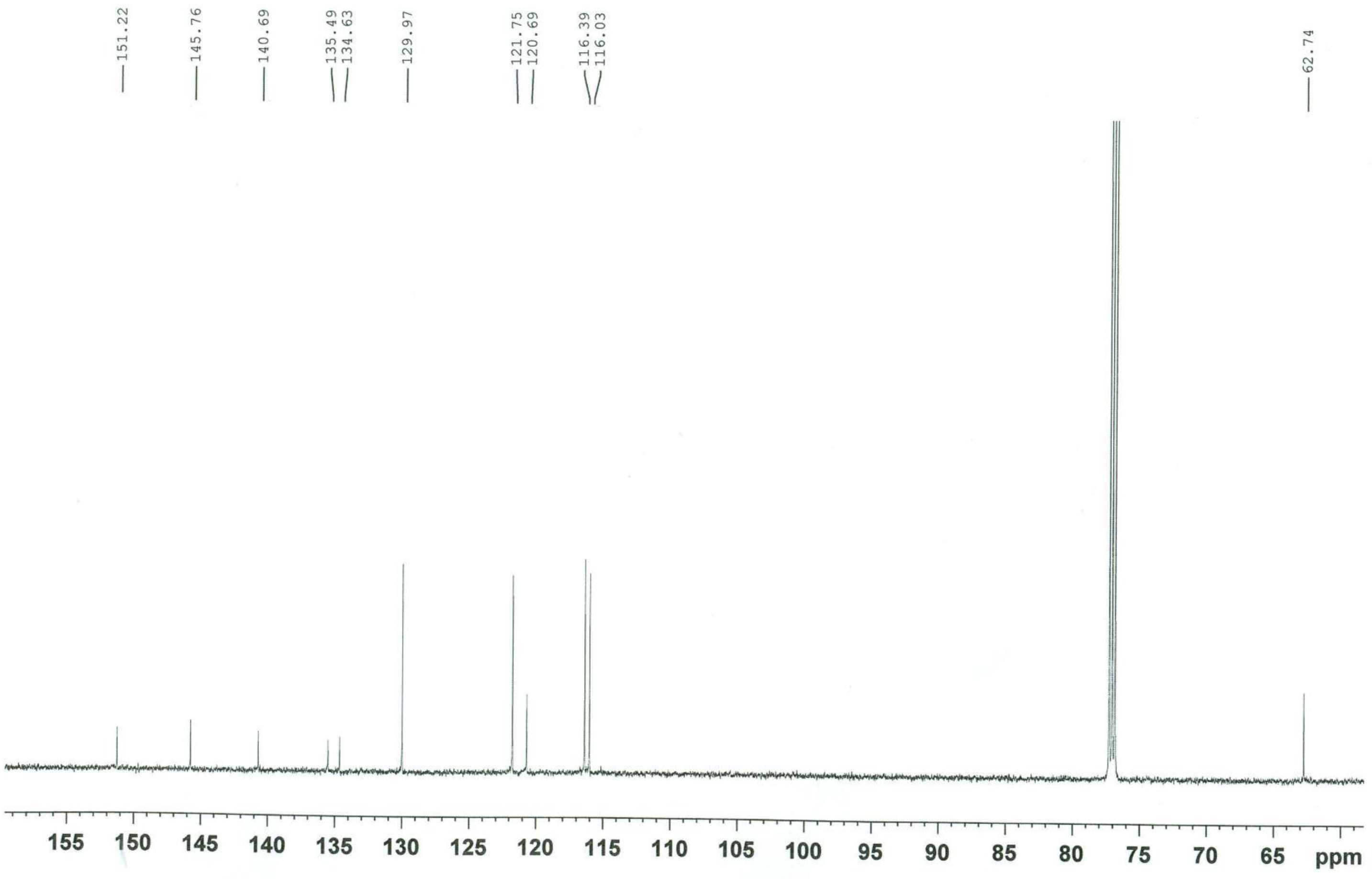

Fig. S34 ^{13}C NMR spectrum of 1-(4-chlorophenyl)-4-(4-aminophenoxy)methyl-1*H*-1,2,3-triazole **4n** in $CDCl_3$

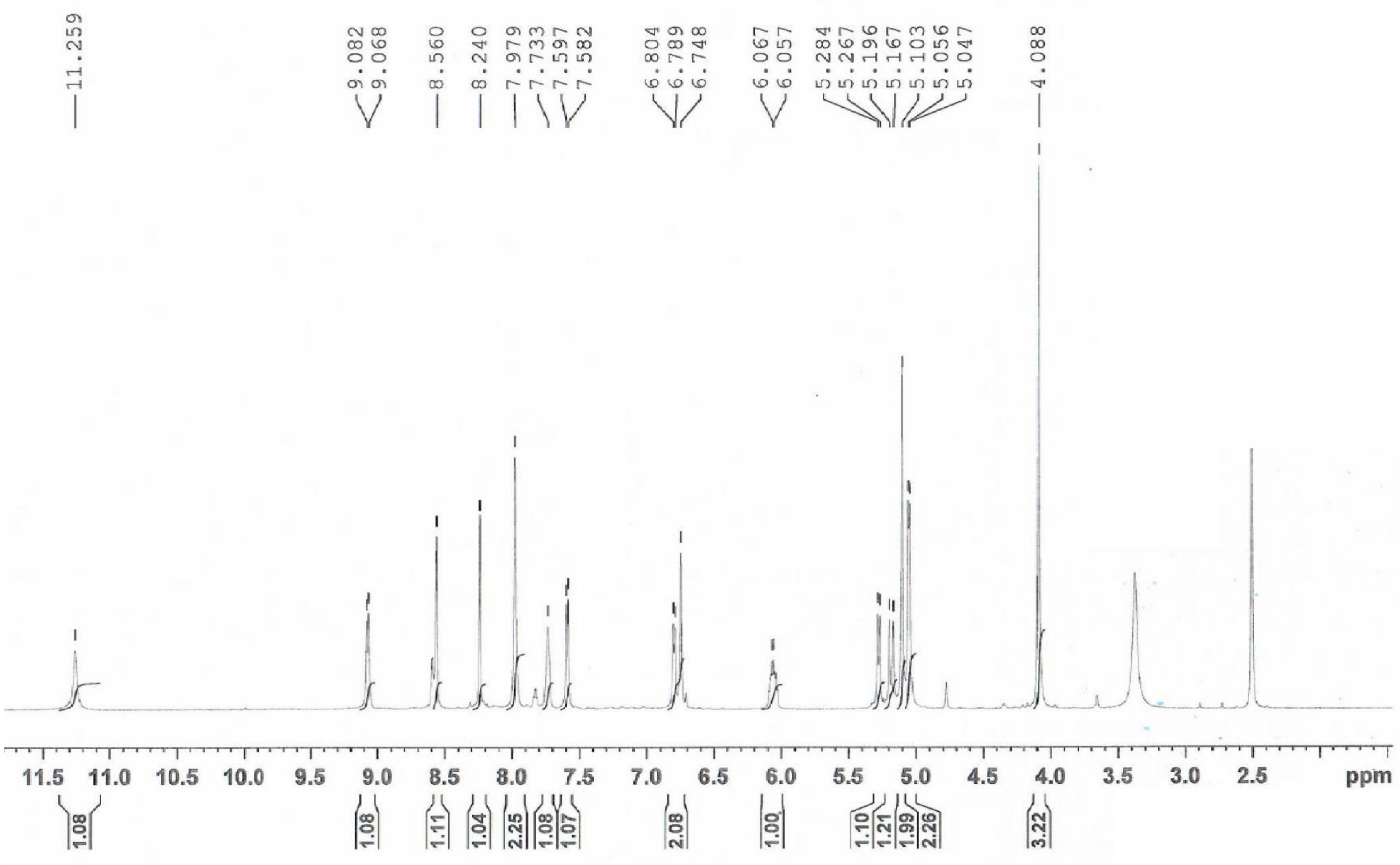

Fig. S35 ^{1}H NMR spectrum of 5-methyl-9-(1-allyl-1*H*-1,2,3-triazol-4-yl)methoxy-12*H*-quino[3,4-*b*][1,4]benzothiazinium chloride **2a** in DMSO

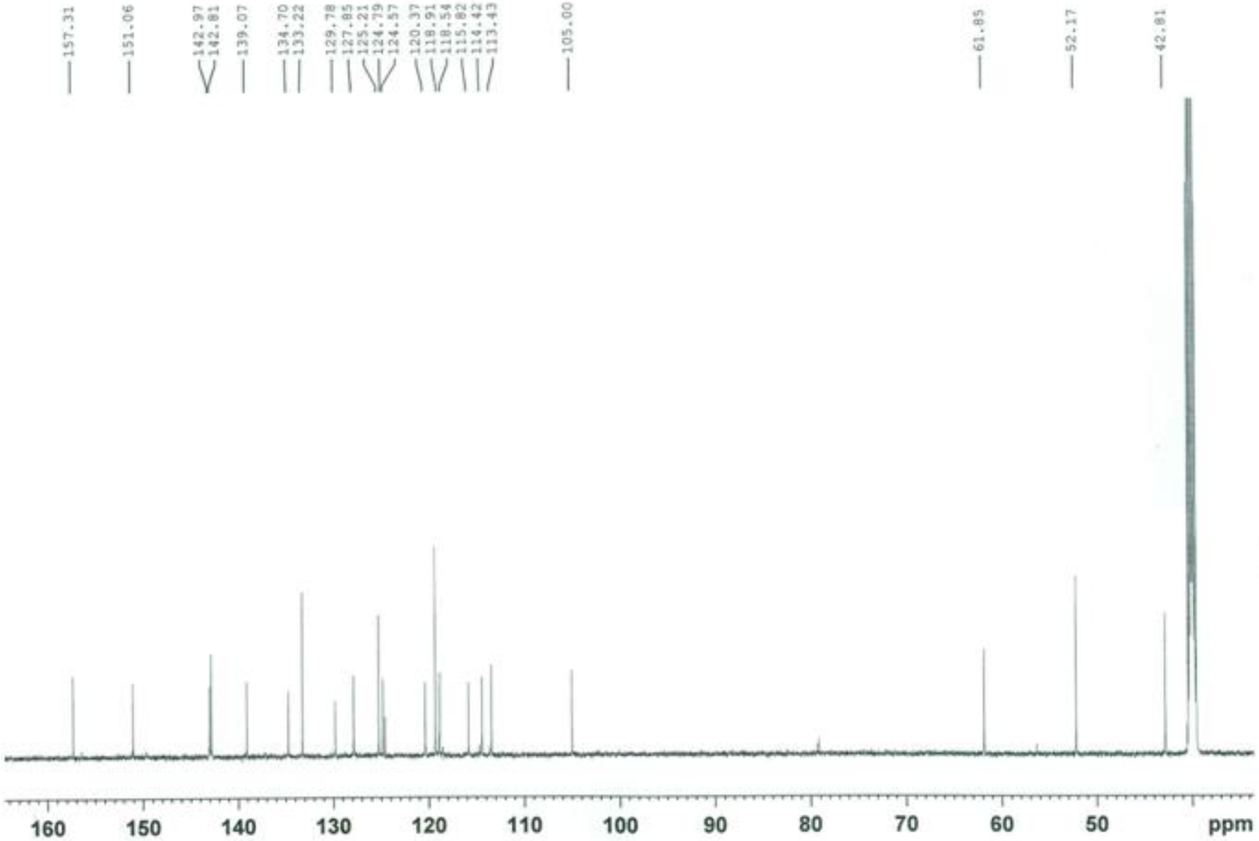

Fig. S36 ^{13}C NMR spectrum of 5-methyl-9-(1-allyl-1*H*-1,2,3-triazol-4-yl)methoxy-12*H*-quino[3,4-*b*][1,4]benzothiazinium chloride **2a** in DMSO

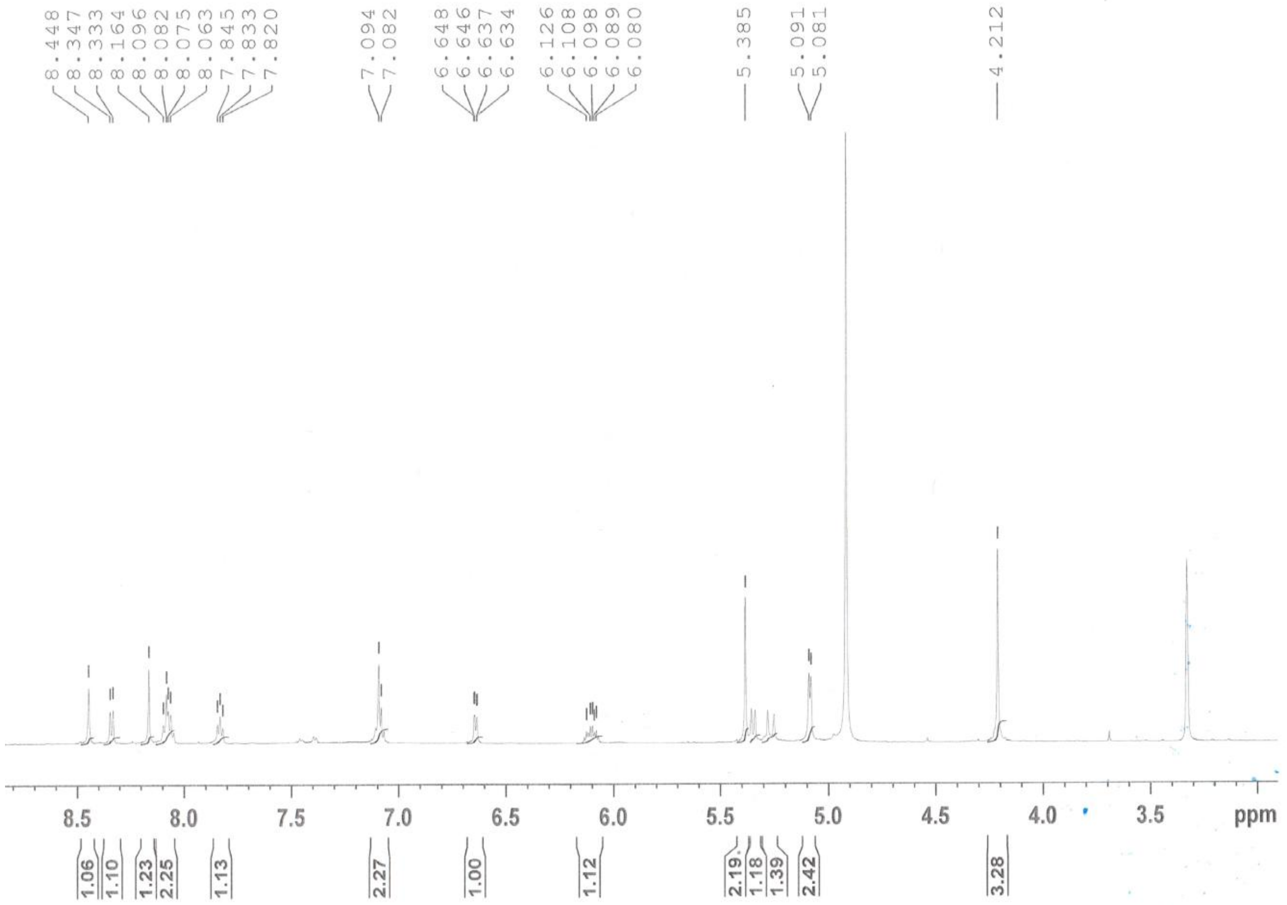

Fig. S37 ^{1}H NMR spectrum of 5-methyl-10-(1-allyl-1*H*-1,2,3-triazol-4-yl)methoxy-12*H*-quino[3,4-*b*][1,4]benzothiazinium chloride **2b** in CD_3OD

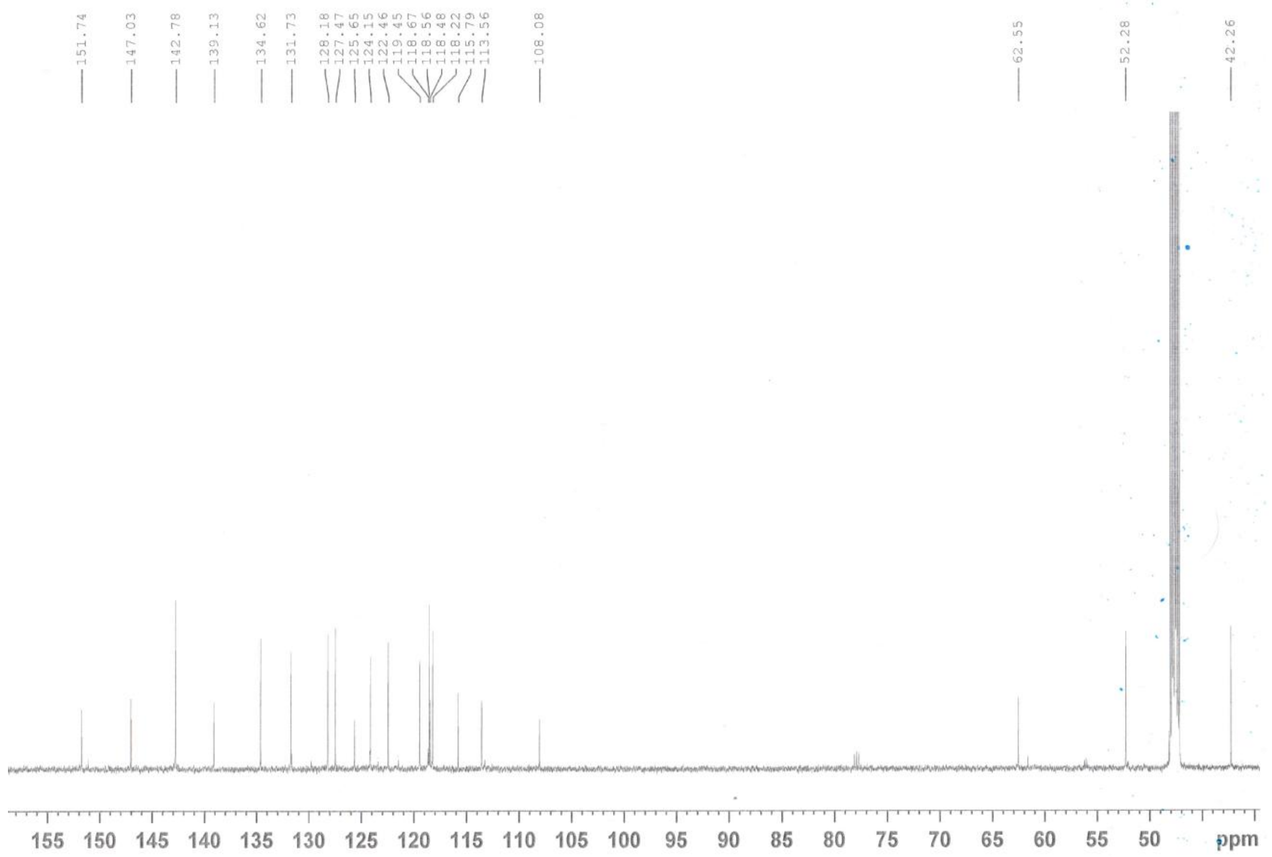

Fig. S38 ^{13}C NMR Spectrum of 5-methyl-10-(1-allyl-1*H*-1,2,3-triazol-4-yl)methoxy-12*H*-quino[3,4-*b*][1,4]benzothiazinium chloride **2b** in CD_3OD

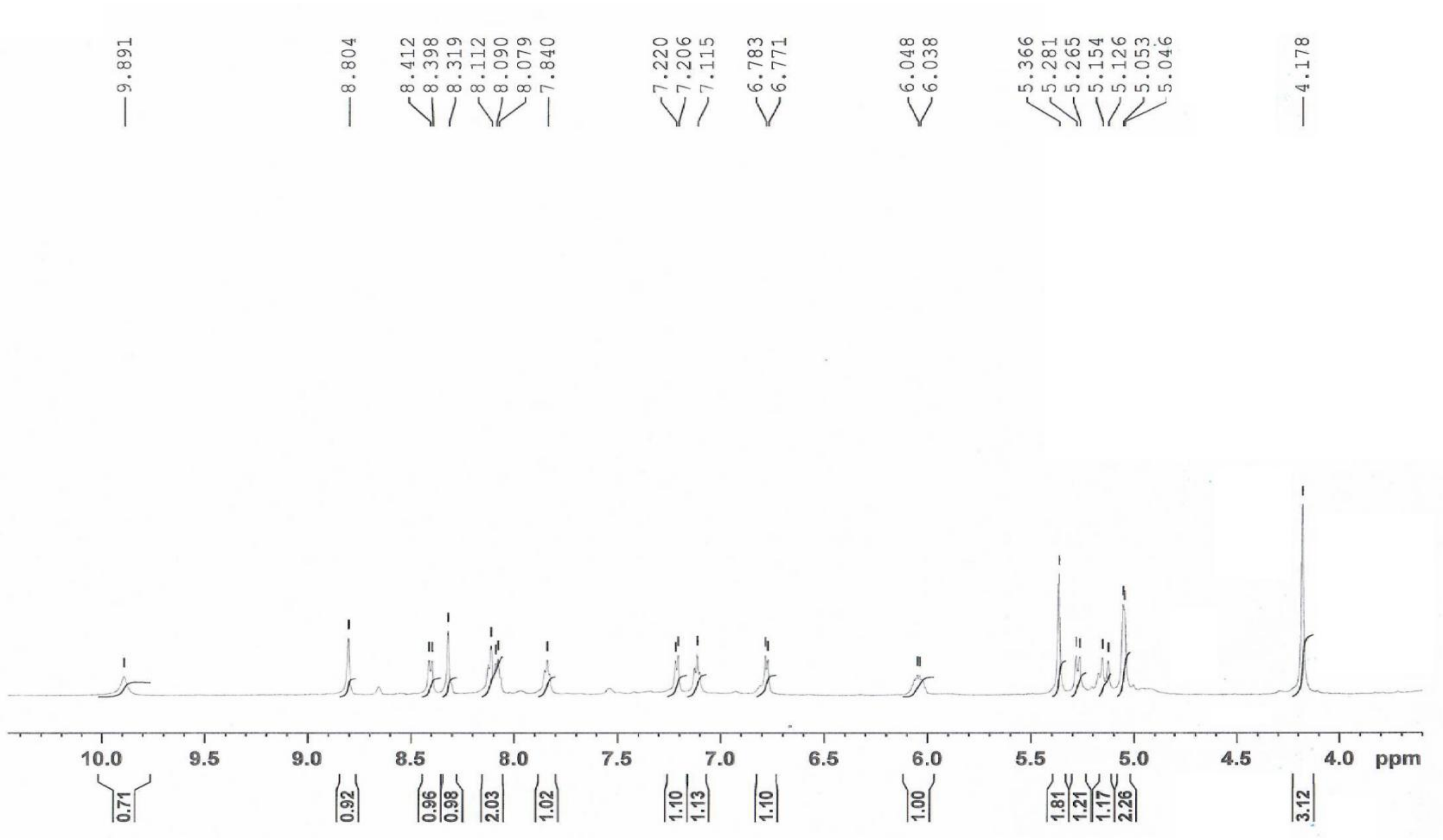

Fig. S39 ^{1}H NMR spectrum of 5-methyl-11-(1-allyl-1*H*-1,2,3-triazol-4-yl)methoxy-12*H*-quino[3,4-*b*][1,4]benzothiazinium chloride **2c** in DMSO

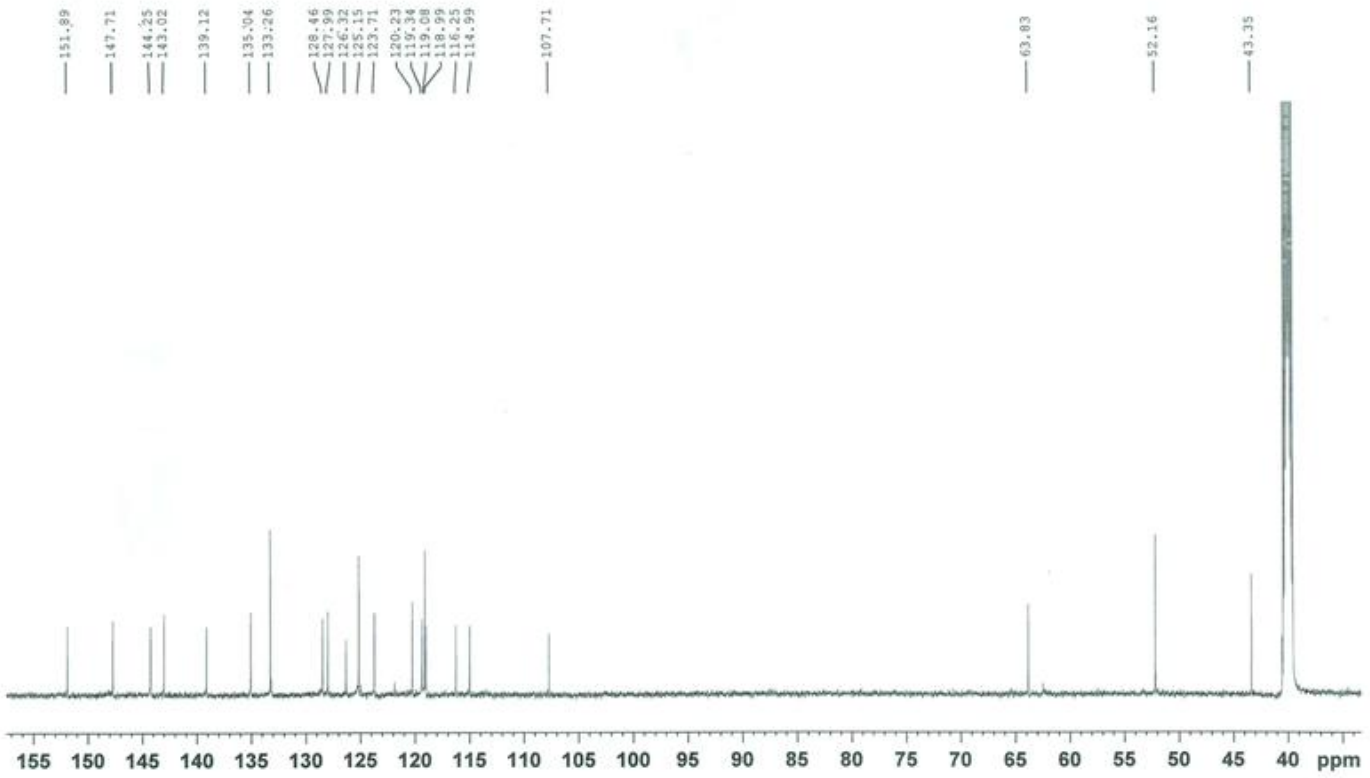

Fig. S40 ^{13}C NMR spectrum of 5-methyl-11-(1-allyl-1*H*-1,2,3-triazol-4-yl)methoxy-12*H*-quino[3,4-*b*][1,4]benzothiazinium chloride **2c** in DMSO

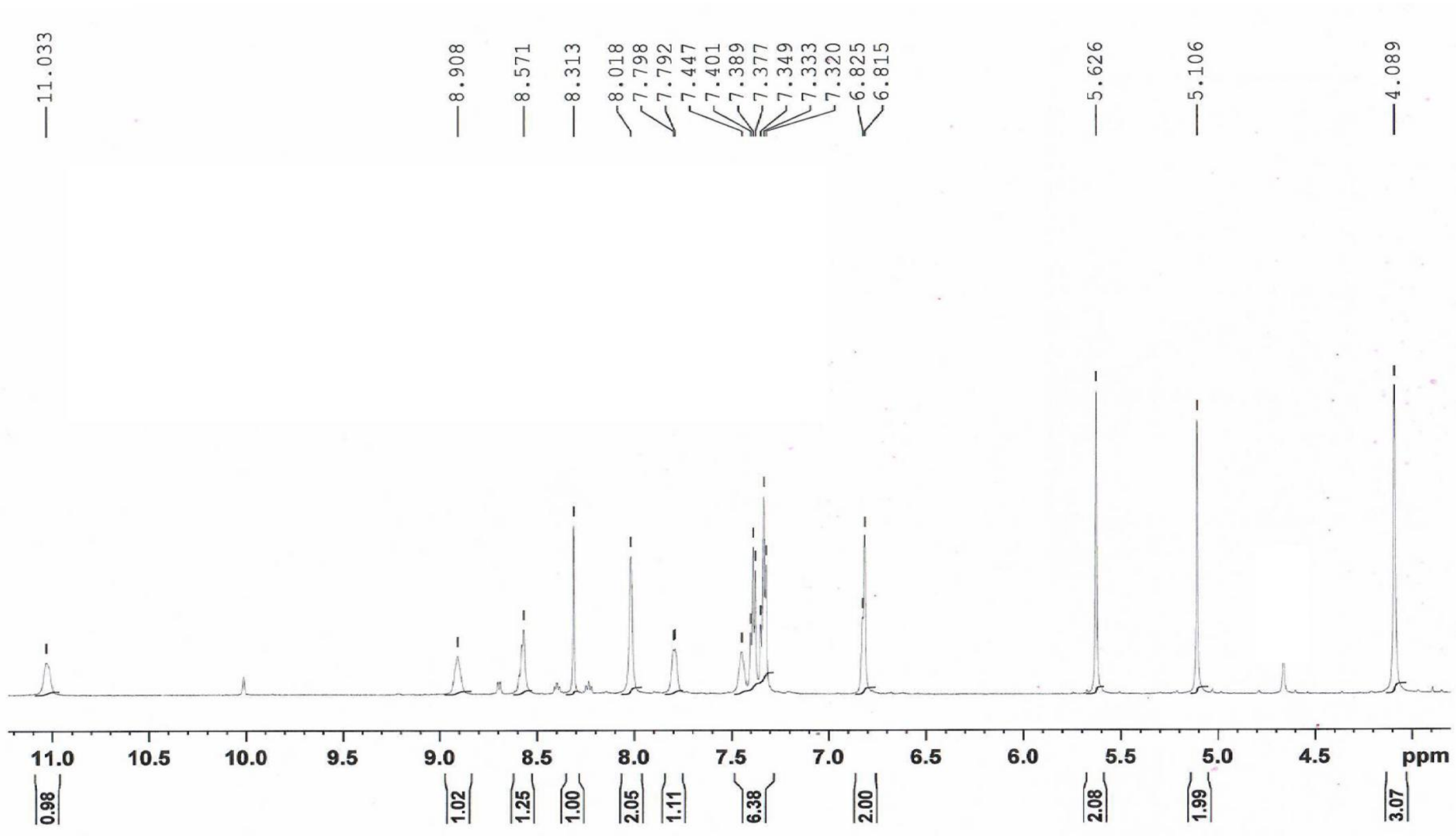

Fig. S41 ^{1}H NMR Spectrum of 5-Methyl-9-(1-benzyl-1*H*-1,2,3-triazol-4-yl)methoxy-12*H*-quino[3,4-*b*][1,4]benzothiazinium chloride **2d** in DMSO

.

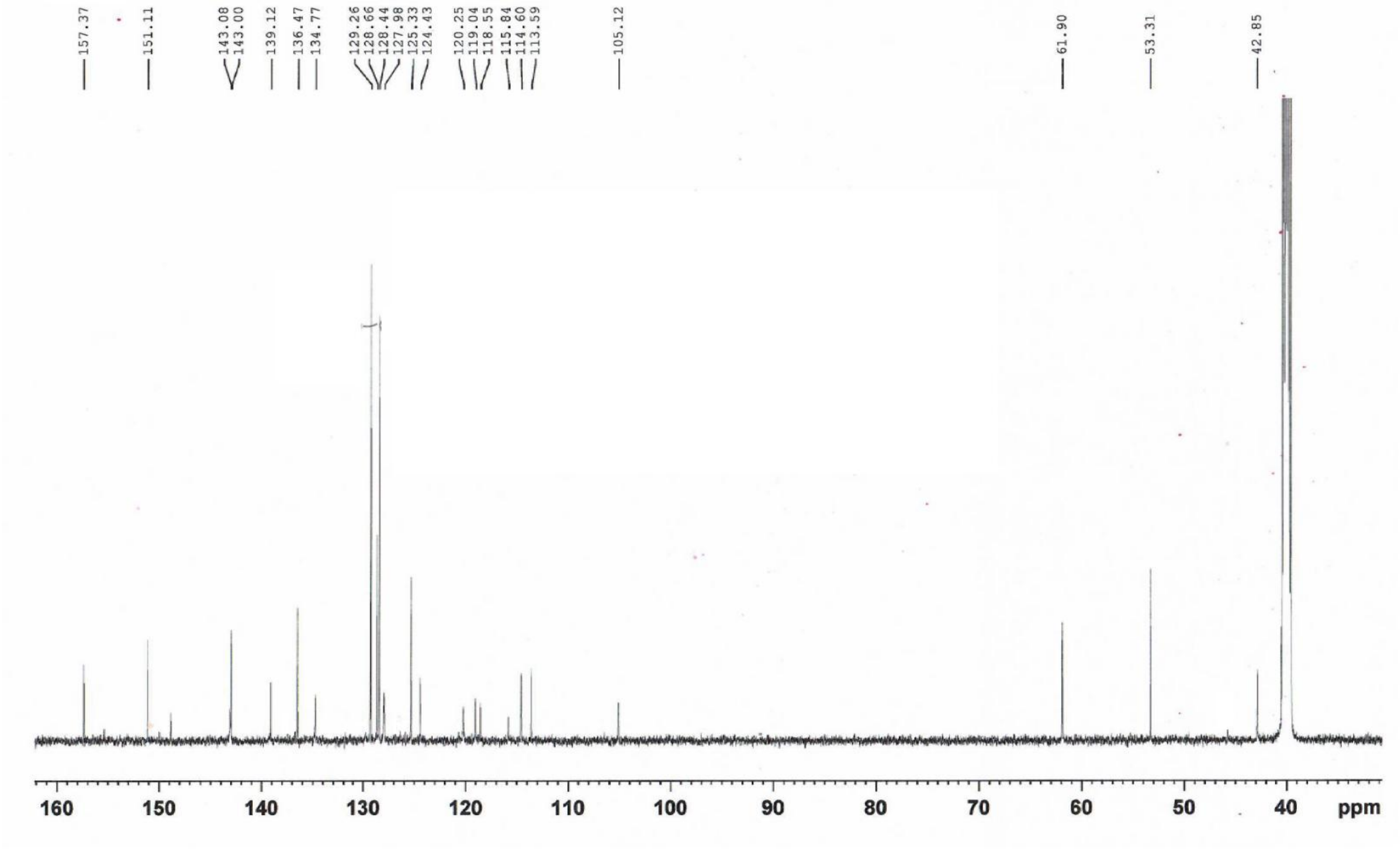

Fig. S42 ^{13}C NMR spectrum of 5-methyl-9-(1-benzyl-1*H*-1,2,3-triazol-4-yl)methoxy-12*H*-quino[3,4-*b*][1,4]benzothiazinium chloride **2d** in DMSO

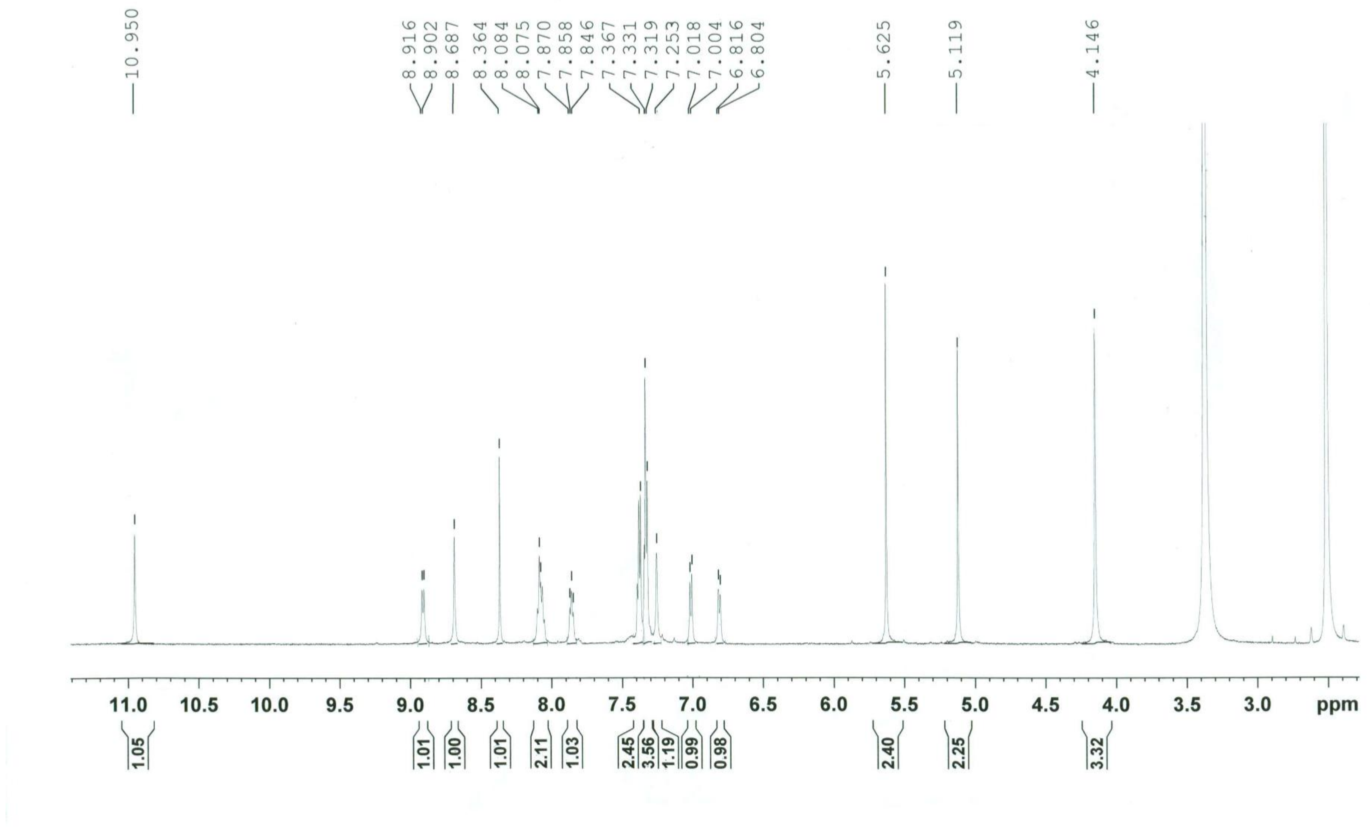

Fig. S43 ^{1}H NMR Spectrum of 5-Methyl-10-(benzyl-1H-1,2,3-triazol-4-yl)methoxy-12*H*-quino[3,4-*b*][1,4]benzothiazinium chloride **2e** in DMSO

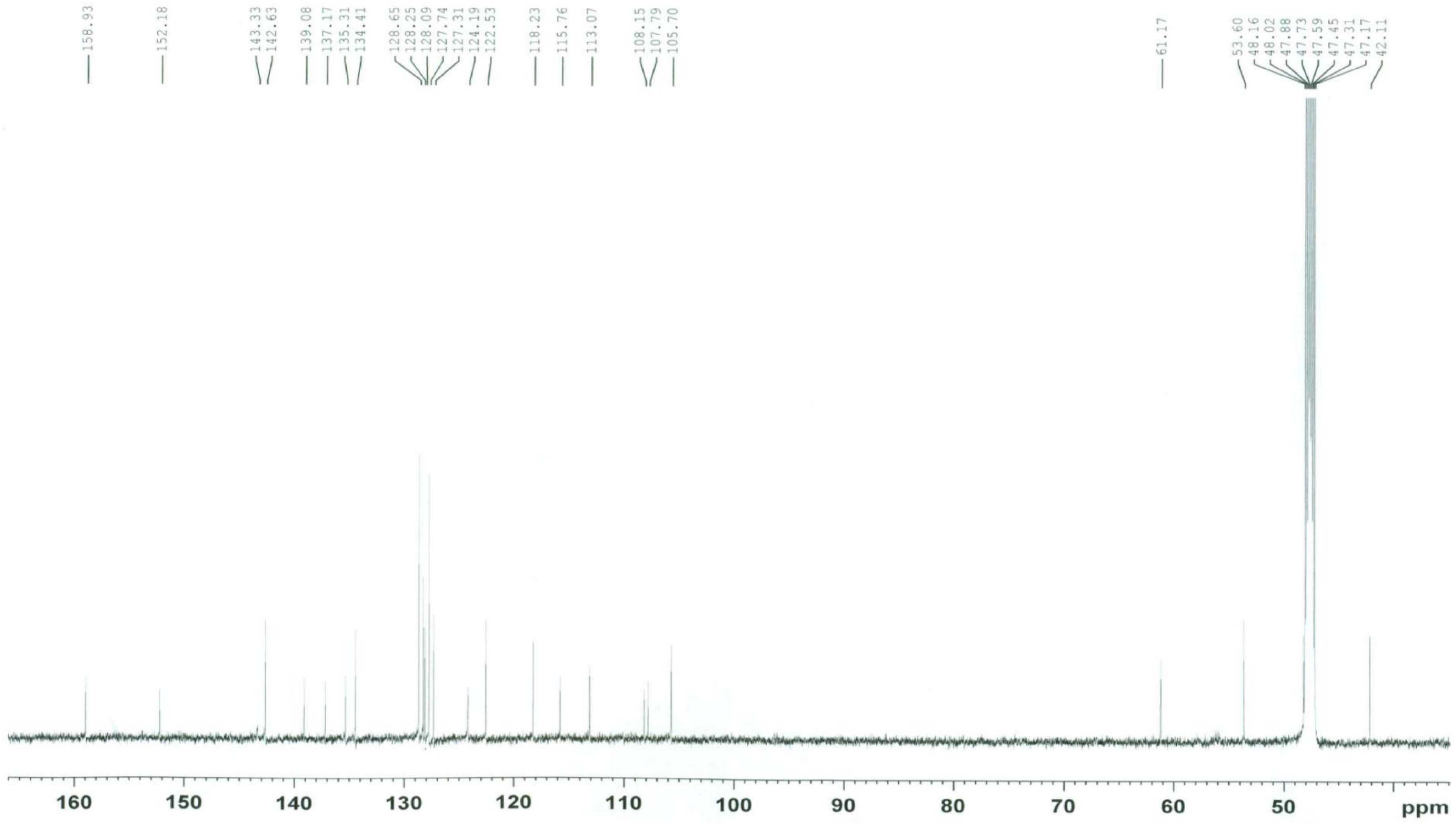

Fig. S44 ^{13}C NMR spectrum of 5-methyl-10-(1-benzyl-1*H*-1,2,3-triazol-4-yl)methoxy-12*H*-quino[3,4-*b*][1,4]benzothiazinium chloride **2e** in CD_3OD.

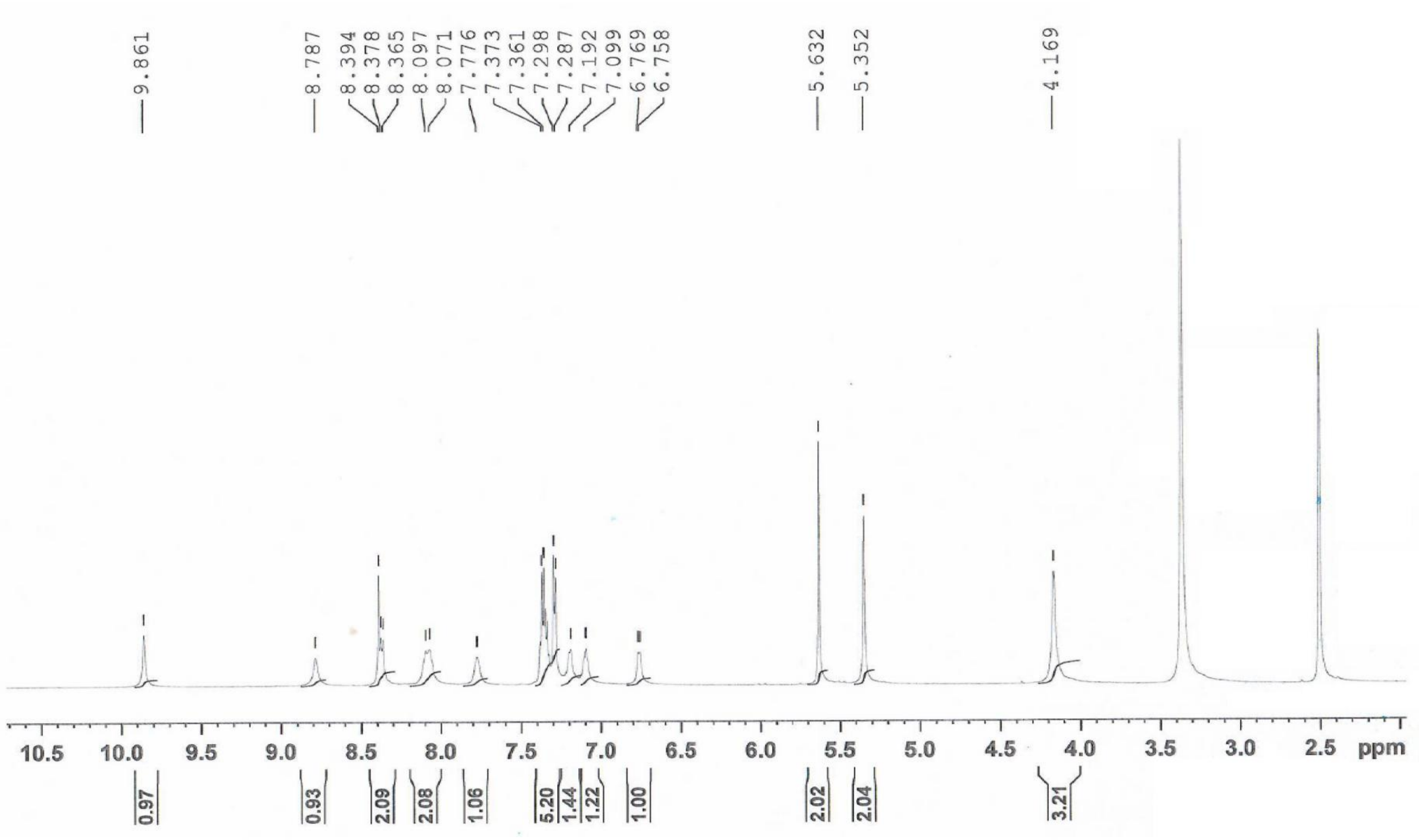

Fig. S45 ^{1}H NMR spectrum of 5-methyl-11-(1-benzyl-1*H*-1,2,3-triazol-4-yl)methoxy-12*H*-quino[3,4-*b*][1,4]benzothiazinium chloride **2f** in DMSO

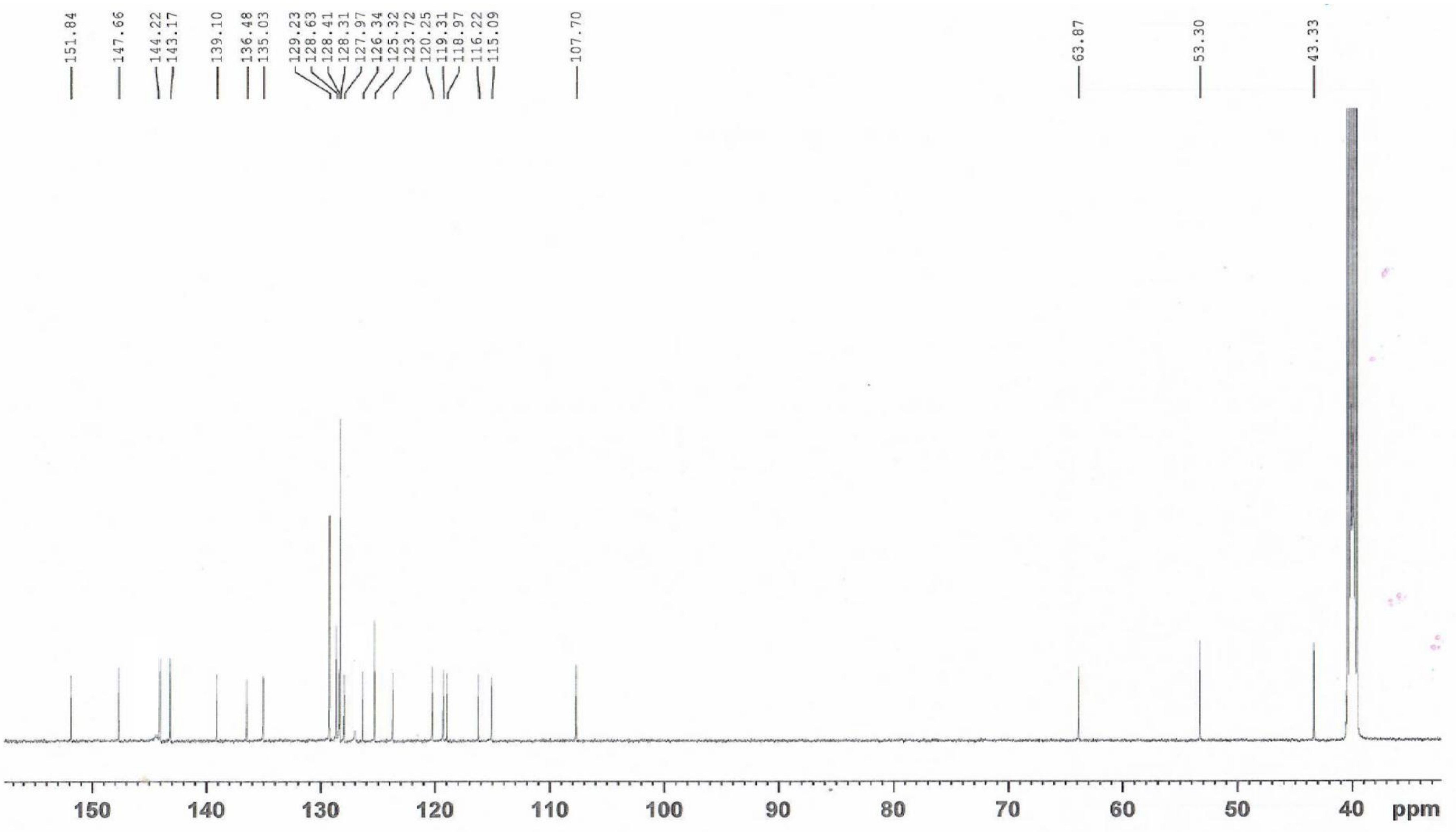

Fig. S46 ^{13}C NMR spectrum of 5-methyl-11-(1-benzyl-1*H*-1,2,3-triazol-4-yl)methoxy-12*H*-quino[3,4-*b*][1,4]benzothiazinium chloride **2f** in DMSO

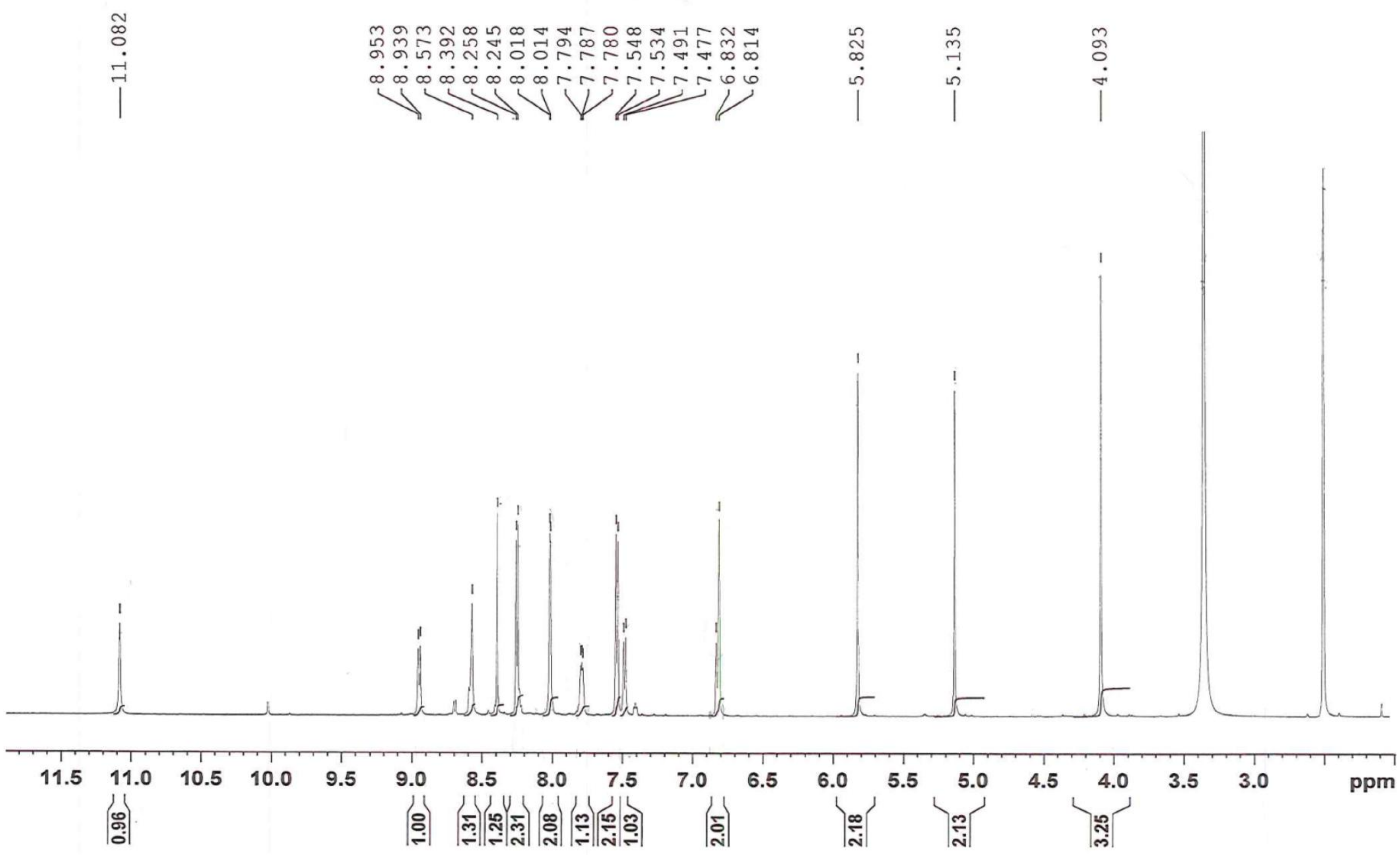

Fig. S47 ^{1}H NMR Spectrum of 5-methyl-9-[1-(4-nitrobenzyl)-1*H*-1,2,3-triazol-4-yl]methoxy-12*H*-quino[3,4-*b*][1,4]benzothiazinium chloride **2g** in DMSO

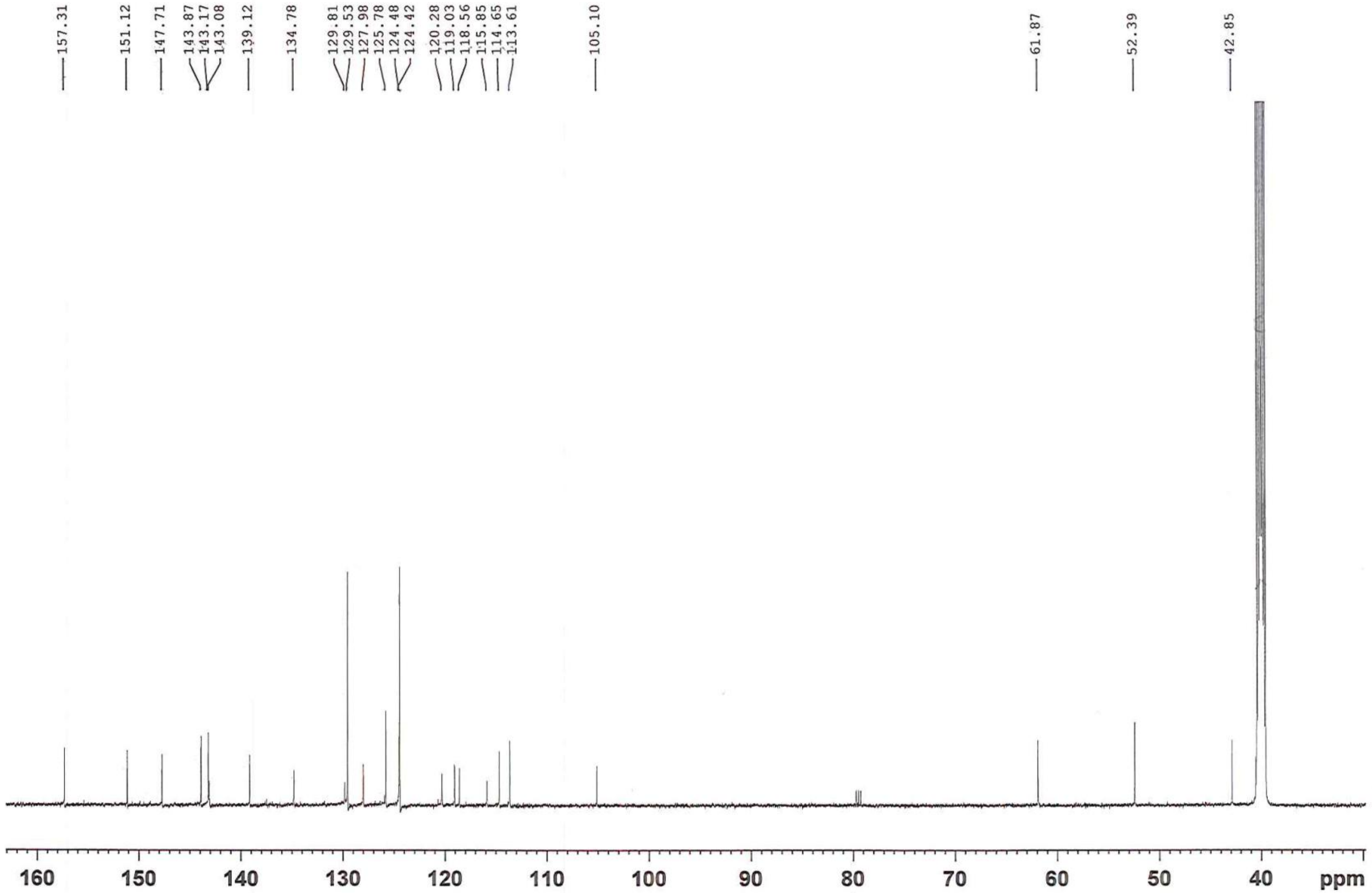

Fig. S48 ^{13}C NMR spectrum of 5-methyl-9-[1-(4-nitrobenzyl)-1*H*-1,2,3-triazol-4-yl]methoxy-12*H*-quino[3,4-*b*][1,4]benzothiazinium chloride **2g** in DMSO

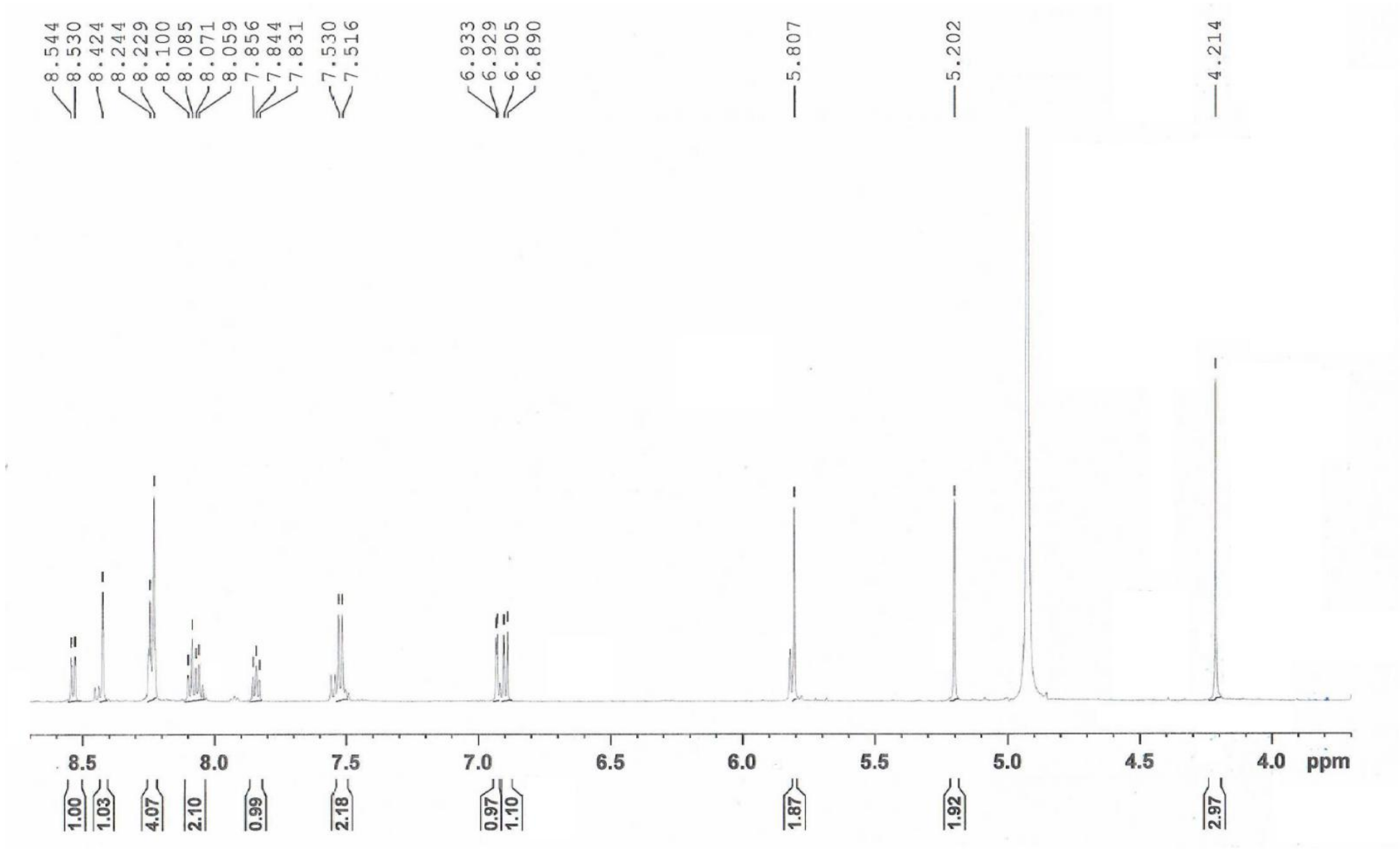

Fig. S49 ^{1}H NMR Spectrum of 5-Methyl-10-[1-(4-nitrobenzyl)-1*H*-1,2,3-triazol-4-yl]methoxy-12*H*-quino[3,4-*b*][1,4]benzothiazinium chloride **2h** in CD_3OD

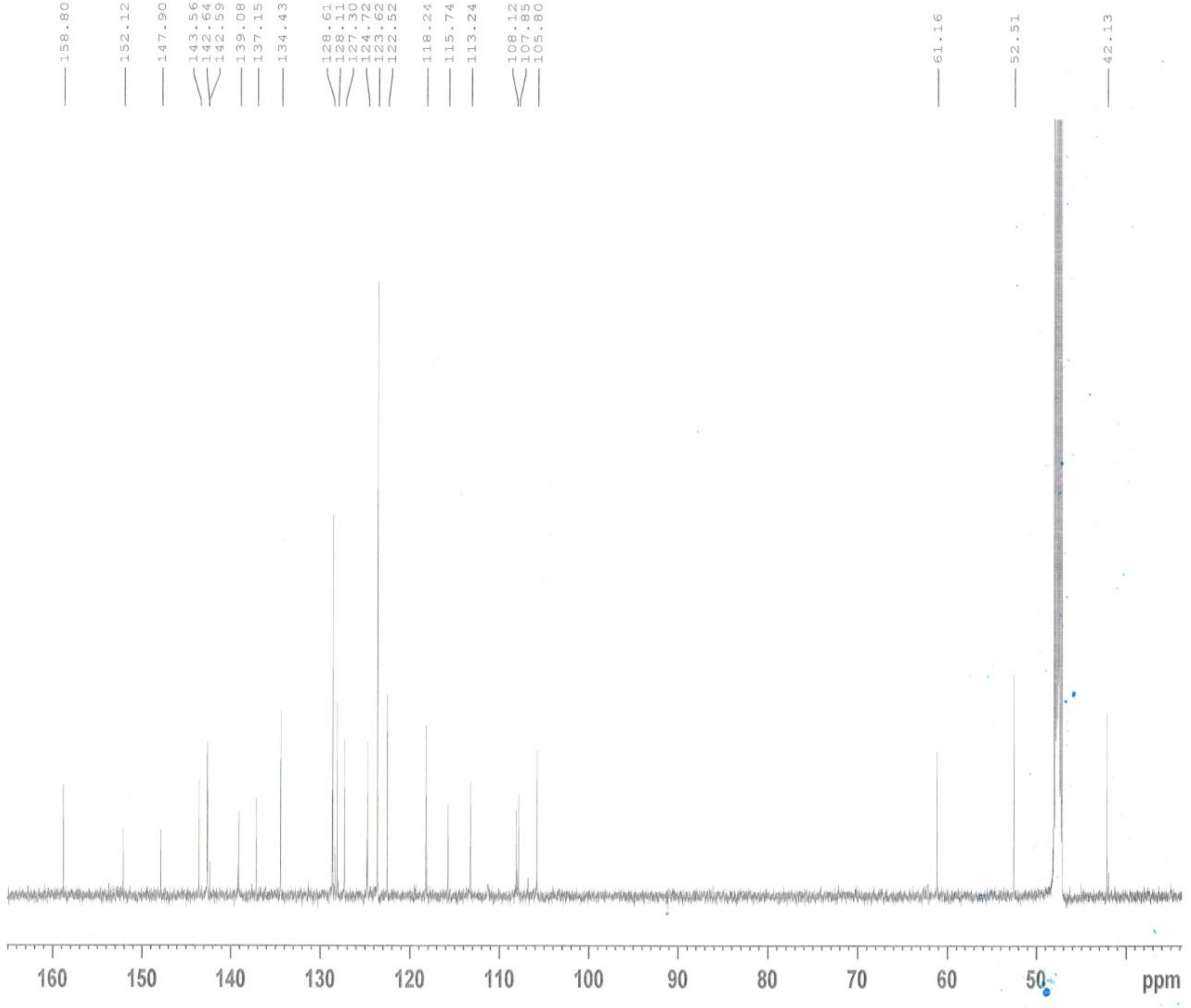

Fig. S50 ^{13}C NMR spectrum of 5-methyl-10-[1-(4-nitrobenzyl)-1*H*-1,2,3-triazol-4-yl]methoxy-12*H*-quino[3,4-*b*][1,4]benzothiazinium chloride **2h** in CD_3OD

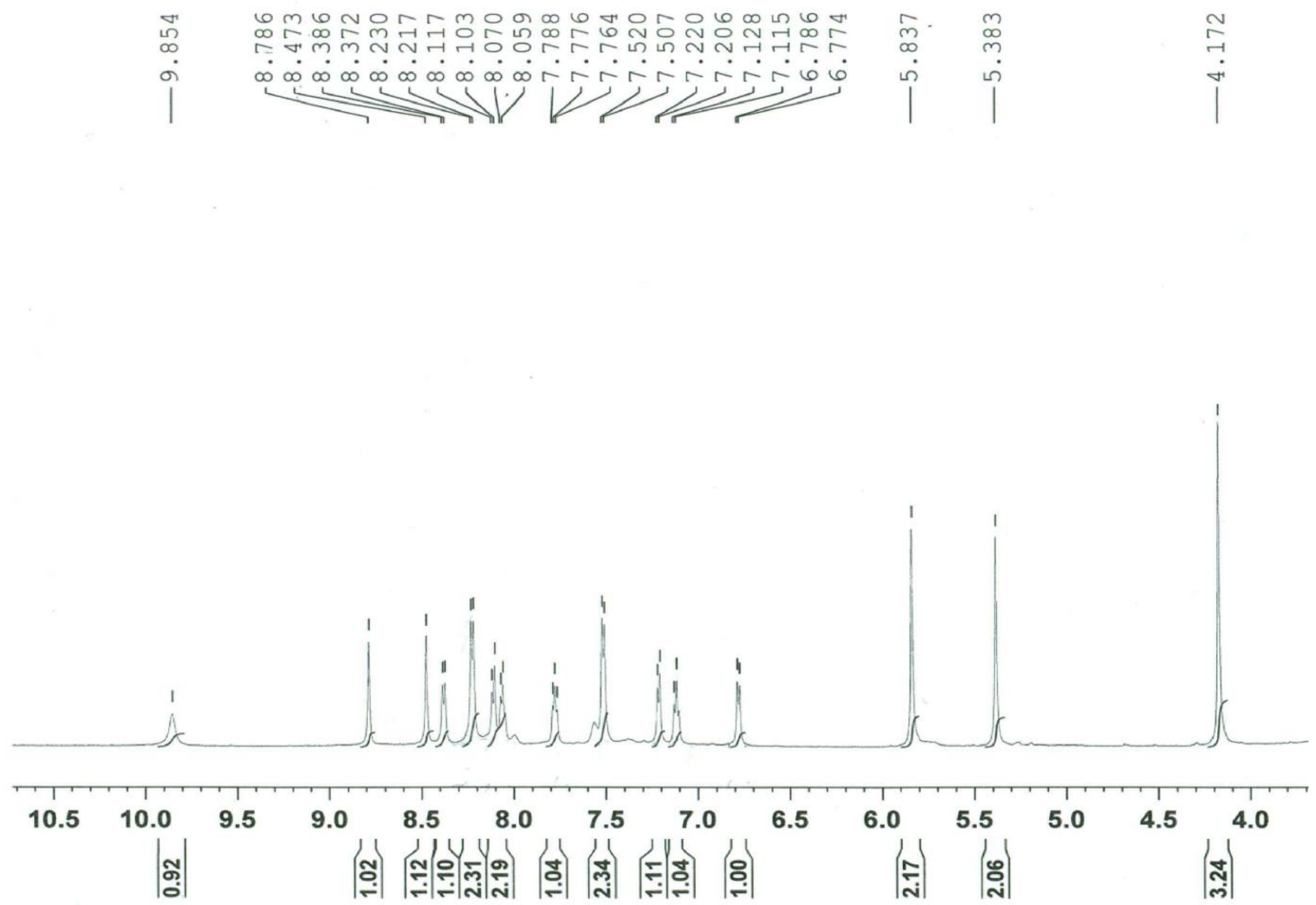

Fig. S51 ^{1}H NMR spectrum of 5-methyl-11-[1-(4-nitrobenzyl)-1*H*-1,2,3-triazol-4-yl]methoxy-12*H*-quino[3,4-*b*][1,4]benzothiazinium chloride **2i** in DMSO

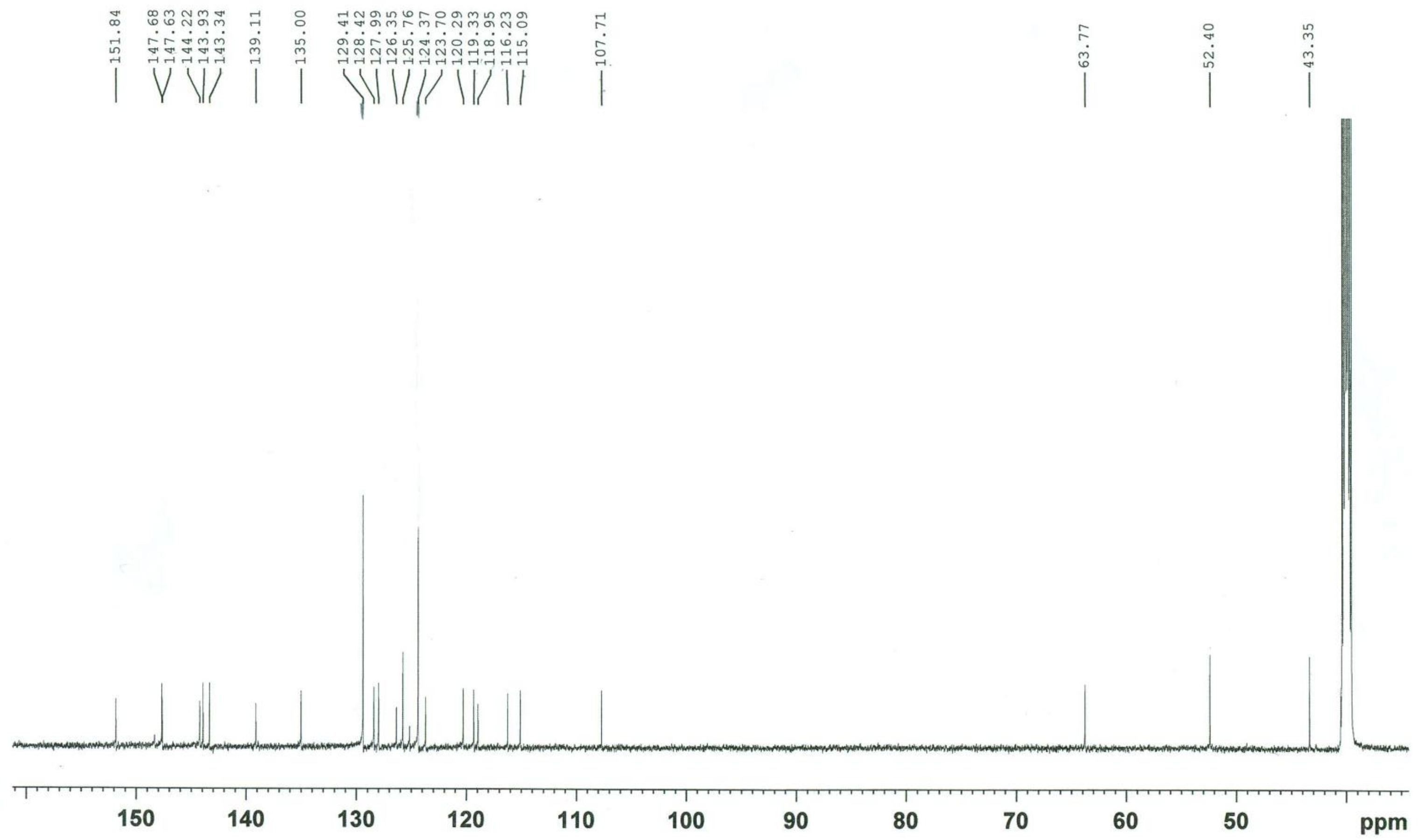

Fig. S52 ^{13}C NMR spectrum of 5-methyl-11-[1-(4-nitrobenzyl)-1*H*-1,2,3-triazol-4-yl]methoxy-12*H*-quino[3,4-*b*][1,4]benzothiazinium chloride **2i** in DMSO

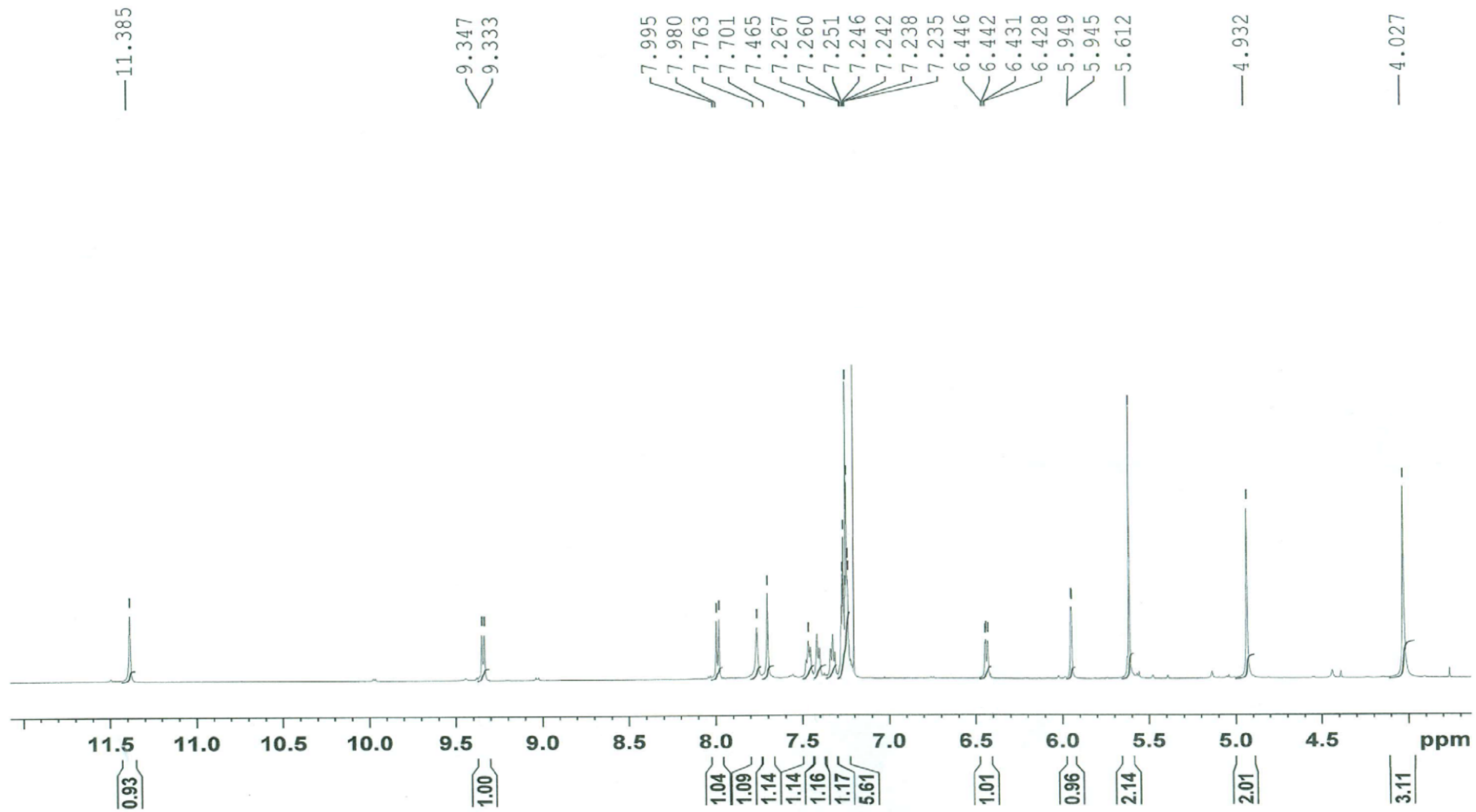

Fig. S53 ^{1}H NMR spectrum 5-methyl-9-(1-(phenylthio)methyl-1*H*-1,2,3-triazol-4-yl)methoxy-12*H*-quino[3,4-*b]*[1,4]benzothiazinium chloride **2j** in DMSO

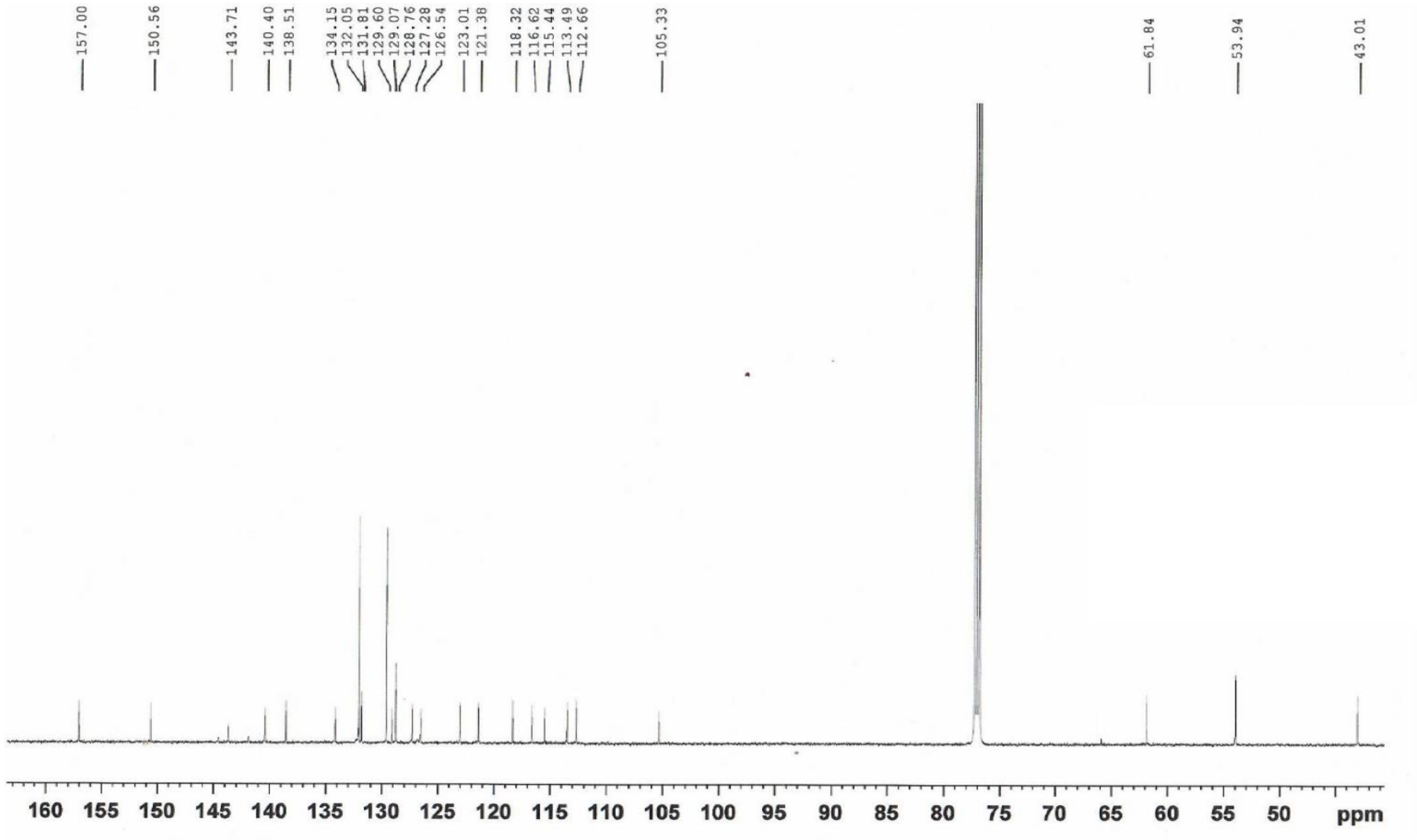

Fig. S54 ^{13}C NMR Spectrum 5-Methyl-9-(1-(phenylthio)methyl-1*H*-1,2,3-triazol-4-yl)methoxy-12*H*-quino[3,4-*b*][1,4]benzothiazinium chloride **2j** in DMSO

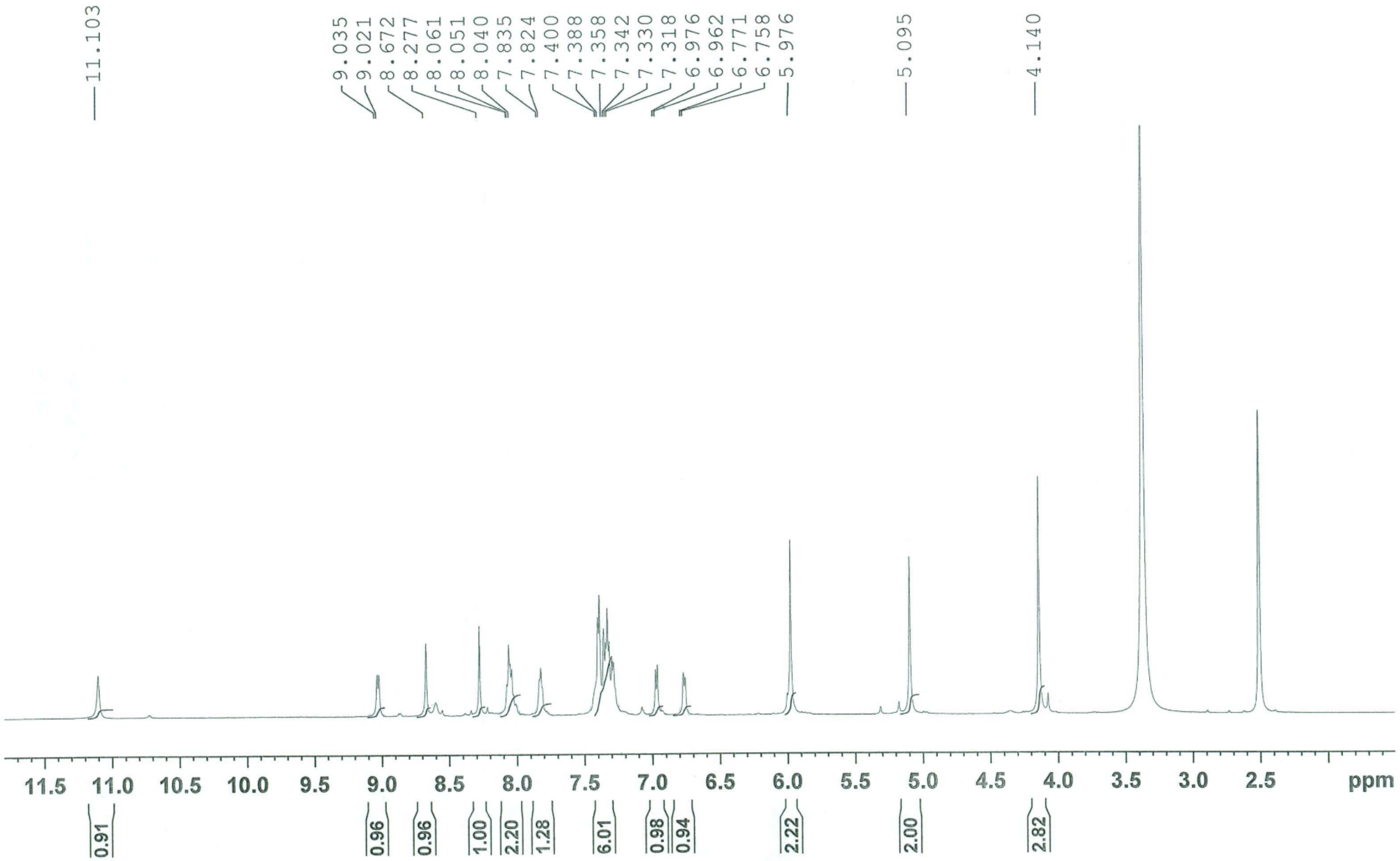

Fig. S55 ^{1}H NMR Spectrum 5-Methyl-10-(1-(phenylthio)methyl-1*H*-1,2,3-triazol-4-yl)methoxy-12*H*-quino[3,4-*b]*[1,4]benzothiazinium chloride**2k** in DMSO

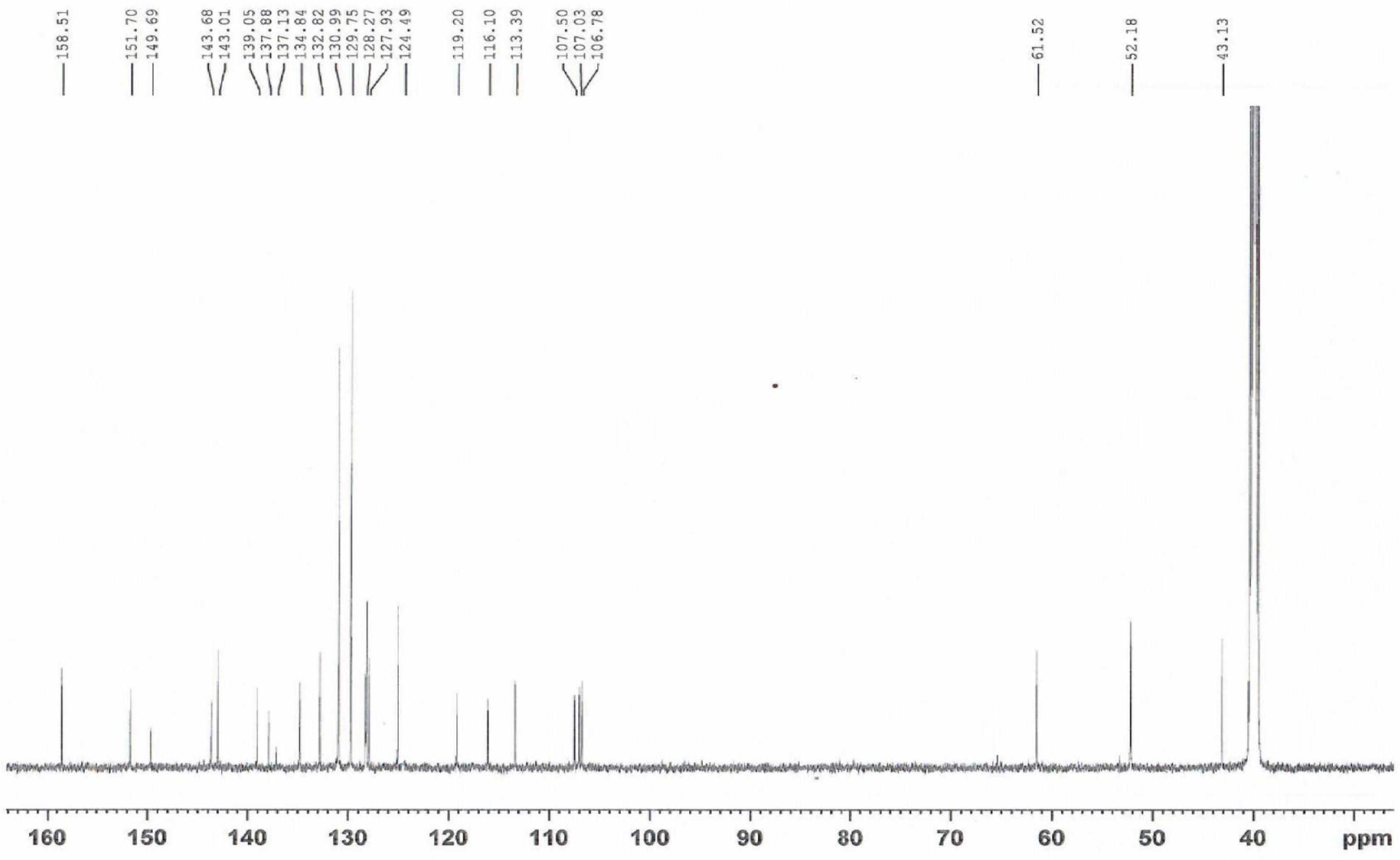

Fig. S56 ^{13}C NMR Spectrum 5-Methyl-10-(1-(phenylthio)methyl-1*H*-1,2,3-triazol-4-yl)methoxy-12*H*-quino[3,4-*b]*[1,4]benzothiazinium chloride **2k** in DMSO

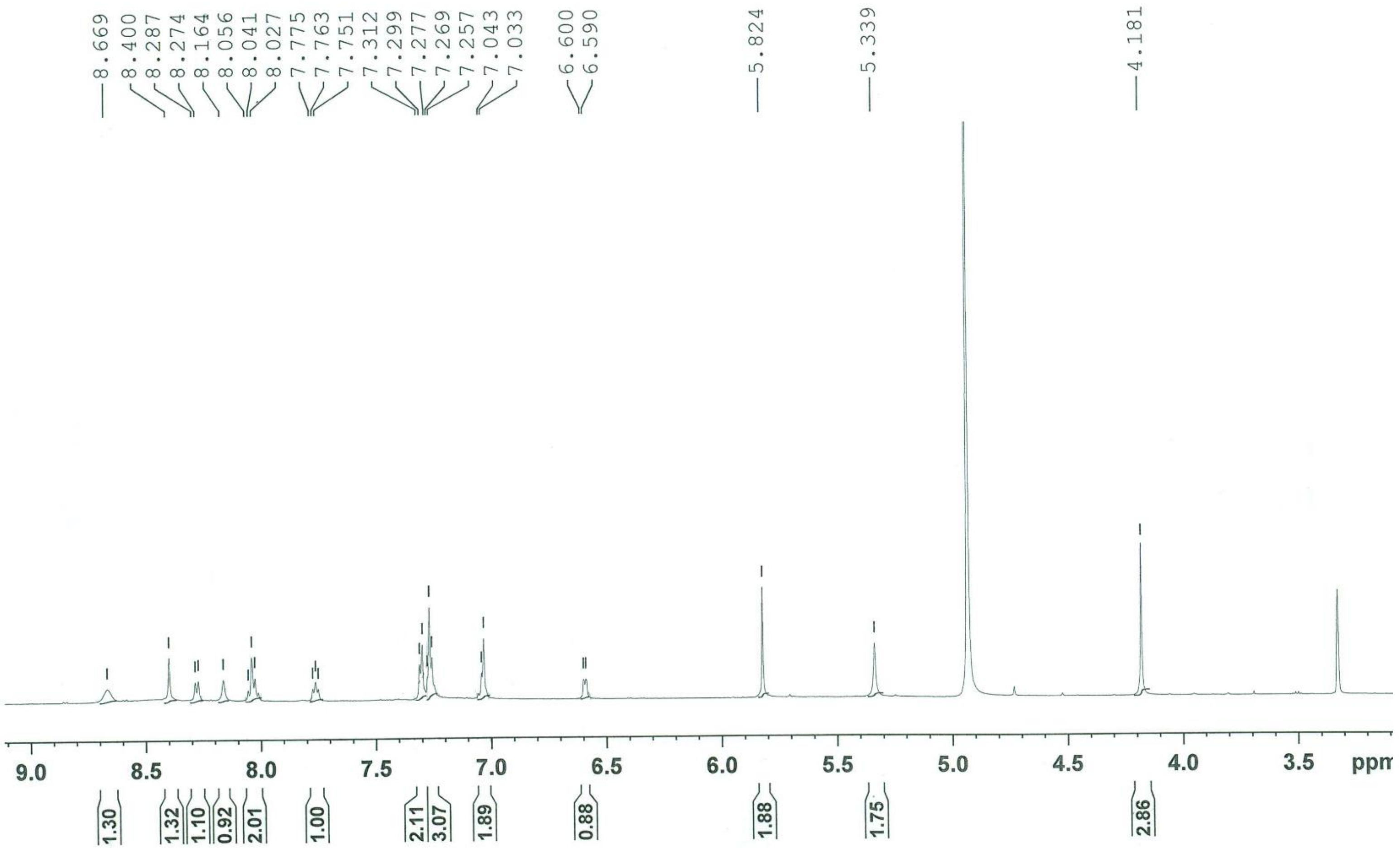

Fig. S57 ^{1}H NMR Spectrum 5-methyl-11-(1-(phenylthio)methyl-1*H*-1,2,3-triazol-4-yl)methoxy-12*H*-quino[3,4-*b*][1,4]benzothiazinium chloride **2l** in DMSO

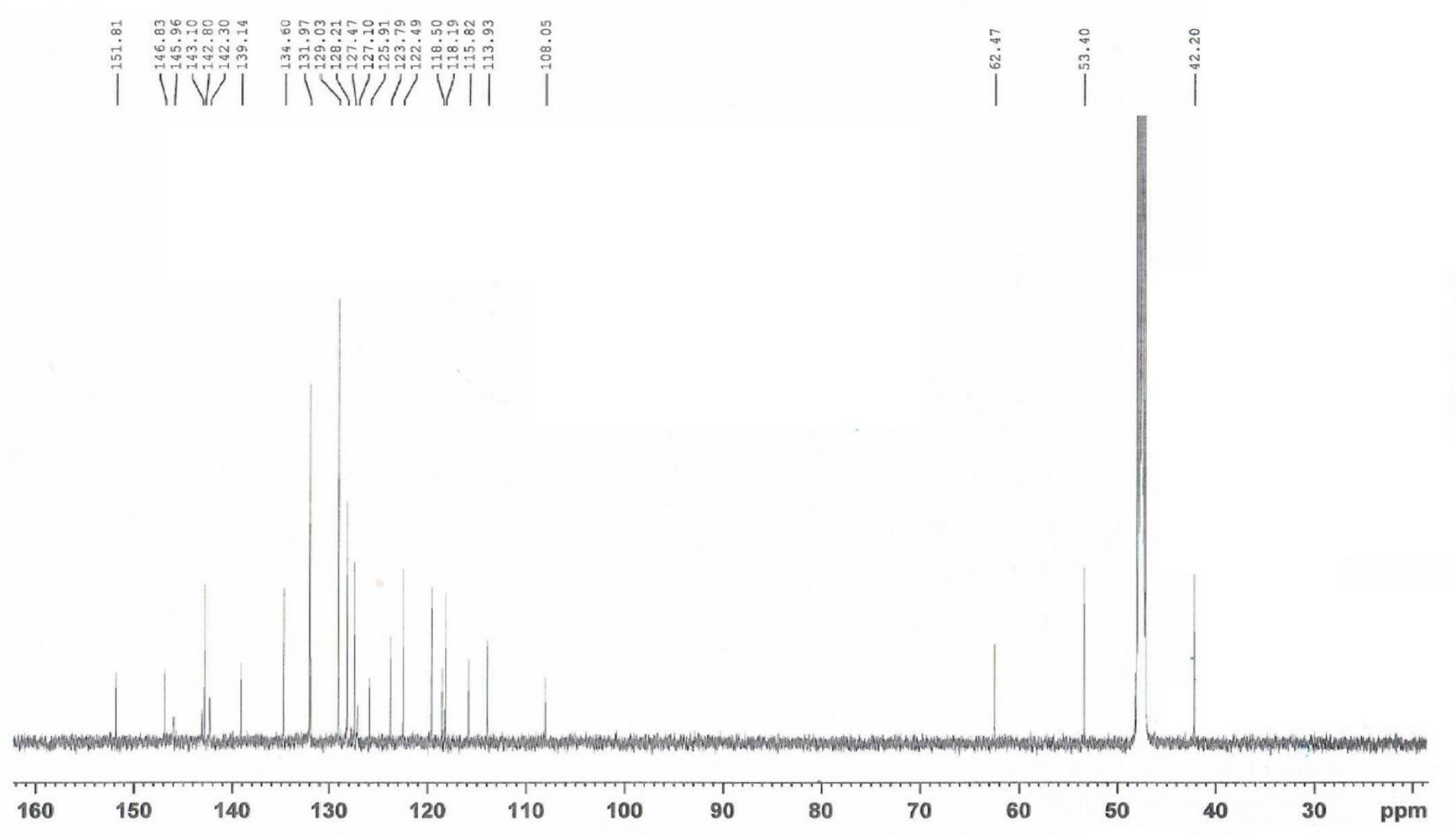

Fig. S58 ^{1}H NMR Spectrum 5-Methyl-11-(1-(phenylthio)methyl-1*H*-1,2,3-triazol-4-yl)methoxy-12*H*-quino[3,4-*b]*[1,4]benzothiazinium chloride**2l** in CD_3OD

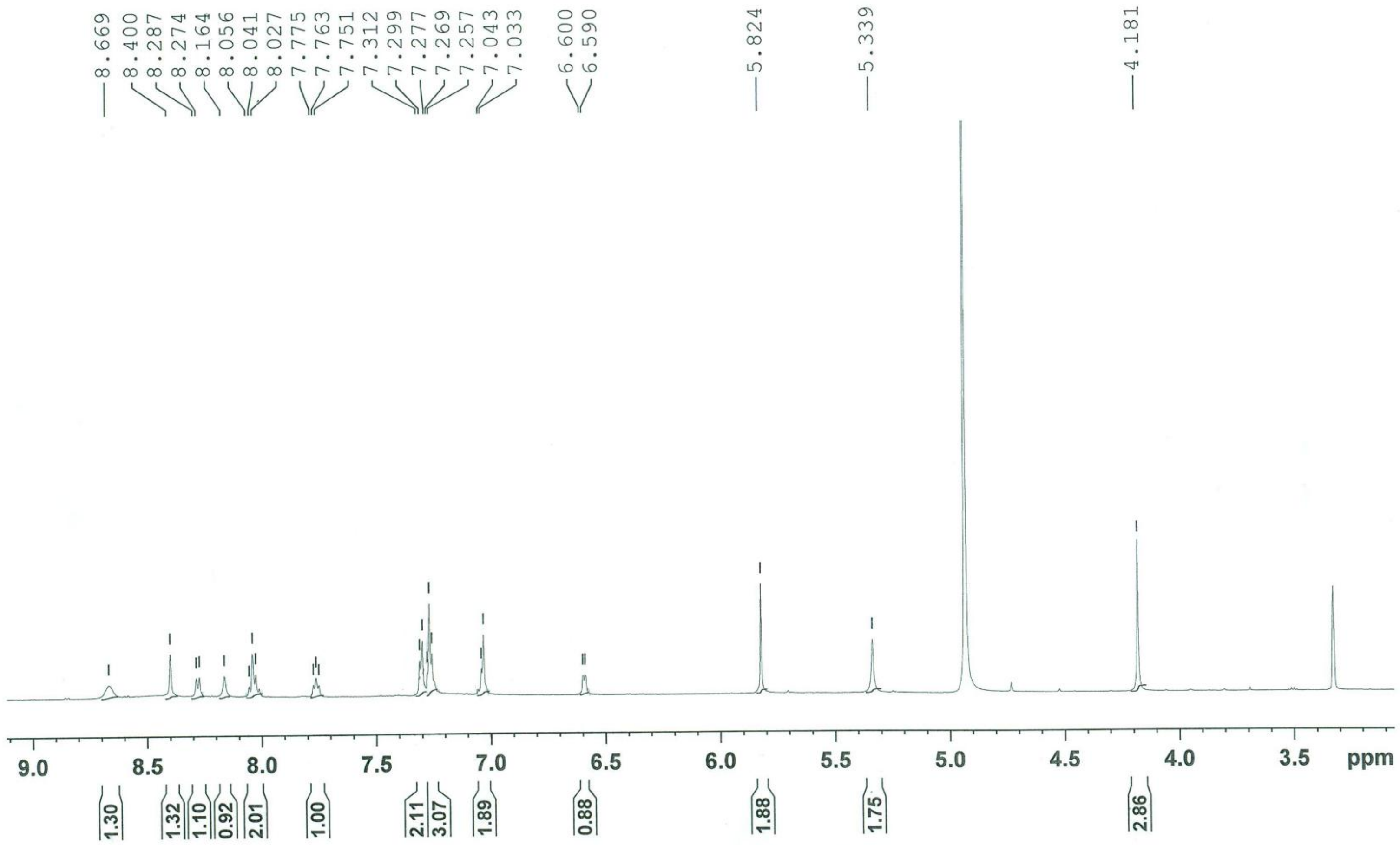

Fig. S59 ^{1}H NMR Spectrum of 5-Methyl-9-(1-(4-chlorophenyl)-1*H*-1,2,3-triazol-4-yl)methoxy-12*H*-quino[3,4-*b*][1,4]benzothiazinium chloride **2m** in DMSO

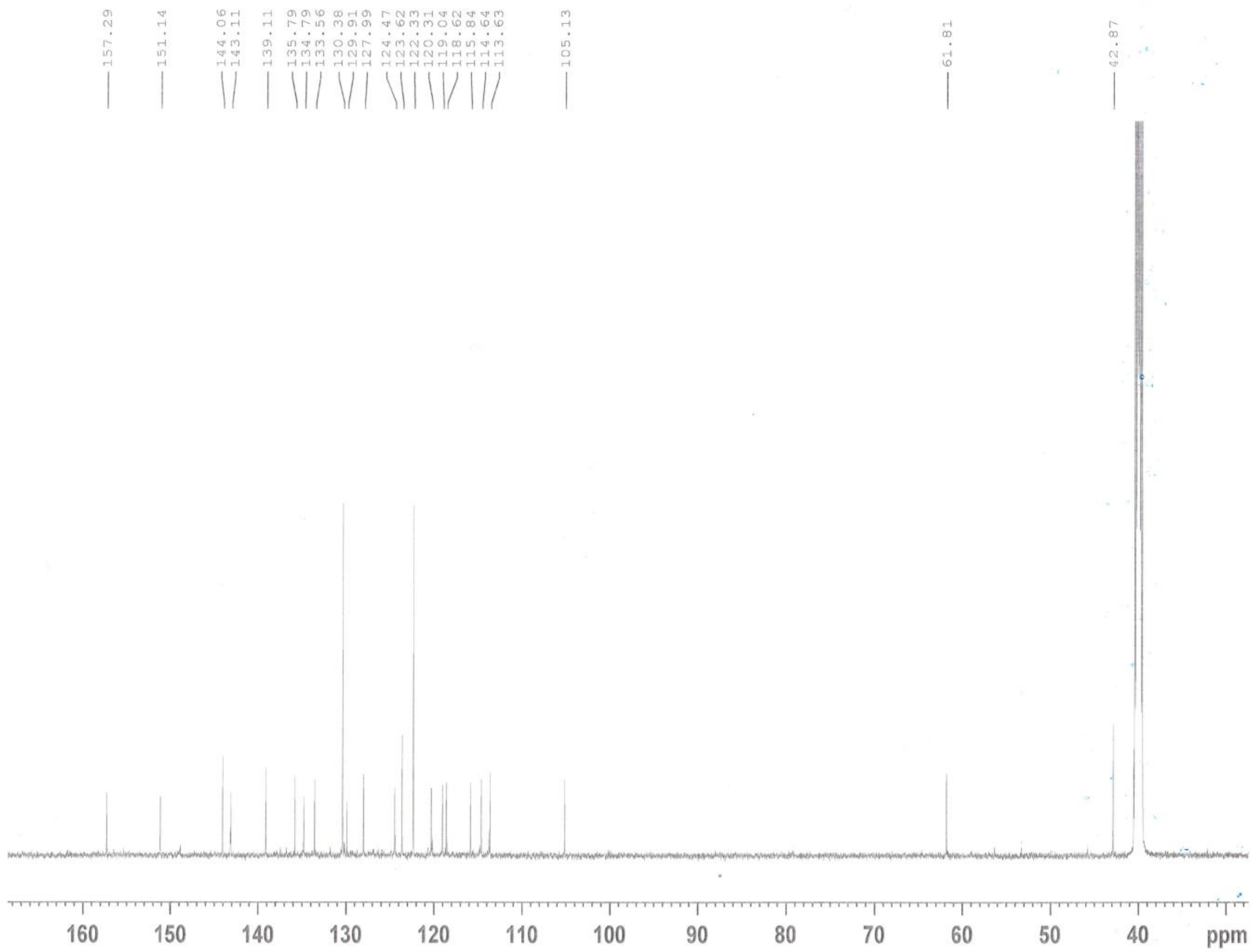

Fig. S60 ^{13}C NMR Spectrum 5-Methyl-9-(1-(4-chlorophenyl)-1*H*-1,2,3-triazol-4-yl)methoxy-12*H*-quino[3,4-*b*][1,4]benzothiazinium chloride **2m** in DMSO.

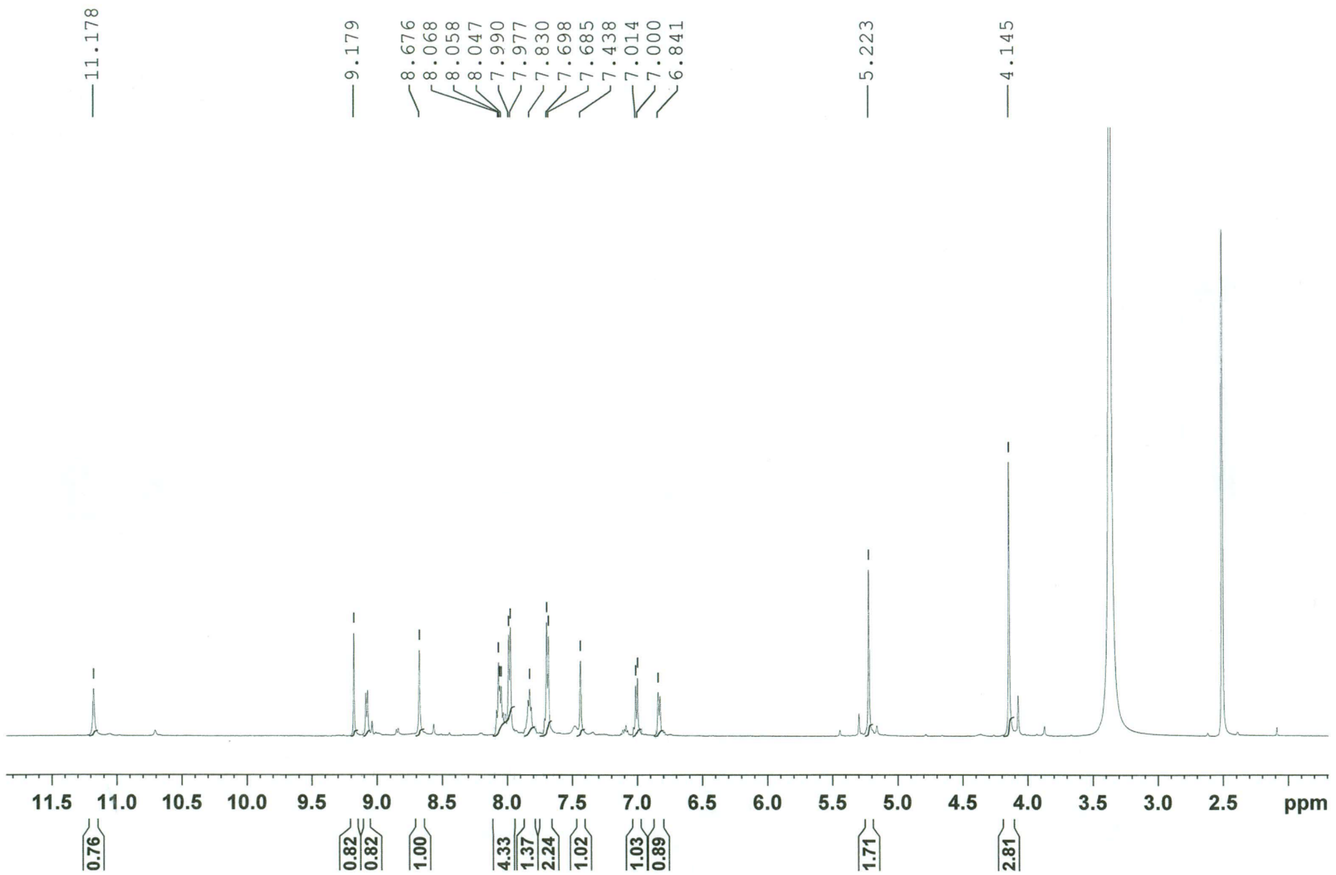

Fig. S61 ^{1}H NMR Spectrum 5-Methyl-10-(1-(4-chlorophenyl)-1*H*-1,2,3-triazol-4-yl)methoxy-12*H*-quino[3,4-*b*][1,4]benzothiazinium chloride **2n** in DMSO.

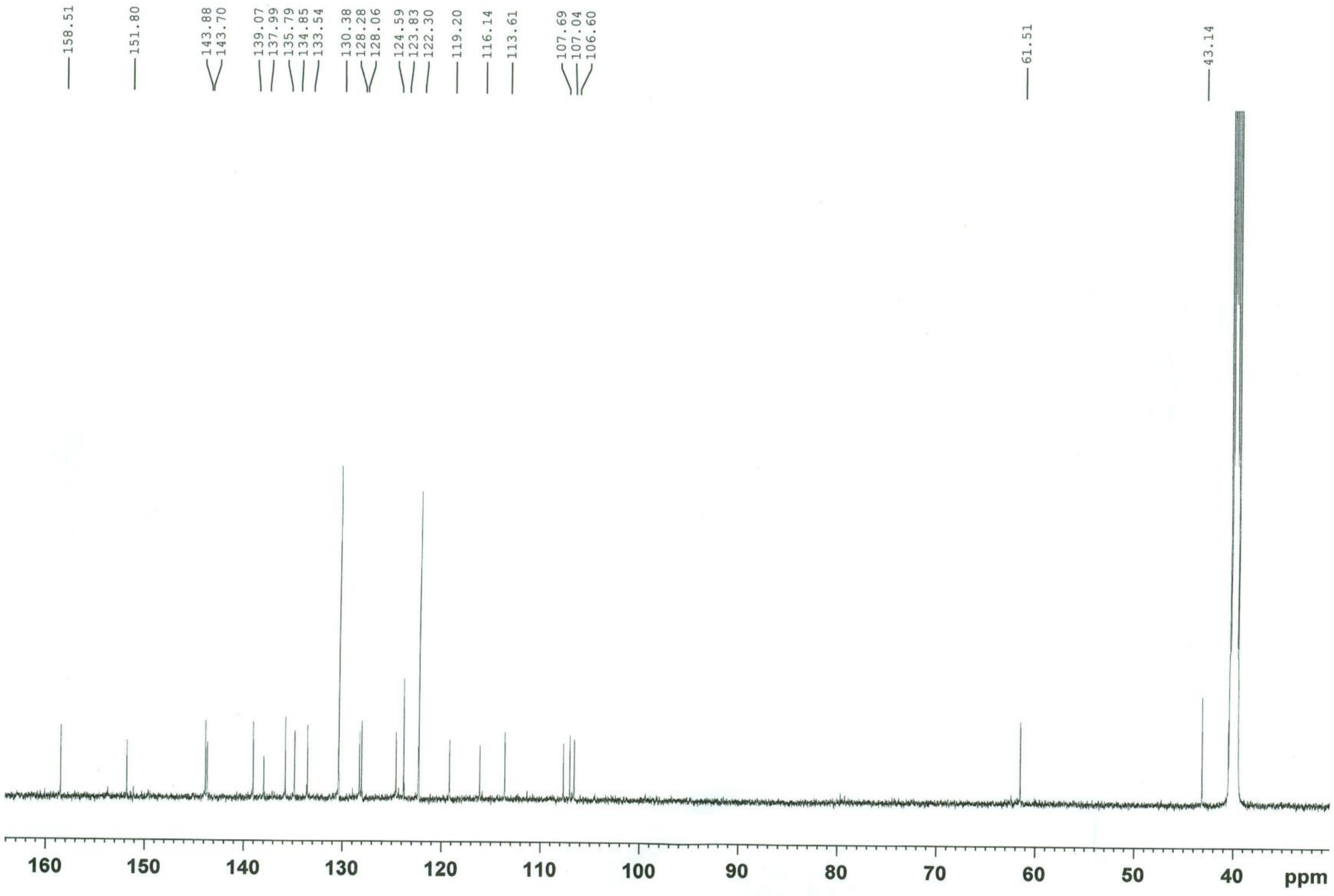

Fig. S62 ^{13}C NMR Spectrum 5-methyl-10-(1-(4-chlorophenyl)-1*H*-1,2,3-triazol-4-yl)methoxy-12*H*-quino[3,4-*b*][1,4]benzothiazinium chloride 2**n** in DMSO
